# The chemical compound ‘Heatin’ stimulates hypocotyl elongation and interferes with the Arabidopsis NIT1‐subfamily of nitrilases

**DOI:** 10.1111/tpj.15250

**Published:** 2021-05-06

**Authors:** Lennard van der Woude, Markus Piotrowski, Gruson Klaasse, Judith K. Paulus, Daniel Krahn, Sabrina Ninck, Farnusch Kaschani, Markus Kaiser, Ondřej Novák, Karin Ljung, Suzanne Bulder, Marcel van Verk, Basten L. Snoek, Martijn Fiers, Nathaniel I. Martin, Renier A. L. van der Hoorn, Stéphanie Robert, Sjef Smeekens, Martijn van Zanten

**Affiliations:** ^1^ Molecular Plant Physiology Institute of Environmental Biology Utrecht University Padualaan 8 Utrecht 3584 CH the Netherlands; ^2^ Department of Molecular Genetics and Physiology of Plants Faculty of Biology and Biotechnology Universitätsstraße 150 Bochum 44801 Germany; ^3^ Department of Chemical Biology & Drug Discovery Utrecht Institute for Pharmaceutical Sciences University Utrecht Universiteitsweg 99 Utrecht 3584 CG the Netherlands; ^4^ Plant Chemetics Laboratory Department of Plant Sciences University of Oxford South Parks Road Oxford OX1 3RB UK; ^5^ Chemische Biologie Zentrum für Medizinische Biotechnologie Fakultät für Biologie Universität Duisburg‐Essen Universitätsstr. 2 Essen 45117 Germany; ^6^ Umeå Plant Science Centre Department of Forest Genetics and Plant Physiology Swedish University of Agricultural Sciences Umea SE‐901 83 Sweden; ^7^ Laboratory of Growth Regulators The Czech Academy of Sciences & Faculty of Science Institute of Experimental Botany Palacký University Šlechtitelů 27 Olomouc 78371 Czech Republic; ^8^ Bejo Zaden B.V. Trambaan 1 Warmenhuizen 1749 CZ the Netherlands; ^9^ Plant‐Microbe Interactions Institute of Environmental Biology Utrecht University Padualaan 8 Utrecht 3584 CH the Netherlands; ^10^ Keygene Agro Business Park 90 Wageningen 6708 PW the Netherlands; ^11^ Theoretical Biology and Bioinformatics Institute of Biodynamics and Biocomplexity Utrecht University Padualaan 8 Utrecht 3584 CH the Netherlands; ^12^ Bioscience Wageningen University and Research Droevendaalsesteeg 1 Wageningen 6708 PB the Netherlands; ^13^ Biological Chemistry Group Sylvius Laboratories Institute of Biology Leiden Leiden University Sylviusweg 72 Leiden 2333 BE the Netherlands

**Keywords:** chemical genetics, thermomorphogenesis, Arabidopsis, nitrilases, NIT1‐subfamily, Heatin, aldehyde oxidase, PIF4, 1‐iminomethyl‐2‐naphthol, IAN, indole‐3‐acetonitrile

## Abstract

Temperature passively affects biological processes involved in plant growth. Therefore, it is challenging to study the dedicated temperature signalling pathways that orchestrate thermomorphogenesis, a suite of elongation growth‐based adaptations that enhance leaf‐cooling capacity. We screened a chemical library for compounds that restored hypocotyl elongation in the *pif4‐2*–deficient mutant background at warm temperature conditions in *Arabidopsis thaliana* to identify modulators of thermomorphogenesis. The small aromatic compound ‘Heatin’, containing 1‐iminomethyl‐2‐naphthol as a pharmacophore, was selected as an enhancer of elongation growth. We show that *ARABIDOPSIS ALDEHYDE OXIDASES* redundantly contribute to Heatin‐mediated hypocotyl elongation. Following a chemical proteomics approach, the members of the NITRILASE1‐subfamily of auxin biosynthesis enzymes were identified among the molecular targets of Heatin. Our data reveal that nitrilases are involved in promotion of hypocotyl elongation in response to high temperature and Heatin‐mediated hypocotyl elongation requires the NITRILASE1‐subfamily members, NIT1 and NIT2. Heatin inhibits NIT1‐subfamily enzymatic activity *in vitro* and the application of Heatin accordingly results in the accumulation of NIT1‐subfamily substrate indole‐3‐acetonitrile *in vivo*. However, levels of the NIT1‐subfamily product, bioactive auxin (indole‐3‐acetic acid), were also significantly increased. It is likely that the stimulation of hypocotyl elongation by Heatin might be independent of its observed interaction with NITRILASE1‐subfamily members. However, nitrilases may contribute to the Heatin response by stimulating indole‐3‐acetic acid biosynthesis in an indirect way. Heatin and its functional analogues present novel chemical entities for studying auxin biology.

## Significance Statement

We identified the chemical compound Heatin that stimulates plant thermomorphogenesis and show that Heatin interferes with NIT1‐subfamily enzymatic activity and results in accumulation of auxin. The precise mechanism of action of Heatin requires further study but the investigations presented can contribute to knowledge‐based engineering of warm temperature‐resilient crops. Heatin is a promising agrochemical to steer plant growth and is a novel chemical tool for studying auxin biology.

## INTRODUCTION

Many plant species, including the model system *Arabidopsis thaliana*, respond to small increases in temperature by adapting their architecture to maximize fitness in suboptimal temperature conditions. This process is called thermomorphogenesis (Casal and Balasubramanian, 2019; Quint *et al.*, 2016) and comprises a number of growth features including hypocotyl elongation, upward leaf movement and petiole elongation. The resulting open rosette architecture promotes cooling capacity (Crawford *et al.*, 2012; Park *et al.*, 2019). Temperature is perceived via diverse molecular mechanisms that are increasingly well understood (Chung *et al.*, 2020; Jung *et al.*, 2020; Jung *et al.*, 2016; Legris *et al.*, 2016). Central in the regulation of thermomorphogenesis are bHLH transcription factor proteins PHYTOCHROME INTERACTING FACTORS 4 (PIF4) and PIF7 (Chung *et al.*, 2020; Fiorucci *et al.*, 2020; Franklin *et al.*, 2011; van der Woude *et al.*, 2019). Levels of these PIFs increase rapidly in response to high temperature (Chung *et al.*, 2020; Fiorucci *et al.*, 2020; Franklin *et al.*, 2011; van der Woude *et al.*, 2019) and PIFs induce biosynthesis of the bioactive auxin indole‐3‐acetic acid (IAA) that is required for thermomorphogenesis by binding and activating the promoters of key auxin biosynthesis genes such as *TRYPTOPHAN AMINOTRANSFERASE OF ARABIDOPSIS* (*TAA1*) and the *cytochrome P450s* genes, *CYP79B2* and *CYP79B3*, as well as the flavin monooxygenase *YUCCA8* (*YUC8*) (Chung *et al.*, 2020; Fiorucci *et al.*, 2020; Franklin *et al.*, 2011; Sun *et al.*, 2012; van der Woude *et al.*, 2019).

Substantial progress has been made in recent years in our understanding of the molecular and genetic regulation of thermomorphogenesis (Casal and Balasubramanian, 2019; Quint *et al.*, 2016). However, functional redundancy among temperature sensing and response mechanisms, as well as the thermodynamic (passive) effect of temperature on biological processes, impeded the identification of factors involved in the complex molecular networks regulating temperature‐mediated elongation growth, as these are readily missed by classical forward genetics approaches (Vu *et al.*, 2019). Alternative strategies to identify possible redundant factors are chemical genetics or chemical genomics approaches (Dejonghe and Russinova, 2017; Kaschani and van der Hoorn, 2007). In chemical genetics, compound libraries are screened for compounds that interfere with the biological system. One major advantage is that compounds can target a broad range of related molecules, whereas knockout mutations in a single gene could be masked by redundancy. Moreover, compounds can be more specific than genetic loss‐of‐function approaches in terms of tissue localization or developmental timing of its effects (Cutler and McCourt, 2005; Hicks and Raikhel, 2014; Kaschani and van der Hoorn, 2007; Park *et al.*, 2009). Chemical genetics has its origin in the pharmaceutical sciences where it is used to identify targets of known drugs and for drug discovery (Cong *et al.*, 2012). Prominent examples in plant sciences include the identification of abscisic acid receptor proteins (Park *et al.*, 2009) and studies on biosynthesis and functioning of auxin and brassinosteroids (Nishimura *et al.*, 2014; Savaldi‐Goldstein *et al.*, 2008; Zhao *et al.*, 2003).

Here, a chemical genetics approach was taken to study thermomorphogenesis by screening for small molecules that rescue impaired hypocotyl elongation of the *pif4‐2* mutant at high temperature conditions. We identified a compound, named ‘Heatin’, containing a 1‐iminomethyl‐2‐naphthol moiety that was subsequently determined to be the pharmacophore. A targeted chemical proteomics approach using a chemically tagged Heatin analogue led to the identification of members of the NITRILASE1 (NIT1)‐subfamily among the Heatin targets. We demonstrate by genetic means that the NIT1‐subfamily of nitrilases promotes hypocotyl elongation in response to high temperature and that Heatin requires these enzymes for its effect on hypocotyl elongation. Heatin was found to inhibit NIT1‐subfamily enzyme activity *in vitro* and Heatin treatment resulted in *in vivo* accumulation of the substrate indole‐3‐acetonitrile (IAN). Surprisingly, also a significant accumulation of bioactive auxin (IAA) was detected in Heatin‐treated seedlings. Therefore, the stimulation of hypocotyl elongation by Heatin appears to involve other actors in addition to nitrilases. The role of nitrilases in the production of IAA is not well‐understood and Heatin somehow impinges on this role.

## RESULTS

### Identification of Heatin as compound that stimulates thermomorphogenesis

**Figure 1:**
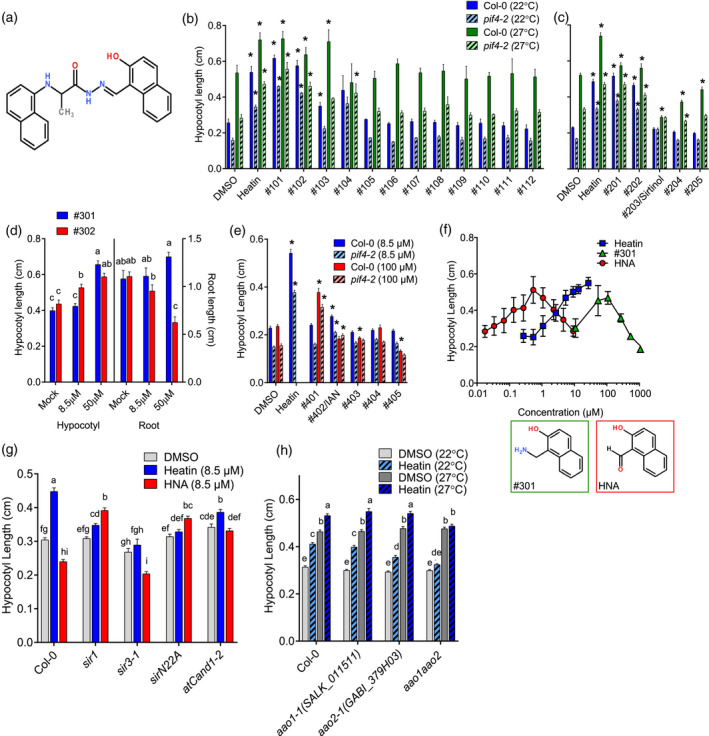
Structure–activity relation study. (a) Heatin structure (*N*'‐[(2‐hydroxy‐1‐naphthyl)methylene]‐2‐(1‐naphthylamino)propanohydrazide). (b–e) Effects of Heatin analogues (8.5 µm, unless specified otherwise) on hypocotyl elongation and (d) root length, of 8‐day‐old Col‐0 wild‐type (dark, clear bars) or *pif4‐2* mutant (light, dashed bars) seedlings, at (b,c) 22°C (blue bars) or 27°C (green bars). (d) Dose–response relationship of Heatin analogue no. 301 (blue bars) and no. 302 (red bars) on hypocotyl elongation (left panel) and root length (right panel). (e) Dose–response relationship of Heatin analogues on Col‐0 and *pif4‐2* applied at 8.5 µm (blue bars) or 100 µm (red bars), at 22°C. DMSO, dimethyl sulphoxide. (f) Dose–response relationship of Heatin (blue, squares), 1‐aminomethyl‐2‐naphthol (no. 301; green, triangles) and 2‐hydroxy‐1‐naphthaldehyde (HNA, red circles) on hypocotyl elongation of 8‐day‐old Col‐0 seedlings. Insets below panel indicate compound no. 301 (left, green line) and HNA (right, red line) structures. (g,h) Hypocotyl lengths of 8‐day‐old (g) Sirtinol‐resistant *arabidopsis aldehyde oxidase 1* (*aao1*), *aao2* and *aao1 aao2* double mutants, in the presence of mock (DMSO; grey open bars), Heatin (8.5 µm; blue bars), or HNA (8.5 µm; red bars) at (g,h) 22°C (light bars) or (h) 27°C (dark bars). Values are averages of (b,c,e,f,g,h) three or four replicates, of 15–25 seedlings each, or (d) a representative experiment of 20 seedlings. (b,c,e) Asterisks indicate significant differences (*P* < 0.05) from wild type. (d,g,h) Letters indicate significance groups (Tukey HSD *post hoc*), where averages that do not share letters are significantly different from each other (*P* < 0.05). Error bars indicate SEM.

Small molecule compounds that potentially stimulate thermomorphogenesis were identified from a library of 8360 chemical entities, by testing their ability to restore hypocotyl elongation of the *Arabidopsis pif4‐2* mutant grown at warm temperature (28°C) (Figure S1a,b, Table S1). In total, 36 initial compounds were identified that stimulated elongation growth without apparent visual side effects such as curled leaves or agravitropic growth (Figure S1b,c, Table S2). Quantification of hypocotyl lengths at control (22°C) and high temperature (27°C) conditions reproducibly confirmed the stimulatory effects of several compounds in either Col‐0 wild type and/or *pif4‐2* (Table S3, Figure 1c).

One compound (D; Figure 1a, Table S3) stood out for its strong elongation‐stimulating capacity (Figure 1, Figure S1c). Therefore, we further focused on this compound, subsequently named ‘Heatin’. To test if Heatin can stimulate thermomorphogenesis, we verified if it could stimulate thermomorphogenesis‐related phenotypes in rosette‐stage plants (Figure S2a). A significant Heatin effect was indeed found for all measured architectural traits except petiole length (Figure S2b–h) in both Col‐0 and *pif4‐2* mutant plants. This shows that Heatin treatment phenocopies thermomorphogenesis and acts at least partly genetically downstream of PIF4 or bypasses PIF4 effects.

### 1‐Iminomethyl‐2‐naphthol is a pharmacophore for Heatin

Structure–activity relationship assessment was performed to identify the key active moiety of Heatin (Figure 1b; Figure S3a–e, Table S4). First, 12 commercially available Heatin analogues, assigned nos 101–112, were tested (Figure 1b). These compounds all had a similar central core structure and differed mainly in the composition of their side chains (Figure S3b, Table S4). Four of these compounds were bioactive (nos 101–104; Figure 1b) and all these contained a 2‐naphthol moiety that was absent in non‐active compounds (Figure S3b). Of note, compound no. 104 was previously identified in a chemical genetics screen to affect G‐protein‐dependent signalling in rat cell cultures (Marlo *et al.*, 2009). Therefore, we tested various Arabidopsis G‐protein signalling mutants but did not find any significant effect that would suggest their involvement in Heatin signalling (Figure S4a).

Subsequently, a second set of compounds was assayed (nos 201–205), which had different degrees of similarity to the central Heatin core structure, but all contained the 2‐naphthol moiety (Figure S3c, Table S4). Neither 2‐naphthol (no. 205) itself, nor 1‐methyl 2‐napthol (no. 204), were sufficient for hypocotyl elongation, even at high concentrations (Figure 1c; Figure S4b). Compound nos 201, 202 and 203 were active (Figure 1c), although no. 203 inhibited hypocotyl elongation under high temperature conditions. These three bioactive compounds contained either 1‐aminomethyl‐2‐naphthol (no. 202) or 1‐iminomethyl‐2‐naphthol (nos 201 and 203) moiety, which were absent in inactive compound nos 204 and 205 (Figure 1c). Therefore, the 1‐aminomethyl‐2‐naphthol/1‐iminomethyl‐2‐naphthol moiety is considered the pharmacophore for hypocotyl elongation. Indeed, 1‐aminomethyl‐2‐naphthol (no. 301) by itself was able to induce hypocotyl elongation (Figure 1d; Figure S3d, Table S4), albeit at higher concentrations than used to identify Heatin (8.5 µm).

To assess the requirement of the hydroxyl‐group of 1‐aminomethyl‐2‐naphthol for bioactivity or specificity, 1‐naphthylmethylamine (no. 302) was assayed in parallel. This compound caused hypocotyl elongation at high concentrations, e.g. 1‐aminomethyl‐2‐naphthol (no. 301), although a strong inhibitory effect on root growth was also observed (Figure 1d; Figure S3d, Table S4), suggesting that the hydroxyl group of 1‐aminomethyl‐2‐naphthol, and most likely the 1‐iminomethyl‐2‐naphthol pharmacophore for Heatin is necessary for the specificity of hypocotyl elongation during seedling growth.

Further evaluation of compounds with additional structural features (nos 401–405) (Figure 1e; Figure S3e, Table S4) revealed that modifications between the 2‐napthol and the amide group generally led to reduced bioactivity compared with Heatin. However, changing the amino‐group into a piperidine (no. 401) led to retention of some bioactivity at high concentrations (Figure 1e). Of note, treatment with the Heatin analogue, and auxin precursor, IAN (no. 402) stimulated hypocotyl elongation, albeit less effectively than Heatin (Figure 1e) (Normanly *et al.*, 1997).

### *ARABIDOPSIS ALDEHYDE OXIDASES* redundantly contribute to Heatin‐mediated hypocotyl elongation

Heatin analogue no. 203 was identified previously as Sirtinol, a compound that inhibits Sirtuin NAD‐dependent deacetylases in yeast (Grozinger *et al.*, 2001). In Arabidopsis, Sirtinol supresses hypocotyl elongation in darkness (Zhao *et al.*, 2003). We found that Sirtinol (no. 203) also supresses high temperature‐induced hypocotyl elongation in light (Figure 1c), despite Sirtinol containing the 1‐iminomethyl‐2‐naphthol pharmacophore (Figure S3c) that was sufficient for stimulation of hypocotyl elongation in Heatin and its analogues (Figure 1b,f).

Previous structure–activity relationship studies identified 2‐hydroxy‐1‐naphthaldehyde (HNA) as the pharmacophore for Sirtinol, which is similar to the 1‐iminomethyl‐2‐naphthol pharmacophore for Heatin (Figure 1f), but is hydrolysed on the amide (Dai *et al.*, 2005). Considering the suppression of hypocotyl elongation by Sirtinol (no. 203) (Figure 1c) and the concentration‐dependent manner of 1‐aminomethyl‐2‐naphthol (no. 301)‐mediated hypocotyl elongation (Figure 1d), we assayed HNA effects over a concentration range. This revealed that HNA was able to stimulate hypocotyl elongation, but at much lower concentrations than previously used by Dai *et al.*, (2005) and Zhao *et al.*, (2003) (Figure 1f).

Several *sirtinol resistant* (*sir*) mutants are available and most have defects in molybdenum cofactor biosynthesis (Dai *et al.*, 2005; Zhao *et al.*, 2003). The current hypothesis is that HNA is metabolized into bioactive 2‐hydroxy‐1‐naphthoic acid (HNC) by molybdenum cofactor‐dependent aldehyde oxidase activity. In addition, we observed that Sirtinol‐resistant mutants are resistant to applications of Heatin (Figure 1g). Expression data from the TraVA database indicated that of the four *ARABIDOPSIS ALDEHYDE OXIDASE* (*AAO)* genes present in Arabidopsis, *AAO1* and *AAO2* transcripts are most abundant in young seedling tissues (Klepikova *et al.*, 2016) (Figure S5a). Therefore, we generated an *aao1 aao2* double mutant (Figure S5b–d) and found that this line is insensitive to Heatin (Figure 1h), whereas high temperature‐induced hypocotyl elongation was retained. Various *aao* single mutants remained sensitive to Heatin application (Figure 1h; Figure S5d), except for *aao2* that consistently exhibited reduced sensitivity. These findings suggest that AAO1 and AAO2 redundantly contribute to Heatin‐mediated hypocotyl elongation, with a possible prevalent role for AAO2.

### Heatin‐mediated hypocotyl elongation involves auxin

Thermomorphogenesis depends on bioactive auxin and the structure of the endogenous auxin precursor IAN (no. 402) is similar to 1‐iminomethyl‐2‐naphthol. Moreover, a role for AAO2 in auxin biosynthesis has been implicated (Seo *et al.*, 1998), although this is disputed (Mashiguchi *et al.*, 2011; Seo *et al.*, 2004). Therefore, we investigated a possible relation between auxin and Heatin in mediating hypocotyl elongation.

**Figure 2:**
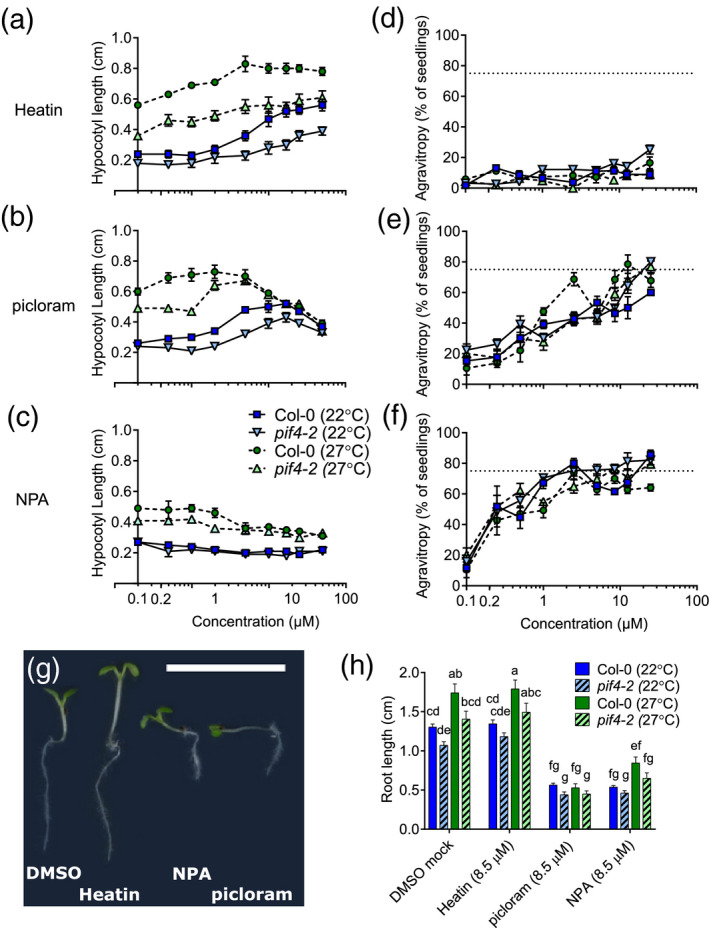
Dose–response effects of Heatin, picloram and *N*‐1‐naphthylphthalamic acid (NPA) on hypocotyl elongation, gravitropism and root length. (a–c) Hypocotyl lengths and (d–f) agravitropy scores, of 8‐day‐old Col‐0 wild‐type (circles and squares) and *pif4‐2* (triangles) seedlings in the presence of (a,d) Heatin, (b,e) picloram and (c,f) NPA at 22°C (dark square, triangles down markers, closed lines) or 27°C (circles and triangles up, light markers, dashed lines). (d,f) Dashed horizontal lines at 75% indicate complete agravitropy (random seedling orientation). (g) Image of representative seedlings grown on the indicated compounds (8.5 µm). Note the differences in hypocotyl lengths and gravitropism. Scale bar = 1 cm. (h) Root length of 8‐day‐old Col‐0 wild‐type (clear bars) and *pif4‐2* (dashed bars) seedlings grown on mock (dimethyl sulphoxide solvent), Heatin, picloram or NPA (each 8.5 µm), at 22°C (blue bars) or 27°C (green bars). Letters indicate significance groups (Tukey HSD *post‐hoc* tests), where averages that do not share letters are significantly different from each other (*P* < 0.05). Values are averages of three replicates of 15–25 seedlings each. Error bars indicate SEM.

The application of Heatin triggered hypocotyl elongation in a dose–response way (Figures 1f and 2a) in Col‐0 wild‐type and *pif4‐2* mutant plants at control (22°C) and high (27°C) temperature conditions, in a manner distinct from the synthetic auxin analogue picloram (4‐amino‐3,5,6‐trichloro‐2‐pyridinecarboxylic acid) (Figure 2b). The polar auxin transport inhibitor *N*‐1‐naphthylphathalamic acid (NPA) suppressed elongation growth (Figure 2c) as expected, but Heatin treatment could overcome this inhibition (Figure S6a). Picloram becomes repressive for hypocotyl elongation at high concentrations (Figure 2b) but such a negative effect was not observed for Heatin (Figure 2a).

NPA application, or saturation of the tissues with picloram, disturbs coordinated gravity‐directed growth (gravitropic response (Boonsirichai *et al.*, 2002; Nagashima *et al.*, 2008; Rakusová *et al.*, 2011), Figure 2e–g). In contrast, Heatin did not interfere with gravitropism (Figure 2d,g). Consistent with published data, we observed that high temperature results in longer roots, whereas roots of *pif4‐2* were mildly shorter than wild type (Figure 2h) (Martins *et al.*, 2017). Heatin and compound 1‐aminomethyl‐2‐naphthol (no. 301) did not affect root length, unlike NPA and picloram that were both inhibitive (Figures 1d and 2h).

**Figure 3:**
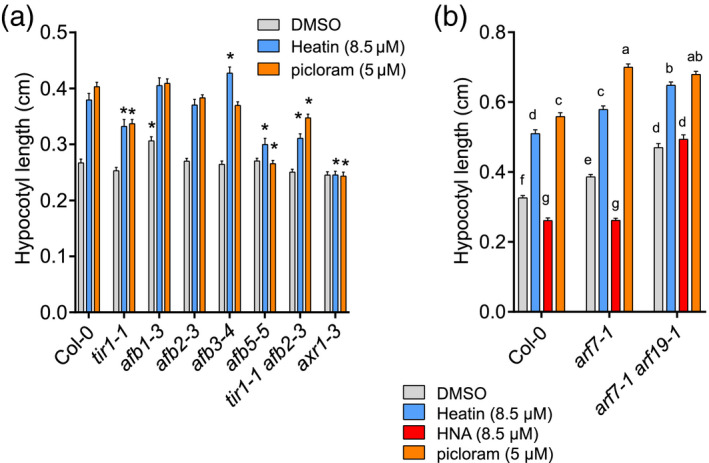
Heatin activity is dependent on auxin. (a,b) Hypocotyl lengths of 8‐day‐old wild‐type Col‐0 and auxin signalling mutant seedlings, on mock (DMSO solvent, grey bars) or in the presence of (a,b), Heatin (8.5 µm; blue bars), picloram (a) 5 µm or (b) 8.5 µm (light orange bars), and (b) HNA (red bars). (a) Asterisks indicate significant difference from Col‐0 wild type (*P* < 0.05). (b) Letters indicate significance groups (Tukey HSD *post‐hoc* test), where averages that do not share letters are significantly different from each other (*P* < 0.05). Error bars indicate SEM. Values are averages of three to five independent repetitions of 15–25 seedlings each. DMSO, dimethyl sulphoxide; HNA, 2‐hydroxy‐1‐naphthaldehyde.

The *axr1‐3* mutant, disturbed in SCF^TIR1/AFB^ auxin receptor complex formation (del Pozo *et al.*, 2002; del Pozo and Estelle, 1999), was insensitive to Heatin (Figure 3a; Figure S6b). The *tir1‐1* receptor mutant showed reduced Heatin sensitivity as well, whereas mutants in auxin receptors, AFB1 and AFB2, retained responsiveness at 8.5 µm Heatin. Surprisingly, *afb3‐4* was hypersensitive to Heatin (Figure 3a). The double *tir1‐1 afb2‐3* mutant was more resistant than the single mutants were. The *afb5‐5* mutant was insensitive to picloram, but remained significantly, albeit reduced, responsive to Heatin (Figure 3a; Figure S6b). Together, these data suggest that Heatin activity relies on intact auxin perception genes and that genetic redundancy between different auxin receptors exists in relaying the Heatin signal.

Several mutants disturbed in auxin signalling, such as the *arf7‐1 arf19‐1* double mutant (Li *et al.*, 2006) and *AtCAND1*, involved in SCF‐complex functioning (Cheng *et al.*, 2004), exhibited altered sensitivity to HNA. The *Atcand1‐2* mutant was resistant to Heatin as well (Figure 1g). However, *arf7‐1* and *arf7‐1 arf19‐1* mutants retained sensitivity to Heatin application (Figure 3b; Figure S6c). Furthermore, where Sirtinol is known to activate *DR5:GUS* (Zhao *et al.*, 2003), Heatin was unable to induce this auxin reporter (Figure S6d). Together, this suggests that Sirtinol and Heatin affect at least in part, different signalling pathways, despite the structural similarities.

### Transcriptomics of Heatin responsiveness

Time‐lapse imaging of hypocotyl growth demonstrated that Heatin‐induced hypocotyl elongation initiated approximately 48 h after induction of germination, during the photoperiod of day 3 (Figure S7a,b). This contrasts with the onset of high temperature‐induced hypocotyl elongation, which occurred from germination (*t* = approximately 24 h) onwards. Heatin effects persisted throughout the experimental period. Both *pif4‐2* and Col‐0 wild‐type seedlings were sensitive to Heatin (Figure S8), confirming that PIF4 is not essential for Heatin’s effects. However, as *pif4‐2* mutants appear less responsive (Figure 2a; Figure S11), we cannot exclude that PIF4 contributes to the effects of Heatin. Based on these findings, we defined an early (2‐day‐old seedlings; 48 h) and late (7‐day‐old seedlings; 168 h) sampling time point for RNA‐sequencing (RNA‐seq) experiments, with the aim of cataloguing Heatin‐induced changes in the transcriptome (see Appendix S1 for details).

Upon application of Heatin, the expression of only two genes at 22°C and 10 genes at 27°C was significantly different in 2‐day‐old seedlings. The effects of Heatin were more pronounced in 7‐day‐old seedlings (Tables S5–S7, Figure S9, Appendix S1). The small number of genes affected by Heatin treatment in 2‐day‐old seedlings hampered systematic enrichment analyses. However, four of the eight Heatin‐upregulated genes in 2‐day‐old seedlings at 27°C are known auxin responsive genes [*INDOLE‐3‐ACETIC ACID INDUCIBLE 5* (*IAA5*), *GH3.1*, *GH3.3* and *1‐AMINOCYCLOPROPANE‐1‐CARBOXYLATE SYNTHASE 4* (*ACS4*)] (Paponov *et al.*, 2008; Tian *et al.*, 2002) (Table S6). Terms in the Gene Ontology (GO) enrichment analysis of Heatin‐upregulated genes at day 7 also pointed towards auxin, as among other terms, genes annotated as ‘response to auxin stimulus’ was found as an overrepresented category (22°C) (Table S8). Examination of specific auxin biosynthesis and signalling genes confirmed that expression of several AUX/IAA proteins is significantly affected by Heatin treatment, whereas auxin biosynthesis genes do not seem affected at the transcript level (Table S9).

### NIT1‐subfamily proteins are potential molecular targets of Heatin

**Figure 4:**
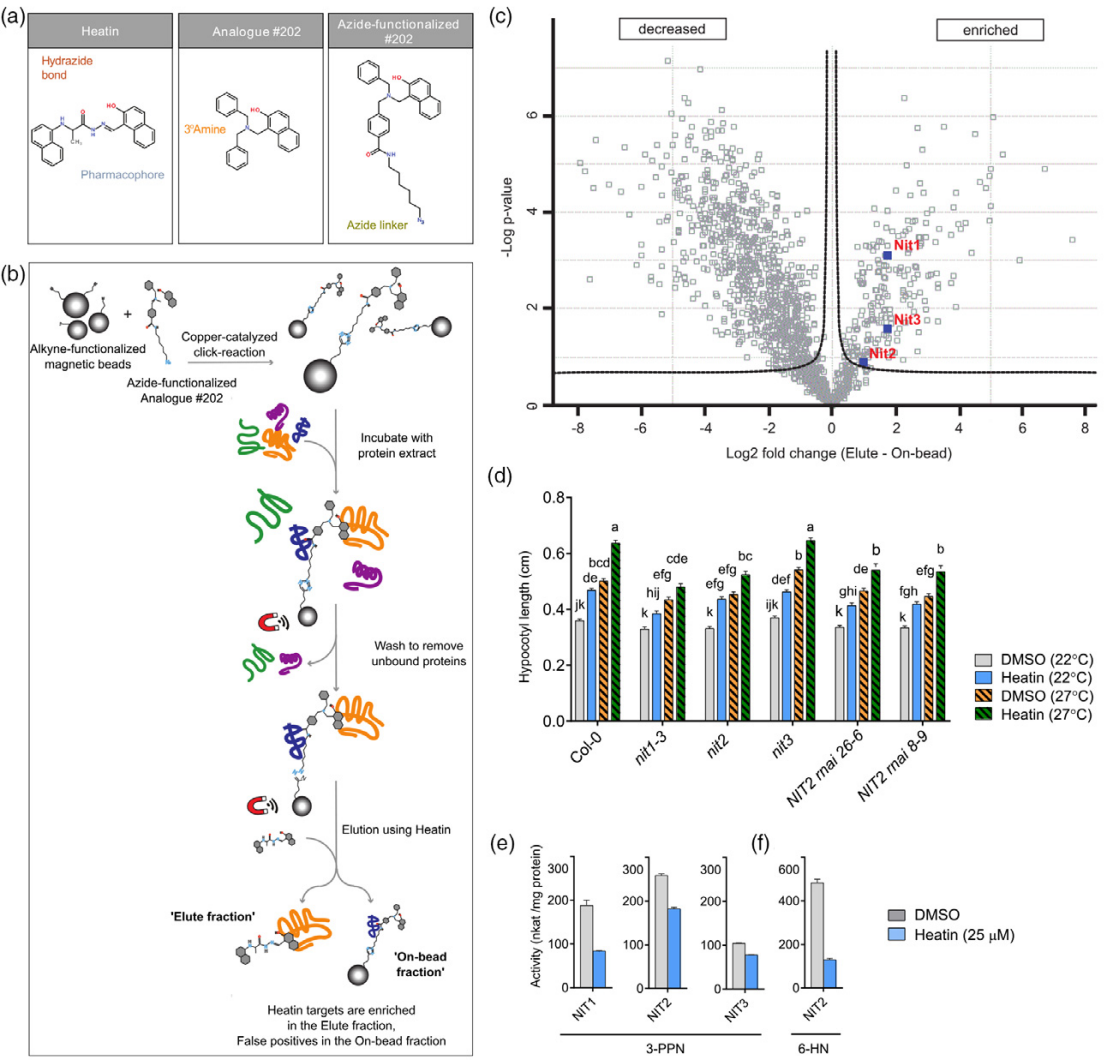
Nitrilase 1‐subfamily members are direct Heatin targets. (a) Chemical structures of Heatin (left), analogue no. 202 (middle) and azide‐functionalized compound no. 202 (right). Highlighted are the pharmacophore (blue), Heatin’s hydrazide bond (red), analogue no. 202’s corresponding amine bond (yellow) and the azide linker (green). (b) Schematic representation of the chemical proteomics strategy. (c) Volcano plot of statistical significance against fold‐change of protein group label‐free quantification intensities between ‘Heatin‐eluted’ and ‘On‐bead fractions’ based on a two‐sided Student’s *t*‐test (FDR: 0.05; S0: 0.1). NIT1‐subfamily member proteins (indicated in blue squares and red letters) are enriched in the Elute fraction. Dotted lines represent the threshold for significant differences in protein abundances. Data are based on four biological replicates per condition. (d) Hypocotyl lengths of 8‐day‐old *nitrilase1‐subfamily* mutant seedlings and Col‐0 wild type, grown on mock [dimethyl sulphoxide (DMSO), grey and orange bars] or in presence of Heatin (8.5 µm; blue and green bars), at either 22°C (open bars) or 27°C (dashed bars). Values are averages of six independent repetitions of 20–30 seedlings each. Letters indicate significance groups (Tukey HSD *post‐hoc* test), where averages that do not share letters are significantly different from each other (*P* < 0.05). (e,f) *In vitro* enzymatic activity of recombinant NIT1‐subfamily proteins with (e) 3‐phenylpropionitrile (3‐PPN) or (f) 6‐heptenenitrile (6‐HN) as substrate (2.5 µm), with DMSO solvent as mock (grey bars) or Heatin (25 µm; blue bars), present in the reaction mix. Values are averages of three technical replicates. Error bars indicate SEM.

A chemical proteomics (Futamura *et al.*, 2013) strategy was designed to identify potential biomolecular targets of Heatin (Figure 4; Figures S10–S12a). For this purpose, we structurally elaborated Heatin analogue no. 202 (Figure S3c) as it displayed a similar bioactivity (Figure 1c) to Heatin and shares the 2‐napthol moiety but contains a stable tertiary amine in place of the hydrazide moiety found in Heatin, making it resistant to hydrolysis (Figure 4a). Starting from no. 202, an azide‐functionalized probe (Appendix S2, Figure 4a) was synthesized and subsequently linked to alkyne‐functionalized magnetic beads by means of the copper catalysed ‘click’ reaction (Figure 4b). The resulting Heatin‐coated beads were incubated with total‐protein extract of pooled 2–3‐day‐old seedlings. Bead elution was performed using the original Heatin molecule to enrich for Heatin‐specific targets and to remove unspecific proteins attached to the beads or probe molecule (Figure 4b). The resulting ‘Elute’ fraction and the ‘On‐bead’ remaining fraction were collected separately and analysed by liquid chromatography‐tandem mass spectrometry (LC‐MS/MS). After filtering of the MS results (Data S1), 212 protein groups were significantly enriched in the ‘Elute’ fraction compared with the ‘On‐bead’ fraction [at 5% false discovery rate (FDR), Figure 4c, Table S10].

Small molecules often bind multiple members of multiprotein families and hence chemical genetics approaches are particularly useful to overcome genetic redundancy (Dejonghe and Russinova, 2017; Tóth and van der Hoorn, 2010). We therefore searched for enriched molecular function GO terms among the 212 ‘Heatin‐eluted’ protein groups (Table S11, Figure S10a). This unbiased approach recovered phospholipase activator activity as the top overrepresented category. However, all recovered peptides that were assigned to ADP ribosylation factor proteins were shared among the six annotated phospholipase activator activity proteins in Arabidopsis, which are therefore not discriminative. Consequently, they were collectively assigned to a single protein group. Thus, it is undetermined if indeed several of these factors are present in the ‘Elute fraction’, as all members of a protein group were included in the GO term analysis. For this reason, we focused on the second highest fold enriched group: ‘indole‐3‐acetonitrile nitrilase/nitrile hydratase’ activity (88.02 times), consisting of the four members of the nitrilase protein family of which each member was uniquely identified and therefore assigned an independent protein group. Interestingly, of the four nitrilases, we recovered all three members of the *Brassicaceae‐*specific NIT1‐subfamily (NIT1, NIT2 and NIT3) as potential Heatin targets, but not the divergent NIT4 (Piotrowski, 2008; Vik *et al.*, 2018; Vorwerk *et al.*, 2001) (Figure 4c, Table S11). Indeed, of several tested crop species, only varieties belonging to the *Brassicaceae* exhibited Heatin‐induced hypocotyl elongation (Figure S10b, Appendix S3). Such hypocotyl elongation is a good proxy for growth and productivity of adult plants under high temperatures (Ibañez *et al.*, 2017).

We next tested whether the NIT1‐subfamily proteins are required for Heatin‐mediated hypocotyl elongation, by assaying sensitivity of Arabidopsis *nitrilase1‐subfamily* mutants to Heatin (Figure 4d; Figure S11). In the presence of Heatin, hypocotyls of *nit1‐3* (Normanly *et al.*, 1997) were significantly shorter than Col‐0 wild type in both control and high temperature (27°C) conditions (Figure 4d). A *35S::NIT1* overexpression line exhibited shorter hypocotyls under control conditions in the absence of Heatin, but this effect was overcome by Heatin, possibly due to increased sensitivity to the compound (Figure S11a). In the presence of Heatin, hypocotyl lengths of a *nit2* knock‐out mutant and two independent *NIT2 RNAi* lines (Lehmann *et al.*, 2017) were also reduced, whereas a *nit3* knock‐out mutant was not affected in its responsiveness (Figure 4d). Overall, our results indicate that NIT1 and NIT2 are required for full Heatin‐mediated hypocotyl elongation, whereas NIT3 has no apparent role. Interestingly, in the absence of Heatin hypocotyl elongation of *nit1‐3*, *nit2*, *NIT2 RNAi* knockdown and *NIT1* overexpression (Figure 4d; Figure S11a) lines all displayed reduced responsiveness to high temperature. Therefore, NIT1 and NIT2 can be considered contributors to seedling thermomorphogenesis. Heatin did not affect *NIT1‐subfamily* mRNA levels, indicating that Heatin operates at the protein level. Transcriptional regulation may be involved in NIT1‐subfamily contributions to thermomorphogenesis as mRNA levels responded to high temperatures (Figure S11b).

We next considered the possibility that NIT1‐subfamily members convert Heatin into HNA/HNC, similar to Sirtinol turnover into HNA. However, all tested *nit* mutants were sensitive to hypocotyl elongation induced by HNA and HNC application similar to wild‐type Col‐0 (Figure S11c). Moreover, Heatin additively stimulated hypocotyl elongation in the presence of supra‐optimal (Figure 1f) HNC (and to a lesser extend HNA) concentrations (Figure S11c). These results suggest that NIT1‐subfamily enzymes are likely not involved in Heatin turnover into HNA/HNC, which further underlines that Sirtinol/HNA and Heatin affect at least in part, different signalling pathways.

### Heatin affects auxin metabolism via NIT1‐subfamily proteins

**Figure 5:**
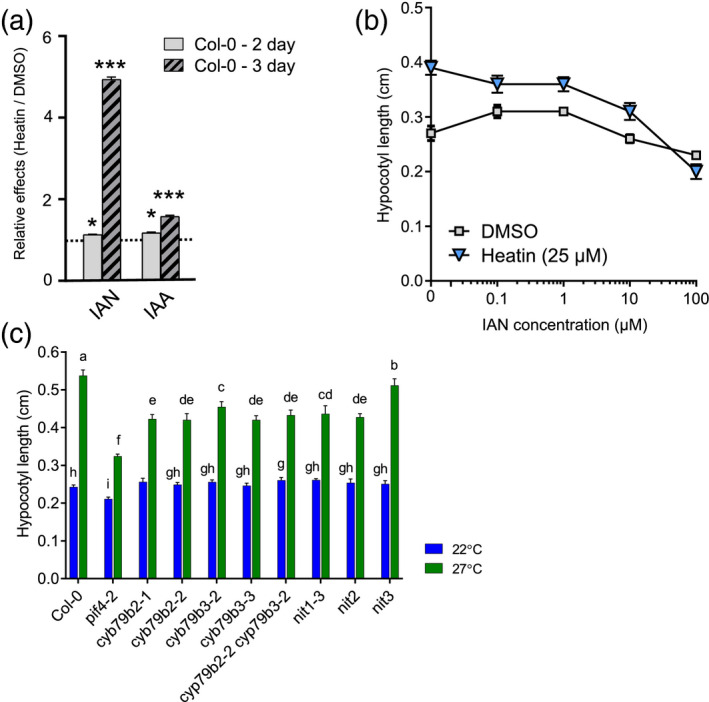
Heatin application results in indole‐3‐acetonitrile (IAN) and indole‐3‐acetic acid (IAA) accumulation required for hypocotyl elongation. (a) Relative Heatin effects on endogenous IAN and IAA levels. Two‐day‐old samples are shown as clear bars and 3‐day‐old samples are shown as striped bars. Horizontal dotted line indicates no difference between dimethyl sulphoxide (DMSO) and Heatin treatment. *N* = 4 replicates per treatment, each of 10 mg (fresh weight) seedlings. Asterisks indicate statistical significance, *P < 0.05, ***P < 0.001, determined by a Student’s *t*‐test. (b) Dose–response curves of hypocotyl lengths of 8‐day‐old Col‐0 wild‐type seedlings grown on medium containing various concentrations of IAN in the presence (blue triangles) or absence (mock; grey squares) of Heatin (25 µm). (c) Hypocotyl lengths of 8‐day‐old seedlings of Col‐0 wild‐type and various *cyp79b2* and *cypb79b3* and *nitrilase1‐subfamily* mutants, grown at 22°C (blue) and 27°C (green bars). Values are averages of (b) seven to eight independent repetitions of 15–68 seedlings, and (c) six to eight independent repetitions of 15–50 seedlings each. Error bars indicate SEM. Letters indicate significance groups (Tukey HSD *post‐hoc* test), where averages that do not share letters are significantly different from each other (*P* < 0.05). Note that these data provide independent confirmation for the observation that NIT1 and NIT2 contribute to thermomorphogenesis.

Previous reports indicated roles for NIT1‐subfamily members in defence and in auxin metabolism (Lehmann *et al.*, 2017; Piotrowski, 2008) and IAN is a known NIT1‐subfamily substrate that is converted by the nitrilases to IAA (Lehmann *et al.*, 2017). Given the involvement of auxin in Heatin‐induced hypocotyl elongation (Figure 3) on the one hand and the auxin signature observed among the genes affected by Heatin on the other hand (Tables S6–S9), we enquired whether Heatin modulates hypocotyl elongation by interfering with IAA metabolism. To this end, *in vivo* levels of IAN and IAA were quantified (Pěnčík *et al.*, 2018) in the presence and absence of Heatin (Figure 5a; Figure S13a,b). A striking significant increase in IAN abundance was detected in 2‐day‐old, but particularly 3‐day‐old seedlings. This *in vivo* IAN substrate accumulation prompted NIT1‐subfamily *in vitro* activity assays, using purified recombinant nitrilases (Piotrowski *et al.*, 2001) and different substrates (Figure 4e,f; Figure S12b–d). Results obtained showed that Heatin inhibits nitrilase enzyme activity as turnover of the substrate 3‐phenyl‐propionitrile (3‐PPN) (Vorwerk *et al.*, 2001) was reduced in the presence of Heatin (Figure 4e) in a dose–response manner, with an estimated half‐maximal inhibitory concentration (IC_50_) of 20.7 µm for NIT1 (Figure S12c,d). Turnover of 6‐heptenenitrile by recombinant NIT2 was accordingly reduced by Heatin (Figure 4f), showing that Heatin inhibition of NIT1‐subfamily enzyme activity is not substrate specific.

In the presence of Heatin the *in vivo* level of the nitrilase substrate IAN is increased but, remarkably, also *in vivo* levels of bioactive IAA significantly increased, in both 2‐ and 3‐day‐old seedlings (Figure 5a; Figure S13a,b). Probably, this rise in IAA abundance contributes to Heatin‐induced hypocotyl elongation, implying that observed Heatin‐imposed inhibition of NIT1‐subfamily enzyme may not be directly causal for hypocotyl elongation. Nitrilases are involved in IAA production and may modulate the response to Heatin. We tested the necessity of the IAN/IAA biosynthesis pathway for hypocotyl elongation at warm temperatures. A dose–response assay confirmed that IAN could stimulate elongation up to a concentration of 1 µm, whereas higher concentrations became suppressive (Figure 5b). However, in the presence of Heatin, IAN suppressed hypocotyl elongation already at a 10‐fold lower concentration (Figure 5b). In addition, we tested mutants in the P450 cytochromes CYP79B2 and CYP79B3. These enzymes convert TRP to indole‐3‐acetaldoxime, the precursor of IAN. *Cyp79b* mutants are nearly IAN‐depleted and display reduced hypocotyl elongation in response to high temperatures (Sugawara *et al.*, 2009; Zhao, 2012), which we confirmed in our experimental conditions (Figure 5c). Responsiveness to Heatin application was significantly reduced in *cyp79b* mutants as compared with the Col‐0 wild type (Figure S14a,b). This suggests that Heatin and IAN are associated with the IAA pathway, triggering hypocotyl elongation, although the exact mechanism of Heatin action and its contribution to hypocotyl elongation remains unclear.

## DISCUSSION

**Figure 6:**
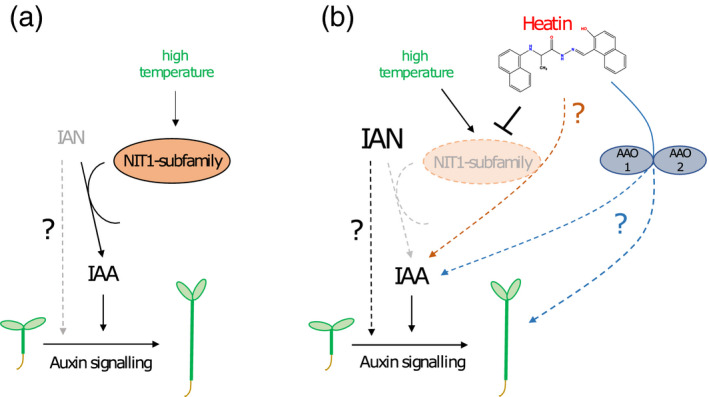
Proposed model of the role of the NIT1 family of nitrilases in hypocotyl elongation (thermomorphogenesis) in the absence and presence of the chemical compound Heatin. (a) In absence of the chemical compound Heatin. Hypocotyl elongation in high temperature conditions (27°C; green letters) requires the NIT1‐subfamily of nitrilase enzymes (orange oval). Members of the NIT1‐subfamily convert the auxin precursor indole‐3‐acetonitrile (IAN) into bioactive auxin indole‐3‐acetic acid (IAA). Hence, in high temperature conditions IAN substrate levels are relatively low (grey letters) and IAA product levels are relatively high (black letters). A possible direct effect of IAN on hypocotyl elongation (bypassing IAA) cannot be excluded and is here indicated as a grey‐dotted arrow and black question mark. How high temperature interacts with NIT1‐subfamily enzymes is unknown, but IAN substrate conversion by NIT2 is promoted by higher temperatures. Furthermore, NIT2 displays an atypical temperature sensitivity profile (Vorwerk *et al.*, 2001). Eventually, auxin perception and signalling are required for hypocotyl elongation. (b) In presence of the chemical compound Heatin. In this study, we report on the identification of the chemical compound Heatin (red letters and chemical structure). We demonstrate that NIT1 and NIT2 are required for Heatin‐mediated hypocotyl elongation and propose that Heatin can directly interact with NIT1‐subfamily members to inhibit their activity (orange oval with dashed outline and grey letters). As a result, IAN substrate levels are high in the presence of Heatin (bold black letters). Unanticipated, also a significant rise in the NIT1‐subfamily enzyme product, bioactive IAA, was observed because of Heatin application, which occurs via an unknown mechanism (orange dashed line and question mark). Although unanticipated, a similar finding has been reported before (Piotrowski *et al.*, 2001). This increase in IAA would likely contribute to observed Heatin‐induced hypocotyl elongation. However, as indicated above, we cannot exclude IAN to trigger hypocotyl elongation (bypassing IAA; dotted arrow). Nevertheless, it appears that the main effect of Heatin on hypocotyl elongation is probably not depending on the nitrilases, although nitrilases may indirectly affect the response to Heatin because of their role in the production of IAA. A possible alternative pathway for Heatin action may involve aldehyde oxidase 1 (AAO1) and AAO2 enzymes, as these redundantly contribute to Heatin‐mediated hypocotyl elongation. As AAO activity was proposed to be linked to IAA biosynthesis (Böttcher *et al.*, 2014; Seo *et al.*, 1998) it is possible that this AAO1/AAO2 route contributes to observed increased IAA levels in the presence of Heatin. AAO1/AAO2 might interfere with elongation growth via a yet unknown mechanism (blue dashed lines and question mark).

Temperature is a pervasive stimulus that affects all molecular processes in plants. Therefore, investigations on temperature signalling networks are prone to thermodynamic and other unspecific effects (Vu *et al.*, 2019). A further complication is the prevalence of genetic redundancy (Cutler and McCourt, 2005). To overcome these hurdles, we adopted a forward chemical genetics strategy (Dejonghe and Russinova, 2017) and identified the small molecule Heatin as an inducer of elongation growth (Figure 6). In a previous chemical genetics screen, the structurally related compound piperidine (no. 401) was also identified as inducer of hypocotyl elongation (Savaldi‐Goldstein *et al.*, 2008), indicating that the findings here reported are not restricted to our experimental conditions. In general, Heatin effects on hypocotyl growth are more pronounced at control temperatures than in high temperatures. A likely explanation for this is that the total elongation capacity is already partly saturated in the elongated hypocotyls at warm temperatures. This may limit the effective window of the compound Heatin.

We show that Heatin application results in *in vivo* accumulation of bioactive IAA and its precursors IAN (Figure 6b). Heatin effects are partly distinct from those of the synthetic auxinic compound picloram and might diverge at the level of AFB3, because mutants in this auxin receptor component are hypersensitive to Heatin, whereas normal picloram sensitivity is retained. Such a specific role for AFB3 would not be unprecedented. Specific roles for each auxin receptor are suggested and each receptor has different affinities for different auxinic compounds (Calderón Villalobos *et al.*, 2012; Parry *et al.*, 2009; Shimizu‐Mitao and Kakimoto, 2014). Interestingly, *afb3* single mutants were reported to be hypersensitive to auxin‐induced hypocotyl elongation (Chapman *et al.*, 2012) and to salt stress (Garrido‐Vargas *et al.*, 2020). Moreover, AFB3 was proposed to have a unique role in the responses of roots to nitrate (Vidal *et al.*, 2010). Although the exact mechanism remains unclear, it is hypothesized that this hypersensitivity is due to increased activity of other TIR1/AFB family members. This is in line with our observation that the *tir1* and *afb5* mutant are partially resistant to Heatin, while the *axr1‐3* mutant, which is disturbed in SCF^TIR1/AFB^ auxin receptor complex formation (del Pozo *et al.*, 2002; del Pozo and Estelle, 1999), is insensitive.

Alternatively, Heatin’s bioavailability could be restricted to the hypocotyl. Again, this would not be unprecedented, as several auxin analogues are effectively transported and metabolized *in situ* to release active auxins (Savaldi‐Goldstein *et al.*, 2008). Furthermore, removal of the hydroxyl group from 1‐aminomethyl‐2‐naphthol causes loss of hypocotyl specificity and removal of non‐core parts of the Heatin molecule reduces bioactivity. Heatin accumulation in the hypocotyl is indirectly supported by the observation that 25 µm Heatin is not sufficient for full nitrilase inhibition *in vitro* (Figure 4a; Figure S12c,d), whereas Heatin effects on hypocotyl elongation are clearly induced at a concentration of 5 µm and higher (Figure 2a). It is also possible that inhibition of nitrilases in the physiological context is more effective than in the *in vitro* test buffer. Alternatively, if Heatin directly competes with nitrilase substrate binding, the relatively high substrate levels in the *in vitro* experiment may have outcompeted Heatin binding, thereby dampening the inhibitive effect. The latter would implicate that NIT1‐subfamily proteins are direct molecular targets of the Heatin compound. Circumstantial evidence suggests this to be the case. First, in our chemical proteomics approach we uncovered the NIT1‐subfamily proteins with a Heatin‐derived probe as bait. Secondly, NIT1 and NIT2 are required for full Heatin‐mediated hypocotyl elongation, and thirdly, NIT1‐subfamily enzyme activity is reduced in the presence of Heatin. However, dedicated biochemical, and biophysical experiments are needed to confirm the direct molecular interaction between the ligand Heatin and the proposed NIT1‐subfamily targets and to establish how Heatin inhibits nitrilases (e.g. competitive or allosteric).

Nitrilases catalyse the hydrolysis of nitrile into the corresponding carboxylic acid and ammonia and are found throughout the plant kingdom, where they are mainly involved in cyanide detoxification and defence against pathogens and herbivory (Piotrowski, 2008; Vik *et al.*, 2018; Vorwerk *et al.*, 2001). In *Brassicaceae*, nitrilases can be divided into two subfamilies, i.e. NIT4 and the NIT1‐subfamily (NIT1, NIT2 and NIT3). NIT4 has a well understood role in cyanide detoxification (Piotrowski, 2008) and shows high substrate specificity. Nitrilases of the NIT1‐subfamily have among others a role in auxin biosynthesis (Lehmann *et al.*, 2017; Vik *et al.*, 2018) in a *Brassicaceae‐*specific pathway parallel to the main auxin biosynthesis route (Zhao, 2012). In the NIT1‐subfamily–dependent pathway, l‐tryptophan is metabolized into indole‐3‐acetaldoxime by the cytochrome P450 CYP79B2 (Lehmann *et al.*, 2017; Tóth and van der Hoorn, 2010) and subsequently converted into IAN by CYP71A1 and then hydrolysed by nitrilases into bioactive IAA (Lehmann *et al.*, 2017; Tóth and van der Hoorn, 2010).

Heatin treatment resulted in *in vivo* accumulation of its substrate IAN, in line with the observation that nitrilase activity is suppressed by Heatin *in vitro*. However, we also observed a significant increase in bioactive IAA levels. Such an apparent contradictory connection between NIT1, and IAA and IAN levels is not unprecedented, as it has been reported that *NIT1* overexpression resulted in reduced total IAA and IAN levels (Piotrowski *et al.*, 2001). However, the underlying mechanism is not understood. These findings suggest that the main effect of Heatin on hypocotyl elongation may not be through Heatin affecting the nitrilases, but that the effect of Heatin on hypocotyl elongation is due to increased IAA levels. However, the possibility remains that IAN directly stimulates elongation growth in a NIT1‐ and NIT2‐dependent manner (Figure 6), or that nitrilases indirectly affect the response to Heatin because of their role in IAA biosynthesis.

Previous modelling studies have suggested that IAN might possibly bind to the TIR1 auxin receptor directly to form stable complexes with AUX/IAA proteins (Katz *et al.*, 2015; Vik *et al.*, 2018). This suggestion should be taken with great caution, as IAN did not facilitate TIR‐AUX/IAA7 interaction in a yeast two‐hybrid assay, unlike IAA, and the modelled binding of IAN to TIR1 does not occupy the same region on chain B, close to Ala299, but occurs on chain C (Katz *et al.*, 2015; Vik *et al.*, 2018).

We considered whether Heatin could be metabolized *in situ* to release an auxinic compound affecting auxin metabolism, analogous to Sirtinol’s action relying on hydrolysis of its hydrazine bond to release HNA. Aldehyde oxidase proteins could then metabolize HNA into HNC (Dai *et al.*, 2005; Zhao *et al.*, 2003). However, several of our observations indicate that Heatin is not directly metabolized into HNA/HNC. First, Heatin acts in an additive way to supra‐optimal HNA concentrations (Figure S11a). Second, Heatin does not require ARF7 and ARF19 to exert its effect (Figure 3b; Figure S6c). Third, Heatin did not activate the *DR5* auxin reporter promoter (Figure S6d). Fourth, compound no. 202 used for our chemical proteomics experiment is less prone to hydrolysis and has a similar activity as Heatin, suggesting that Heatin metabolism *in situ* is probably not a prerequisite for its bioactivity. Finally, from a chemical perspective, compound no. 202 cannot be hydrolysed via the same mechanism as Heatin; even if hydrolysed, the magnetic beads used in our proteomics approach would separate from the active moiety of the molecule (Figure 4c), which would have precluded NIT1‐subfamily recovery. Nevertheless, we observed resistance of *sir1* to Sirtinol and Heatin, suggesting that aldehyde oxidation capacity is required for Heatin’s effect (Dai *et al.*, 2005). Heatin resistance of the *aao1‐1 aao2‐1* double mutant supports this notion. It is tempting to speculate that Heatin may in part affect elongation growth by modulating auxin metabolite levels by interfering with aldehyde oxidation capacity, as AAO1 has been implicated in IAA biosynthesis (Böttcher *et al.*, 2014; Seo *et al.*, 1998) (Figure 6b). However, the contribution of AAO proteins to auxin biosynthesis *in planta* is still under debate (Mashiguchi *et al.*, 2011; Seo *et al.*, 1998; Seo *et al.*, 2004). In conclusion, we propose that Heatin has a complex mode of action, which may involve inhibiting NIT1‐subfamily activity and affecting an AAO‐dependent process (Figure 6b).

We also observed that *NIT1* and *NIT2* contribute to high temperature‐induced hypocotyl elongation in the absence of Heatin, demonstrating that these proteins have a role in seedling thermomorphogenesis (Figure 6a). Interestingly, high temperature affects NIT2 enzymatic activity, analogous to Heatin‐mediated inhibition of NIT2 activity, as the thermal optimum for enzymatic IAN substrate conversion of NIT2 is at the relatively low temperature of 12–15°C and decreases rapidly at higher temperatures (Vorwerk *et al.*, 2001). This atypical temperature sensitivity profile is striking, as it is not observed for NIT1 or NIT3, or for other NIT2 substrates, and may play a critical role in modulating IAA/IAN levels at warm temperatures and consequently thermomorphogenesis.

In conclusion (Figure 6), our work assigns a role to the NITRILASE1‐subfamily in mediating thermomorphogenesis and identifies Heatin as a chemical entity for studies on auxin biology. Given the large number (no. 212) of protein groups that were significantly enriched in the ‘Elute’ fraction in our chemical proteomics approach, it is possible that Heatin affects (elongation) growth via one or several of the other interacting proteins. We cannot exclude that Heatin may affect other processes in the plant that possibly contribute to elongation growth than merely affecting auxin biosynthesis, either by targeting other candidate proteins than the NIT1‐subfamily, or via the contribution of NIT1‐subfamily enzymes to other metabolic pathways (Lehmann *et al.*, 2017; Vik *et al.*, 2018). The relevance and applicability of the Heatin–nitrilase connection in modulating cell expansion, plant growth and acclimation to high temperature should therefore be further validated in future experiments. Nevertheless, we propose that Heatin and its functional analogues can be of use in *Brassicaceae* crop systems as agrochemicals to facilitate optimal growth under suboptimal temperature conditions. No detrimental side‐effects of Heatin applications were observed in our experimental set‐ups, but toxicological assessments are required before Heatin can be considered for use in agricultural or horticultural practice.

## Experimental procedures

### Plant materials and growth conditions

Arabidopsis seeds were obtained from the Nottingham Arabidopsis stock centre (www.arabidopsis.info) or were kind gifts of colleagues. The following lines were used: Col‐0, L*er* and C24 wild types, *pif4‐2* (Leivar *et al.*, 2008), *sir1* (Zhao *et al.*, 2003), *sir3‐1* (Dai *et al.*, 2005), *sir/N22A* (Teschner *et al.*, 2010), *atCand1‐2* (homozygous genotyped SALK_099479) (Cheng *et al.*, 2004), *tir1‐1* (Ruegger *et al.*, 1998), *afb1‐3* (Savaldi‐Goldstein *et al.*, 2008), *afb2‐3* (Savaldi‐Goldstein *et al.*, 2008), *afb3‐4* (Parry *et al.*, 2009), *afb5‐5* (Salk_110643) (Prigge *et al.*, 2016), *tir1‐1 afb2‐3* (Savaldi‐Goldstein *et al.*, 2008), *tir1‐1 afb5‐5* (Gleason *et al.*, 2011), *axr1‐3* (Estelle and Somerville, 1987), *aao3‐1* (Seo *et al.*, 2004), *aao3‐2* (González‐Guzmán *et al.*, 2004), *aao3‐4* (homozygous genotyped SALK_072361) (Seo *et al.*, 2004), *aao4‐1* (homozygous genotyped SALK_047520) (Ibdah *et al.*, 2009), *aao4‐2* (homozygous genotyped SALK_057531) (Ibdah *et al.*, 2009), *eDR5:LUC* (Covington and Harmer, 2007), *gpa1‐4* (Jones *et al.*, 2003), *agb1‐2* (Ullah *et al.*, 2003), *gpa1‐4 agb1‐2* (Ullah *et al.*, 2003), *gcr1‐2* (Chen *et al.*, 2004), *gcr2‐4* (Gao *et al.*, 2007), *agg1* (Trusov *et al.*, 2007), *agg1‐1 agg2‐1* (Trusov *et al.*, 2007), *rgs1‐1* (Chen *et al.*, 2003), *gcr triple* (Guo *et al.*, 2008), *arf7‐1* (Okushima *et al.*, 2005), *arf7‐1 arf19‐1* (Okushima *et al.*, 2005), *nit1‐3* (Normanly *et al.*, 1997), *nit2* (SM_3_24059) (Trompetter, 2010), *nit3* (GK_04E09) (Trompetter, 2010), *NIT2‐RNAi* line nos 8–9 and no. 26‐6 (Lehmann *et al.*, 2017), *NIT1OE* (Lehmann *et al.*, 2017), *cyp79b2‐1* (Sugawara *et al.*, 2009), *cyp79b2‐2* (Sugawara *et al.*, 2009), *cyp79b3‐2* (Sugawara *et al.*, 2009), *cyp79b3‐3* (Sugawara *et al.*, 2009) and *cyp79b2‐2 cyp79b3‐2* double (Sugawara *et al.*, 2009). The following genotyped homozygous T‐DNA insertional lines were generated (Alonso *et al.*, 2003): SALK_073700 (*aao1‐2*), SALK_011511 (*aao1‐1*), SALK_104895 (*aao2‐2*), GABI_379H03 (*aao2‐1*) and SAIL_78_H09 (*aao3‐5*). The *aao1 aao2* double mutant was obtained by crossing SALK_011511 and GABI_379H03 lines. The selection of homozygous plants was done by checking for the insertion by polymerase chain reaction (Table S12). Reverse transcriptase–polymerase chain reaction confirmed the absence of full‐length transcripts (Figure S5c). Seeds of crop varieties were commercial batches of F1 hybrids supplied by Bejo Zaden BV (Warmenhuizen, the Netherlands).

Plant materials were grown as in van der Woude *et al.* (2019) on sterile 0.8% plant agar (Duchefa P1001), 1× Murashige–Skoog medium (including MES Buffer and vitamins; Duchefa M0255) without sucrose in Petri dishes, unless stated otherwise. Seeds were surface sterilized by a solution of 0.8% commercial bleach (Glorix) in ethanol for 10 min, followed by twice washing with ethanol for 10 min, or by chlorine gas for 3 h. After sowing, seeds were stratified for 2–3 days (Arabidopsis) or 1 night (crops) at 4°C in darkness. The Petri dishes containing the plants were subsequently grown (van der Woude *et al.*, 2019) under 100–125 µmol m^−2^ sec^−1^ PAR, short day photoperiod conditions (8 h light/16 h darkness) at 70% relative humidity in climate‐controlled Microclima 1000 growth cabinets (Snijders labs, Tilburg, the Netherlands) at either 22°C (control) or 27°C (high temperature), unless stated otherwise.

### Compound library screening and hit confirmation

The small aromatic compound library ‘Laboratories of Chemical Biology Umeå (LCBU) Screening Set’ was used for initial screening. This set contains mainly aromatic drug‐like molecules covering a wide range of chemical space and was purchased from Chembridge Corp. (San Diego, CA, USA) 8000 compounds out of the total 17 500 were screened. In parallel, a library of 360 compounds previously found to be active in plants (Drakakaki *et al.*, 2011) was tested. One µl of each compound was automatically pipetted (Biomek NX, Beckman Coulter pipetting robot) from the 5 mm stock solution to a well in a 24‐well plate. One times 600 µl Murashige–Skoog plant agar medium was added to each well manually. The final concentration of each compound in the wells was 8.3 µm. NPA (Duchefa, Amsterdam, the Netherlands) and picloram (Sigma‐Aldrich, Zwijndrecht, the Netherlands) were both dissolved in dimethyl sulfoxide (DMSO) and manually added to each 24‐well plate to Col‐0 wild type (negative control) and *pif4‐2* (positive control) respectively (4.18 µm) for internal standardization. DMSO 0.1% lacking an active compound was used as the mock solvent control.

Seeds were surface‐sterilized using a 0.1% Tween‐20, 70% ethanol solution for 2 min and subsequently washed with 95% ethanol. Six seeds were manually added to each well in a horizontal line and dispersed using a toothpick. The plates with seeds were stratified in the dark at 4°C for 3 days to synchronize germination. Subsequently, plates were pre‐germinated at 22°C, 100 µmol m^−2^ sec^−1^ long day (16 h photoperiod) conditions for 24 h. The plates were then moved to a growth cabinet (Percival Scientific Inc., Perry, IA, USA) for 8 days, set at 28°C, 75 µmol m^−2^ sec^−1^ in short day conditions (8‐h photoperiod), after which the plates were scanned using a flatbed scanner. Hypocotyl lengths were scored visually. As auxins are effective inducers of high temperature‐induced hypocotyl elongation (Franklin *et al.*, 2011; Gray *et al.*, 1998), we intended to exclude canonical auxinic compounds. All compounds that resulted in a display of the typical auxin‐related phenotypes such as small, inward curved leaves, reduced root growth (Oh *et al.*, 2014; Sorin *et al.*, 2005) and agravitropic growth, in addition to hypocotyl elongation, were excluded from further analyses. To confirm the initial hits and reduce false positive hits, a validation repetition was performed with the initial hit compounds. Of the 36 compounds with reproducible effects hypocotyl lengths were quantified at 22 and 27°C using fresh powder derived from the Chembridge vendor, to exclude effects of possible compound decay or contaminations. Two compounds were not available for follow‐up studies.

### Pharmacological compound applications

Heatin used for candidate hit confirmation was commercially obtained from Chembridge (no. 5713980). Other experiments were performed with *in‐house* synthesized Heatin (Appendix S4). Names, sources and vendor IDs of all chemicals used in this study are in Table S3; Table S4. All compounds were dissolved in DMSO (D4540; Sigma‐Aldrich) and applied to the medium in a final DMSO concentration of 0.1% (v/v). DMSO lacking added compounds was used as solvent (mock) control. Chemical properties of compounds were retrieved from the vendor’s information or public chemical databases.

### Phenotyping

Petri dishes containing seedlings for hypocotyl elongation quantification and root length measurements were scanned using a flatbed scanner and lengths were measured using ImageJ software (https://imagej.nih.gov/ij/) as in van der Woude *et al.* (2019).

Plants for vegetative rosette trait measurements were grown on sterile 0.8% plant agar as described above, in ‘Sterivent High Containers’ (S1686; Duchefa). Six plants per container were grown in several batches until the first plants started bolting. Then, photos were taken from the side for leaf angle measurement and subsequently plants were flattened and photographed from the top. Plants were weighed, and the rosette surface was determined using a LI‐3100 Surface Area Meter (LI‐COR). The petiole and leaf blade length per plant was measured by ImageJ and defined as the average of the lengths of the third to sixth youngest leaves. Hyponastic growth was measured by ImageJ and defined as the average of the angle of two opposing petioles per plant with a petiole length between 0.5 and 1 cm, relative to the horizontal.

Seedling agravitropy was scored by qualification of the growth direction of hypocotyls relative to the direction of gravity. Hypocotyls that deviated more than 45° from the opposite of the direction of gravity, were considered agravitropic. Note that in the absence of a gravitropic response approximately 75% of the seedlings are considered agravitropic by this method.

Phenotypic data were analysed using anova followed by *post‐hoc* Tukey HSD tests using a script generated in R (www.r‐project.org), or when values relative to the control or wild type are shown, by a one‐sample *t*‐test.

### Luciferase assays

Assays were done as described in van der Woude *et al.* (2019). Protein extracts were made of approximately 25 mg freshly harvested seedlings by grinding with a micro‐pestle in 100 µl 1× passive lysis buffer (E1941; Promega, Leiden, the Netherlands) followed by 10‐min incubation at room temperature. Debris was pelleted by 5 min maximum speed (16 000 ***g***) centrifugation. Twenty microliters of supernatant was transferred to a 96‐well Lumitrac‐200 plate (82050‐726; VWR, Amsterdam, the Netherlands). Luciferase activity was assayed using a Glomax 96 microplate luminometer (E6521; Promega). The ‘Luciferase Assay System’ (E1500; Promega) was used with the ‘LUC Assay System with Injector’ protocol (2‐sec delay between injection and measurement, 10‐sec integration time). Subsequently, protein concentrations were determined of each sample using the Bradford method (Bradford reagent: Sigma‐Aldrich; B6916). Absorbance was measured using a Biotech synergy HT plate reader. A bovine serum albumin (A7906; Sigma‐Aldrich) standard curve in passive lysis buffer was used to calculate protein concentrations of each sample. Luciferase signals were corrected for background signal determined by assaying Col‐0 wild type, lacking Luciferase and normalized to the protein concentration of each sample.

### Time‐lapse hypocotyl elongation assays

Assays were done as described in van der Woude *et al.* (2019) with a custom digital time‐lapse camera system consisting of a Canon EOS 350D DSLR camera of which the standard internal IR and UV rejection filters were replaced by a 715‐nm long‐pass filter, allowing detection of wavelengths beyond 715 nm. The camera was placed in front of vertical‐positioned Murashige–Skoog agar plates containing the seedlings and photos were taken with 2‐h intervals for 8 days, using an Aputure AP‐R1C LCD Timer Remote controller. An LED spotlight (940 ± 50 nm; no. BL0106‐15‐28; Kingbright) was used to illuminate seedlings continuously, in addition to the growth cabinet lights. The emitted light did not interfere with plant development as no de‐etiolation of dark‐grown etiolated plants, nor germination of imbibed seeds was observed in otherwise continuous darkness (van der Woude *et al.*, 2019).

### Generation of plant material for approximately omics analyses

To generate samples for RNA‐seq and chemical proteomics, seedlings were grown on sterile 0.8% (RNA‐seq) or 1% (chemical proteomics) plant agar (P1001; Duchefa) with 1× Murashige–Skoog medium (including MES Buffer and vitamins, M0255; Duchefa) without sucrose, as in van der Woude *et al.* (2019). Surface‐sterilized seeds were sown and stratified for 2–3 days at 4°C in darkness and then transferred to the climate cabinet. For transcriptomics, plates were photographed from the top for hypocotyl length measurements at the start of the photoperiod of day 3 (2 day‐old seedlings, 48 h) and day 8 (7‐day‐old seedlings; 168 h) and thereafter harvested into 1.5 ml reaction tubes and snap‐frozen in liquid N_2_. Each sample contained 100–200 seedlings. For the RNA‐seq samples three samples (50–100 seedlings) harvested and grown independently in time were combined (van der Woude *et al.*, 2019). Effectiveness of the treatments was confirmed by measuring the hypocotyl lengths of the replicates using ImageJ (Figure S9a). For chemical proteomics, 2‐ (48 h), 2.5‐ (56 h) and 3‐day‐old (72 h) seedlings were harvested and snap‐frozen in liquid N_2_ similarly as for the transcriptomics experiment.

### RNA‐seq

RNA‐seq was done as described in van der Woude *et al.* (2019) Plant tissues were ground by adding glass beads to the reaction tubes using a TissueLyser II (60‐sec runtime, 30 Hz; Qiagen, Venlo, the Netherlands). RNA was isolated using the Sigma Spectrum Plant Total RNA isolation kit and gDNA was removed by on‐column DNAse treatment (Sigma‐Aldrich). RNA integrity and concentration were checked using RNA 6000 Nano Chips on a Bioanalyser (2100; Agilent Technologies, Amstelveen, the Netherlands). For RNA‐seq library preparation, in total, three samples were prepared for each treatment and time‐point, by combing isolated RNA of three individually harvested batches per sample, each containing multiple seedlings. Illumina TruSeq RNA Library preparation and Illumina HiSeq2500 (high‐throughput) single‐end 50 bp sequencing was outsourced to Macrogen, Korea. Quality control was performed in‐house on the raw‐sequencing reads before analysis using FastQC (www.bioinformatics.babraham.ac.uk/projects/fastqc). Subsequently the raw reads were aligned to the Arabidopsis genome (TAIR10) using tophat v2.0.131 with the parameter settings: ‘bowtie’ (Trapnell *et al.*, 2009), ‘no‐novel‐juncs’, ‘p 6’, ‘G’, ‘min‐intron‐length 40’ and ‘max‐intron‐length 2000’. Aligned reads were summarized over annotated gene models using HTSeq‐count (Anders *et al.*, 2015) v0.6.12 with settings: ‘‐stranded no’, ‘gene_id’. From the TAIR10 GTF file all ORFs of which the annotation starts with ‘CPuORF’ were manually removed previous summarization to avoid not counting all double annotated bZIP TF family members. Sample counts were depth‐adjusted and differential expression was determined using the DESeq package (Anders and Huber, 2010), with default settings. All statistics associated with testing for differential gene expression were performed with R (www.r‐project.org). GO‐term analyses were performed using the AgriGO online tool at: http://bioinfo.cau.edu.cn/agriGO/analysis.php using standard settings. RNA‐seq datasets are deposited at the National Center for Biotechnology information, Gene Expression Omnibus (https://www.ncbi.nlm.nih.gov/geo/) under accession number GSE130964.

### Probe synthesis and click chemistry

Materials and methods used to synthesize the azide‐functionalized probe can be found in Appendix S2. Magnetic Heatin‐coated beads were generated through a Cu(I)‐catalysed Huisgen azide‐alkyne 1,3‐dipolar cycloaddition reaction with the following substituents: 1 ml 10 mg ml^−1^ alkyne‐functionalized (24.8 nmol alkyne mg^–1^) magnetic beads (total 248 nmol alkyne groups; CLK‐1035‐1, Jena Bioscience, Jena, Germany), approximately 1 µmol azide‐functionalized compound no. 202 dissolved in 50 µl DMSO, 0.05 µmol CuSO_4_ dissolved in water, 0.07 µmol Tris[(1‐benzyl‐1H‐1,2,3‐triazol‐4‐yl)methyl]amine (TBTA) dissolved in DMSO and 0.2 µmol Tris(2‐carboxyethyl)phosphine hydrochloride (TCEP) dissolved in DMSO. The total reaction volume was 3 ml, constituting 1 ml bead suspension, 900 µl water containing the CuSO_4_, 900 µl tert‐butyl alcohol and 200 µl DMSO containing the other reaction components. TBTA and CuSO_4_ were mixed first to allow complex formation followed by TCEP and the probe solution and finally the bead suspension. The reaction was incubated overnight while stirring at room temperature. Beads were pelleted with a magnet and washed three times with a cycle of DMSO, a 50% tert‐butyl alcohol solution in water and water. This was followed by three final wash steps with water. Beads were resuspended in water, aliquoted and stored at 4°C until use in the pulldown experiments.

### Pulldown experiments

Protein extracts of four independent biological replicates were generated by grinding approximately 420 2‐, 2.5‐ and 3‐day‐old pooled seedlings per replicate, in 200 μl extraction buffer [10 mm Tris/Cl pH 7.5, 150 mm NaCl, 0.3% NP‐40, Protease Inhibitor Cocktail (11836170001 Roche, Welwyn Garden City, UK), 1 mm 1,4‐dithiothreitol (DTT)]. Supernatant was collected after centrifugation (16 000 ***g***, 10 min at 4°C). 100 µl of beads was equilibrated using extraction buffer by washing three times and subsequently added to the protein extracts and incubated for 1 h at 4°C tumbling end‐over‐end.

Beads were washed 10× using extraction buffer. Sodium dodecyl sulphate–polyacrylamide gel electrophoresis protein gel analysis confirmed the presence of proteins attached to the beads (Figure S12a). The beads were transferred to new tubes after the first and the last washing step and thereafter resuspended in 200 µl extraction buffer. 0.2 µl 25 mm Heatin in DMSO was added to elute Heatin binding proteins. The elution was incubated for 30 min at 4°C and separated from the beads. Beads were resuspended in extraction buffer.

Elutes were reduced, alkylated and digested *in solute* by adding 10 μl 1 m DTT and incubated for 1 h at room temperature, then 10 μl 1 m iodoacetamide was added. Samples were incubated in darkness at room temperature for 1 h. Cysteine 2.5 μl 1 m was added to capture free iodoacetamide. Proteins were precipitated using a methanol/chloroform extraction and resuspended in 50 μl 6 m urea in Tris/Cl pH 8.0. 1.9 μl Trypsin/Lys‐C mix (Promega, Southampton, UK) was added and incubated for 3 h at 37°C. Two hundred and fifty microlitres of Tris buffer was added to bring the urea concentration to <1 m and then incubated overnight. The digestion was stopped by adding 1.5 μl trifluoroacetic acid (0.5%). Samples were centrifuged, and supernatants were purified by Sep‐Pak C18 (WAT020515; Waters, Milford, MA, USA) and analysed by LC‐MS/MS (see below).

*On‐bead* samples were reduced, alkylated and digested by washing twice with 50 mm Tris buffer pH 8.0 and subsequently resuspended in 50 μl of the same buffer. This was followed by adding 2.5 μl 1 m DTT and incubation for 1 h at room temperature, then by adding 2.5 μl 1 m iodoacetamide and incubation in darkness at room temperature for 1 h. Cysteine2.5 μl 1 m was added to capture free iodoacetamide. Urea at 200 μl 8 m was added and directly after 1.9 μl Trypsin/LysC, followed by incubation for 3 h at 37°C. Tris buffer at 1 ml was added to reduce the urea concentration to <1 m. Samples were incubated overnight. Digestion was stopped by adding 6 μl trifluoroacetic acid (0.5%). Beads were separated from supernatant, which was saved and purified by Sep‐Pak C18 (WAT020515; Waters) and analysed by LC‐MS/MS (see below). All steps in the pulldown experiment were done using Protein LoBind tubes (catalog no. 0030108116; Eppendorf).

### Targeted proteomics and protein identification

LC‐MS/MS analysis was performed on all four biological replicates of the Heatin‐eluted and the ‘On bead’ fractions separately, on an Orbitrap Elite (Thermo) (Michalski *et al.*, 2012) coupled to an EASY‐nLC 1000 LC system (Thermo) operated in the one‐column mode. The analytical column was a fused silica capillary (75 µm × 30 cm) with an integrated PicoFrit emitter (New Objective, Littleton, MA, USA) packed in‐house with Reprosil‐Pur 120 C18‐AQ 1.9 µm resin. The analytical column was encased by a column oven (Sonation) and attached to a nanospray flex ion source (Thermo). The column oven temperature was adjusted to 45°C during data acquisition. The LC was equipped with two mobile phases: solvent A (0.1% formic acid, fatty acids, in water) and solvent B (0.1% fatty acids in acetonitrile) of ultra‐performance LC grade (Sigma). Peptides were directly loaded on to the analytical column. Peptides were subsequently separated on the analytical column by running a 140‐min gradient of solvent A and solvent B (start with 7% B; gradient 7–35% B for 120 min; gradient 35–80% B for 10 min and 80% B for 10 min) at a flow rate of 300 nl min^–1^. The mass spectrometer was operated using xcalibur software (version 2.2 SP1.48). The mass spectrometer was set in the positive ion mode. Precursor ion scanning was performed in the Orbitrap analyser (Fourier transform MS) in the scan range of 300–1800 *m*/*z* and at a resolution of 60 000 with the internal lock mass option turned on (lock mass was 445.120025 *m*/*z*, polysiloxane) (Olsen *et al.*, 2005). Product ion spectra were recorded in a data‐dependent fashion in the ion trap MS in a variable scan range and at a rapid scan rate. The ionization potential (spray voltage) was set to 1.8 kV. Peptides were analysed using a repeating cycle consisting of a full precursor ion scan (3.0 × 10^6^ ions or 50 ms) followed by 15 product ion scans (1.0 × 10^4^ ions or 50 ms) where peptides are isolated based on their intensity in the full survey scan (threshold of 500 counts) for MS/MS generation that permits peptide sequencing and identification. Collision‐induced dissociation energy was set to 35% for the generation of MS/MS spectra. During MS/MS data acquisition, dynamic ion exclusion was set to 120 sec with a maximum list of excluded ions consisting of 500 members and a repeat count of 1. Ion injection time prediction, preview mode for the Fourier transform MS, monoisotopic precursor selection and charge state screening were enabled. Only charge states >1 were considered for fragmentation.

RAW spectra were submitted to an Andromeda (Cox *et al.*, 2011) search in MaxQuant (version 1.6.2.6) using the default settings (84). Label‐free quantification and match‐between‐runs was activated (Cox *et al.*, 2014). MS/MS spectra data were searched against the TAIR10 *A. thaliana* representative gene model FASTA file as reference proteome (TAIR10_pep_20110103_representative_gene_model_updated.fasport.org; 27416 entries). All searches included a contaminants database (as implemented in MaxQuant, 246 sequences). This contaminants database contains known MS (mass spectrometry) contaminants (i.e. human proteins picked up during sample preparation) and was included to estimate the level of contamination. Andromeda searches allowed oxidation of methionine residues (16 Da) and acetylation of the protein N‐terminus (42 Da) as dynamic modifications and the static modification of cysteine (57 Da, alkylation with iodoacetamide). Digestion mode was set to ‘specific’, enzyme specificity was set to ‘Trypsin/P’ with two missed cleavages allowed, the instrument type in Andromeda searches was set to Orbitrap and the precursor mass tolerance to ±20 ppm (first search) and ±4.5 ppm (main search). The MS/MS match tolerance was set to ±0.5 Da and the peptide spectrum match FDR and the protein FDR to 0.01 (based on the target‐decoy approach and decoy mode ‘revert’). Minimum peptide length was seven amino acids. Minimum score for unmodified peptides was set to 0. For protein quantification modified peptides (minimum score 40) and unique and razor peptides were allowed.

Further analysis, filtering and annotation of the results was done in perseus v1.6.2.1 (Tyanova *et al.*, 2016). Processed data can be found in Data S1. Detected protein groups were filtered to remove potential contaminants, reverse hits and hits only identified by a modification site. Only protein groups with at least three MS/MS counts over all runs were considered for further analysis. For quantification, related biological replicates were combined into categorical groups to allow comparison of the ‘Heatin‐eluted’ fraction with the ‘On‐bead’ fraction and only those proteins that were found in at least one categorical in a minimum of three of four biological replicates were investigated. Before quantification, missing values were imputed from a normal distribution with default settings. Comparison of protein group quantities (relative quantification) between different MS (mass spectrometry) runs was solely based on the log2‐transformed label‐free quantification (LFQ) intensities as calculated by MaxQuant (MaxLFQ algorithm). Briefly, label‐free protein quantification was switched on and unique and razor peptides were considered for quantification with a minimum ratio count of 2. Retention times were recalibrated based on the built‐in non‐linear time‐rescaling algorithm. MS/MS identifications were transferred between LC‐MS/MS runs with the ‘Match between runs’ option in which the maximal match time window was set to 0.7 min and the alignment time window set to 20 min. The quantification was based on the ‘value at maximum’ of the extracted ion current. At least two quantitation events were required for a quantifiable protein (Cox *et al.*, 2014).

Visualization of relative protein quantification was done by generating a volcano plot where statistical significance was determined by a two‐sided Student’s *t*‐test (Figure 4c; FDR: 0.05, S0: 0.01). Only significantly enriched protein groups (212 in total) were kept for further analysis. Next, a single entry row for multiple assigned ordered gene locus identifiers per protein group was created and gene descriptors were added (www.arabidopsis.org; Bulk data retrieval tool) (Table S10). Subsequently, a GO‐term enrichment analysis was done on the set of 212 enriched proteins in the ‘Elute fraction’ using the TAIR GO term tool (http://www.arabidopsis.org/tools/go_term_enrichment.jsp), which redirects the query to the Panther online repository (Panther14.1; GO Ontology database Released 2019‐07‐03; http://pantherdb.org/) and ‘molecular function’ was examined based on a Fisher’s exact test against the Arabidopsis proteome reference list (27581 entries; Bonferroni‐corrected *P* < 0.05) (Figure S10a, Table S11). MS proteomics data have been deposited to the ProteomeXchange Consortium via the PRIDE (Vizcaíno *et al.*, 2016) partner repository (https://www.ebi.ac.uk/pride/archive/) under identifier PXD015411.

### Enzymatic activity assays

Recombinant NIT1, NIT2 and NIT3 was purified from 1 L *Escherichia coli* culture as described before (Piotrowski *et al.*, 2001). The cell culture was induced by 0.3 mm (v/v) IPTG for 6 h and subsequently centrifuged. Pellets were resuspended in lysis buffer (50 mm sodium phosphate pH 8.0, 300 mm NaCl, 10 mm imidazole, 5 mm beta‐mercaptoethanol, 1 mg ml^−1^ lysozyme). After incubation, the suspension was sonicated using an ultrasound tip (Sonifier B‐17; Branson) in an ultrasonic ice bath. Debris was pelleted by centrifugation. Nitrilases were enriched by (NH_4_)_2_SO_4_ precipitation (40% saturation). Precipitate was collected by centrifugation and resuspended in 12 ml lysis buffer without lysozyme. This suspension was centrifuged again, and supernatant was saved as ‘enriched extract’ (Figure S12b). 6xHis‐tagged nitrilases were purified by loading the enriched extracts on to a Ni‐NTA affinity purification column. The flow‐through was saved for downstream purification analysis (‘Flow‐through’; Figure S12b), washed with lysis buffer with increased imidazole concentration (40 mm) and eluted with lysis buffer with higher imidazole concentration (250 mm). Nitrilase 2.5 ml containing fraction was saved and desalted using a PD‐10 column (Amersham Pharmacia Biotech, Amersham, UK). This resulted in highly purified nitrilase solution in 50 mm potassium phosphate, pH 8.0, 1 mm DTT (‘purified protein’; Figure S12b). The concentration of purified protein fractions was measured by the Bradford method, yielding 390 ng µl^−1^ NIT1, 640 ng µl^−1^ NIT2 and 870 ng µl^−1^ NIT3. Purified protein was aliquoted and flash‐frozen in liquid nitrogen and stored at −80°C until use.

Nitrilase activity assays were performed by measuring produced ammonia at different time points and Heatin concentrations by colorimetric Berthelot’s reaction (Van Slyke and Hiller, 1933), *in triplo* for DMSO mock samples and Heatin samples. Additional DMSO and Heatin samples with heat‐denatured nitrilase protein were included as negative controls to determine background signal. The reaction solution consisted of 50 mm potassium phosphate buffer, pH 8.0, 1 mm DTT and 2.5 mm 3‐PPN or 6‐heptenenitrile substrate unless otherwise stated, 5/10/100 µl purified nitrilase solution and 10 µl 1% DMSO in methanol with or without Heatin (25 µm final Heatin concentration). Water was added up to 1 ml. Reactions were performed at 37°C. The resulting product was analysed by measuring extinction at 640 nm at different time points.

The half maximal inhibitory concentration of Heatin for NIT1 activity (IC_50_) of 3‐PPN substrate turnover was estimated by interpolation of a linear regression model in Microsoft Excel (Figure S12d).

### IAN and IAA quantifications

Assays were done as described in van der Woude *et al.* (2019). Plates with seedlings were photographed from the top for hypocotyl length measurements at the start of the photoperiod of day 3 (2‐day‐old seedlings, 48 h) and day 8 (7‐day‐old seedlings; 168 h) before harvest in liquid N_2_.

Quantification of IAN and IAA metabolites were performed according to the method described by Pěnčík *et al.*, (2018). Samples (10 mg fresh weight) were homogenized and extracted in 1.0 ml of ice‐cold sodium phosphate buffer (50 mm, pH 7.0) containing 0.1% diethyldithiocarbamic acid sodium salt together with a cocktail of stable isotope‐labelled internal standards (5 pmol of [^13^C_6_]IAA and [^13^C_6_]IAN per sample added). Extracts were purified using the in‐tip microSPE based on the StageTips technology (Rappsilber *et al.*, 2003). Briefly, a volume of 250 μl of each plant extract was acidified to pH 2.7 with 0.1 m hydrochloric acid (approximately 100 μl). Combined multi‐StageTips (containing C18/SDB‐XC layers) were activated sequentially, with 50 μl each of acetone, methanol and water, by centrifugation. After application of aliquots of the acidified sample, the microcolumns were washed with 50 μl of 0.1% acetic acid, and elution of samples was performed with 50 μl of 80% (v/v) methanol (525 g, 20 min, 4°C). Eluates were then dried in vacuum and stored at −20°C. IAN and IAA metabolite levels were then determined using ultra‐high performance LC‐MS/MS (1290 Infinity LC system and a 6490 Triple Quadrupole LC/MS system; Agilent Technologies) using stable isotope‐labelled internal standards as a reference (Rittenberg and Foster, 1940). Four independent biological replicates were analysed. Statistical differences were determined by pair‐wise anova.

## Author Contributions

LvdW and MvZ designed, conceived and coordinated the study, performed experiments and wrote the manuscript. GK and NIM performed chemical synthesis and contributed to structure–activity relationship analysis. MP performed NIT1 activity assays and provided *nit1‐subfamily* mutants. JKP, DK, SN, FK, MK and RvdH contributed to the chemical proteomics experiments. LBS and MvV performed transcriptomics analysis. ON and KL performed IAN and IAA quantifications. LBS contributed to statistical and transcriptomics analyses. SR facilitated and supervised the chemical library screen. SB, SS and MF provided critical advice before and throughout the project. All authors commented and approved the manuscript.

## Conflict of interest

SB is an employee of Bejo Zaden B.V. Bejo made in kind contributions to the project but was not involved in the experimental design, data analysis or data presentation in the manuscript. Other authors declare that they have no competing interests.

## Supporting information

**Figure S1**. Chemical genetics screening to identify compounds that affect thermomorphogenesis.
**Figure S2**. Heatin stimulates thermomorphogenesis on the whole‐plant level.
**Figure S3**. Chemical structures of Heatin analogous compounds used for structure–activity relation studies.
**Figure S4**. G‐protein signalling complex mutants do not show altered Heatin sensitivity and Heatin analogue no. 205 does not induce hypocotyl elongation.
**Figure S5**. Characterization of *aao* mutants.
**Figure S6**. Heatin and picloram activity require partly similar and distinct auxin signalling components.
**Figure S7**. Kinetics of Heatin‐induced hypocotyl elongation in germinating and establishing seedlings.
**Figure S8**. Relative effects of Heatin on hypocotyl elongation during seed germination and seedling growth.
**Figure S9**. Heatin effects on the seedling transcriptome.
**Figure S10**. Proteins interacting with Heatin and Heatin effects on crop varieties.
**Figure S11**. *Nit1‐subfamility* mutants exhibit reduced sensitivity to Heatin but remain sensitive to HNA and HNC.
**Figure S12**. Validation of chemical proteomics and NIT1 activity assays.
**Figure S13**. Heatin effects on auxin metabolite levels.
**Figure S14**. *Cyp79b2* and *cyp79b3* mutants exhibit reduced sensitivity to high temperature and are resistant to Heatin‐induced hypocotyl elongation.

**Table S1**. Small molecule screening data.
**Table S2**. Quantification of effects of candidate hit compounds (34 compounds) isolated based on initial visual screening (8360 compounds).
**Table S3**. Potential hit compounds from the chemical genetics screen with consistent effect.
**Table S4**. Chemical compounds used in the structure–activity relation study.
**TableS5**. Numbers of significantly differentially regulated genes.
**TableS6**. Significantly differentially regulated genes in 2‐day‐old Heatin‐treated seedlings.
**TableS7**. Normalized RNA‐seq read counts of genes differentially regulated by Heatin and high temperature in 7‐day‐old seedlings.
**TableS8**. GO‐terms of biological processes significantly enriched amongst Heatin regulated genes after 7 days.
**TableS9**. Change in expression (Log2) of selected auxin biosynthesis, perception and signalling genes.
**TableS10**. Gene identifiers of proteins significantly enriched in the ‘Elute’ fraction.
**TableS11**. GO‐term enrichment analysis of significantly enriched proteins in the ‘Heatin‐eluted’ fraction based on their molecular function.
**Table S12**. Primers used in this study.

**Appendix S1**. Transcriptomics of Heatin responsiveness.
**Appendix S2**. Chemical synthesis of azide‐functionalized probe (*N*‐(6‐azidohexyl)‐4‐((benzyl((2‐hydroxynaphthalen‐1 yl)methyl)amino)methyl)benzamide).
**Appendix S3**. Heatin stimulates growth of *Brassicaceae* varieties specifically.
**Appendix S4**. Chemical synthesis of Heatin (Ethyl naphthalen‐1‐ylalaninate).

**Data S1**. Processed (Perseus v1.6.2.1) data of targeted proteomics.

## Data Availability

Transcriptomics data have been deposited in the GEO repository (https://www.ncbi.nlm.nih.gov/geo/) under accession code GSE130964. MS proteomics data have been deposited to the ProteomeXchange Consortium via the PRIDE partner repository (https://www.ebi.ac.uk/pride/archive/) under identifier PXD015411. Other data supporting the findings of this study are available within the paper and its Supporting information files and/or are available from the corresponding author upon reasonable request. Generated biological materials are available from the corresponding author upon reasonable request.
