# Supplementary material for: The chemical compound ‘Heatin’ stimulates hypocotyl elongation and interferes with the Arabidopsis NIT1‐subfamily of nitrilases
Source: Plant J. 2021 May 6;106(6):1523–40. doi: 10.1111/tpj.15250 (PMC8360157; doi:10.1111/tpj.15250)
Supplement: Supplementary file 1 — Figure S1. Chemical genetics screening to identify compounds that affect thermomorphogenesis. Figure S2. Heatin stimulates thermomorphogenesis on the whole‐plant level. Figure S3. Chemical structures of Heatin analogous compounds used for structure–activity relation studies. Figure S4. G‐protein signalling complex mutants do not show altered Heatin sensitivity and Heatin analogue no. 205 does not induce hypocotyl elongation. Figure S5. Characterization of aao mutants. Figure S6. Heatin and picloram activity require partly similar and distinct auxin signalling components. Figure S7. Kinetics of Heatin‐induced hypocotyl elongation in germinating and establishing seedlings. Figure S8. Relative effects of Heatin on hypocotyl elongation during seed germination and seedling growth. Figure S9. Heatin effects on the seedling transcriptome. Figure S10. Proteins interacting with Heatin and Heatin effects on crop varieties. Figure S11. Nit1‐subfamility mutants exhibit reduced sensitivity to Heatin but remain sensitive to HNA and HNC. Figure S12. Validation of chemical proteomics and NIT1 activity assays. Figure S13. Heatin effects on auxin metabolite levels. Figure S14. Cyp79b2 and cyp79b3 mutants exhibit reduced sensitivity to high temperature and are resistant to Heatin‐induced hypocotyl elongation. [file TPJ-106-1523-s003.docx]

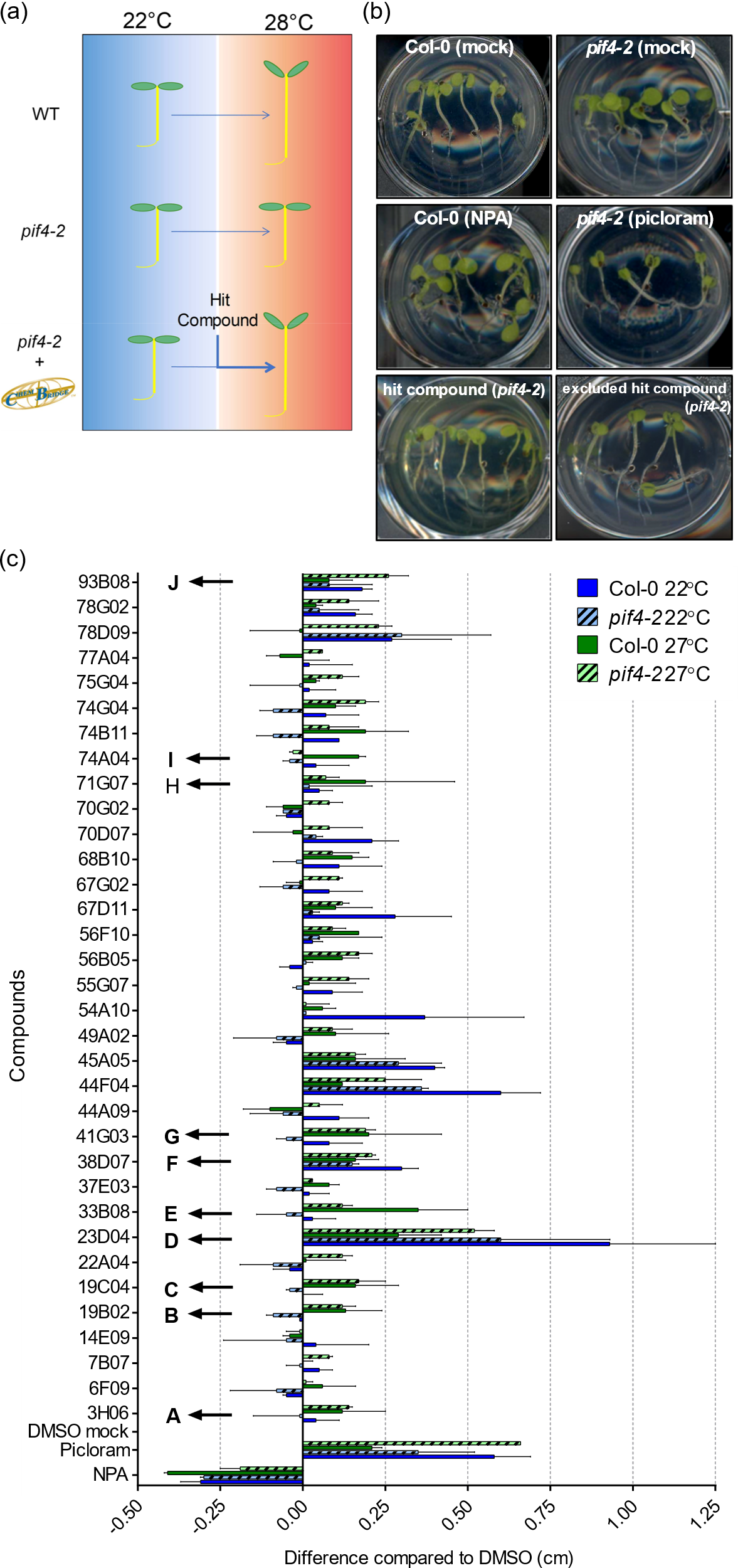


**Figure S1. Chemical genetics screening to identify compounds that affect thermomorphogenesis.** (a) Schematic representation of the screening strategy. Potential ‘hit’ (candidate) compounds were deemed able to rescue impaired hypocotyl elongation of the *pif4-2* mutant at warm temperatures (28°C). (b) Typical hypocotyl elongation phenotypes of nine day-old seedlings observed in the screening (all at 28°C). Indicated are added chemicals and genotype of the seedlings. DMSO was used as solvent mock treatment. Picloram and NPA were added at a final concentration of 4.18 µM and functioned as positive (induction of elongation in *pif4-2* mutant) and negative (repression of elongation of Col-0 wildtype) controls, respectively. Note that a compound was considered as a hit, only when the compound induced hypocotyl elongation without affecting the orientation of growth relative to the gravity vector. (c) Quantification of hypocotyl lengths of eight day-old seedlings in the presence of candidate hit compounds (34 compounds) isolated from the initial visual screening (8360 compounds), shown as absolute difference compared to the DMSO solvent mock control in the Col-0 wildtype (clear bars) and *pif4-2* mutants (dashed bars) at 22°C (blue bars) and 27°C (green bars). See also Table S2. NPA and picloram were included as controls. Arrows show 10 compounds chosen for further analyses based on reproducibility of effects, with their given ID (#I-J). Note that compound ‘D’ is Heatin. All compounds were added at 8.5 µM final concentration, except for NPA and picloram (4.18 µM). Error bars indicate SEM.


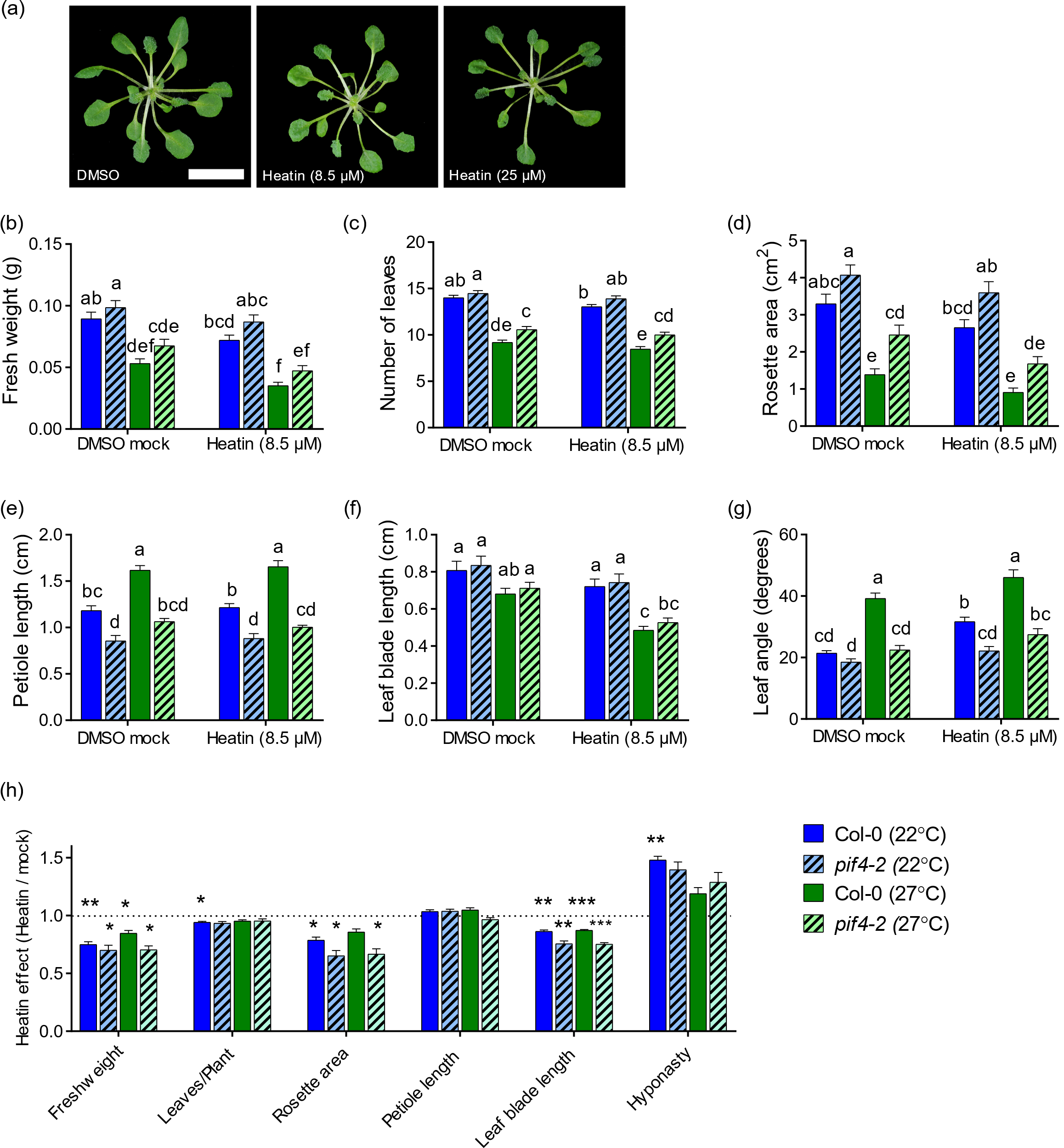


**Figure S2. Heatin stimulates thermomorphogenesis on the whole-plant level.** (a) Representative five week-old rosettes of Col-0 wildtype plants grown at 22°C on medium containing DMSO mock solvent (left) or Heatin (8.5 µM; middle and 25 µM; right). Scale bar equals 1 cm. (b-g) phenotypic traits associated with thermomorphogenesis of Col-0 wildtype (clear bars) and *pif4-2* mutant (dashed bars), grown on DMSO mock or Heatin-containing medium in control (22°C; blue bars) and high temperature (27°C; green bars) conditions. (b) Fresh weight, (c) leaf number, (d) rosette area, (e) petiole length, (f) leaf blade length, and (g) leaf angle relative to the horizontal (hyponastic growth), were quantified at the onset of bolting. Letters indicate significance groups per trait (Tukey HSD post-hoc tests), where averages that do not share letters are significantly different from each other (p<0.05). (h) Relative Heatin effect (compared to the mock (DMSO) treatment) on traits described in panel (b-g) The dotted horizontal line denotes no effect of Heatin. Asterisks indicate significant differences from 1 (*i.e.* no Heatin effect) as determined by one-sample t-test; *p<0.05, **p<0.01, ***p<0.001. Values are averages of 7 replicates of each 8-12 plants. Errors bars indicate SEM.


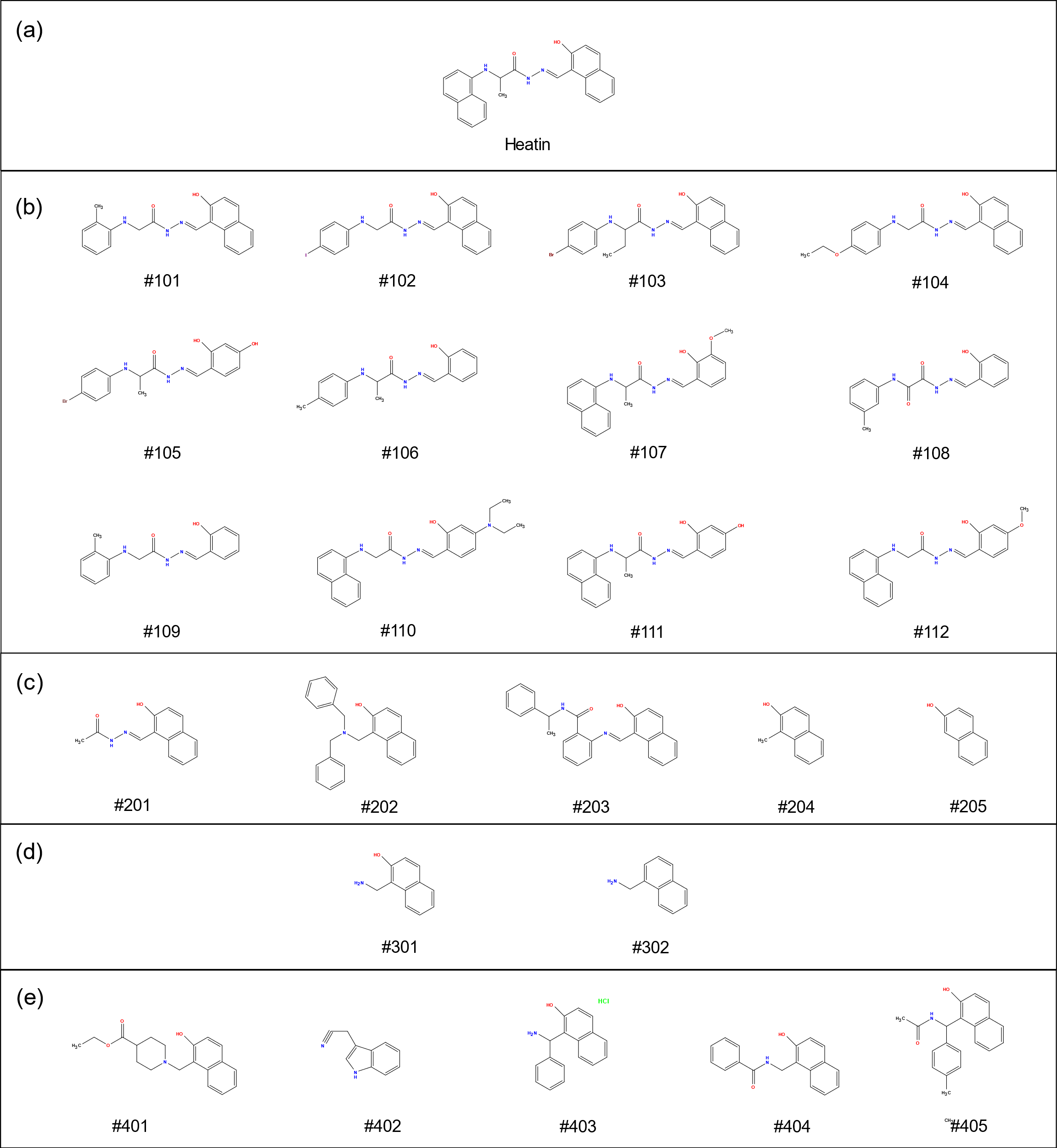


**Figure S3. Chemical structures of Heatin analogous compounds used for structure activity relation studies** (a-e), with their given compound ID (number). For chemical names see Table S4. Note that compound #203 is Sirtinol and #402 is the auxin precursor IAN.


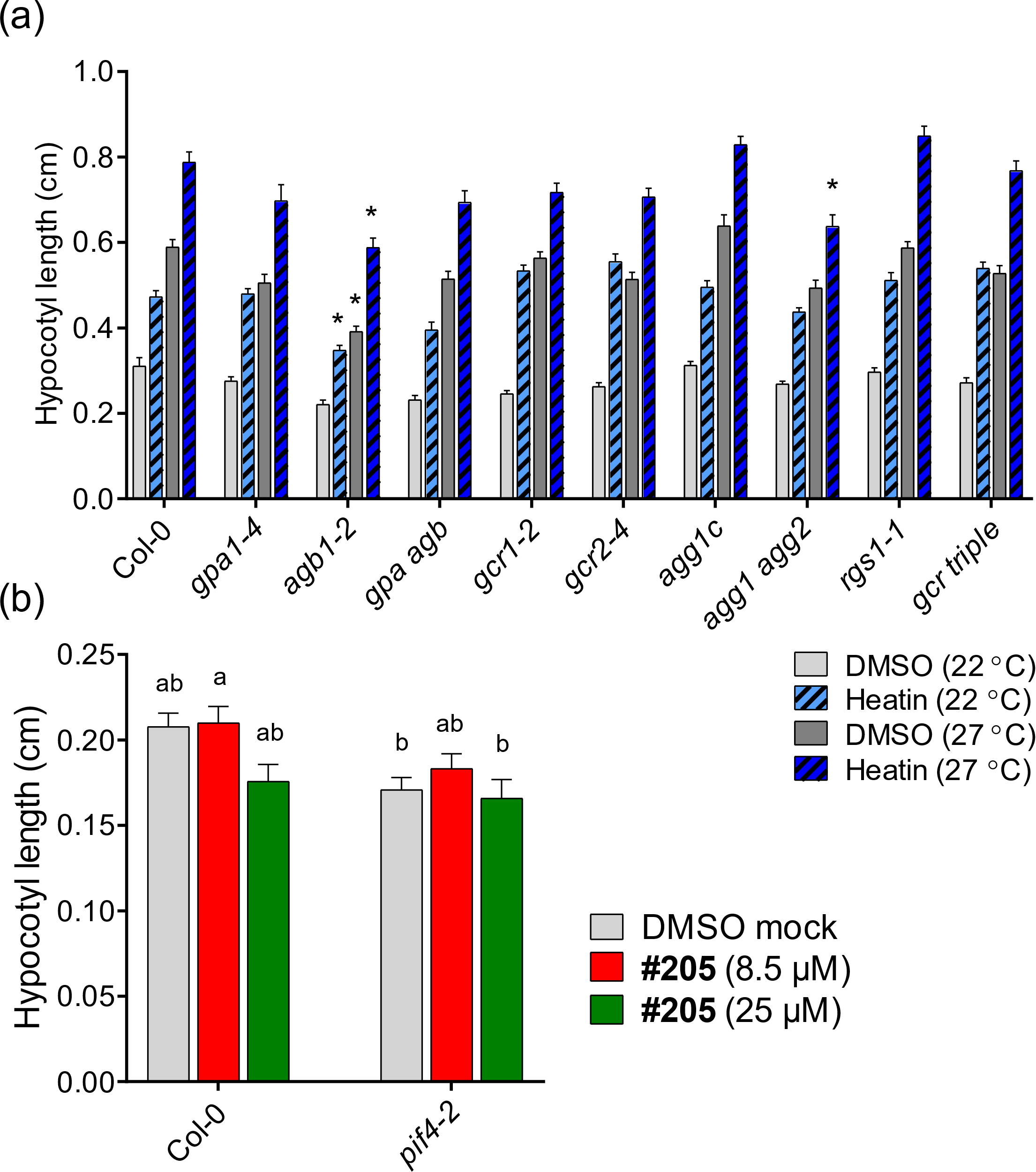


**Figure S4. G-protein signaling complex mutants do not show altered Heatin sensitivity and Heatin analogue #205 does not induce hypocotyl elongation.** (a,b) Hypocotyl length of eight day-old seedlings of Col-0 wildtype and indicated G-protein signaling mutants on (a) mock (DMSO solvent; clear grey bars) or 8.5 µM Heatin (blue, dashed bars) at 22°C (light bars) or 27°C (dark bars) and (b) mock (DMSO solvent; grey bars) or analogue #205 (8.5 µM, red bars; 25 µM, green bars) at 22°C. Values are averages of (a) two replicates of each 15-25 seedlings and (b) 15 seedlings. Asterisks in panel (a) indicate significant difference compared to the Col-0 wildtype value (p<0.05; Student’s t-test) and letters in (b) indicate significance groups (Tukey HSD post-hoc test), where averages that do not share letters are significantly different from each other (p<0.05). Error bars indicate SEM.


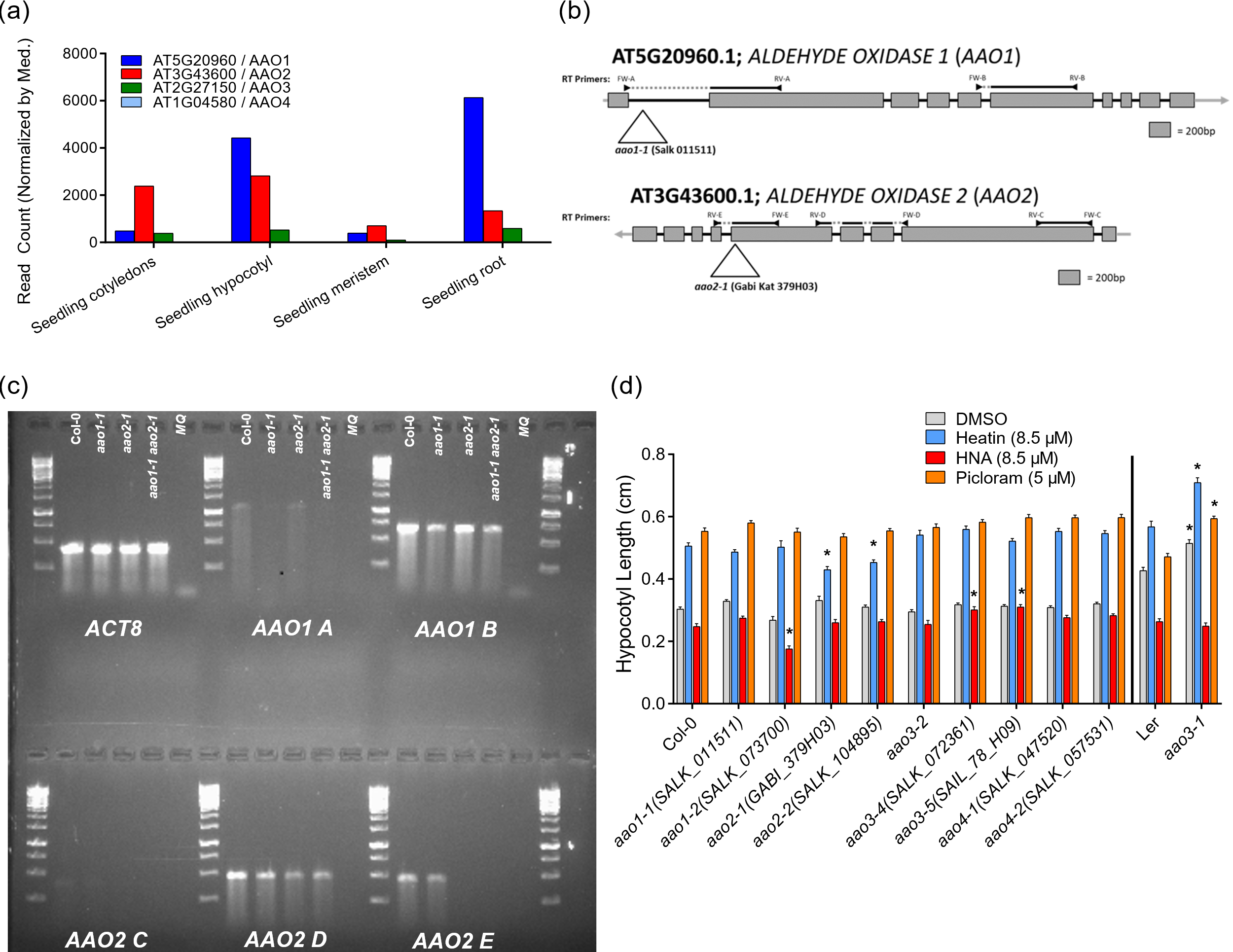


**Figure S5. Characterization of *aao* mutants.** (a) Normalized RNA-seq read counts of *AAO1* (dark blue bars)*, AAO2* (red bars)*, AAO3* (green bars) and *AAO4* (light blue bars) in young Arabidopsis seedlings. Note that *AAO4* read count is low (0.68- 11 in all indicated tissues). Data is derived from the TraVA database (http://travadb.org/). (b) Gene models for *AAO1* and *AAO2*, showing positions of T-DNA inserts (triangles) and RT-primers used for genotyping (indicated as A-E). (c) Agarose gel showing Reverse Transcriptase (RT)-PCR results (35 cycles) of one week-old whole seedlings. Tested genotypes are indicated above the lanes (Col-0, *aao1-1, aao2-1, aao1-1 aao2-*1). MQ indicates miliQ water (no cDNA) used as a negative control. Primer combinations used (A-E, see panel b) are indicated below the lanes. For used oligonucleotides see Table S11. *ACTIN8* (*ACT8*) was included as a positive control and the 1-kb gene-ruler (Thermo Fisher) is included as size marker. (d) Hypocotyl lengths of eight day-old seedlings of indicated *aao* mutant genotypes in the Col-0 and L*er* wildtype genetic backgrounds, grown on mock (DMSO solvent; grey bars), Heatin (8.5 µM; blue bars), HNA (8.5 µM; red bars) or picloram (5 µM; orange bars). Asterisks indicate significant difference compared to the Col-0 wildtype, respectively L*er* (*aoo3-1*) (p<0.05; Student’s t-test). Values are averages of 3 replicates of each 20-25 seedlings. Error bars indicate SEM.


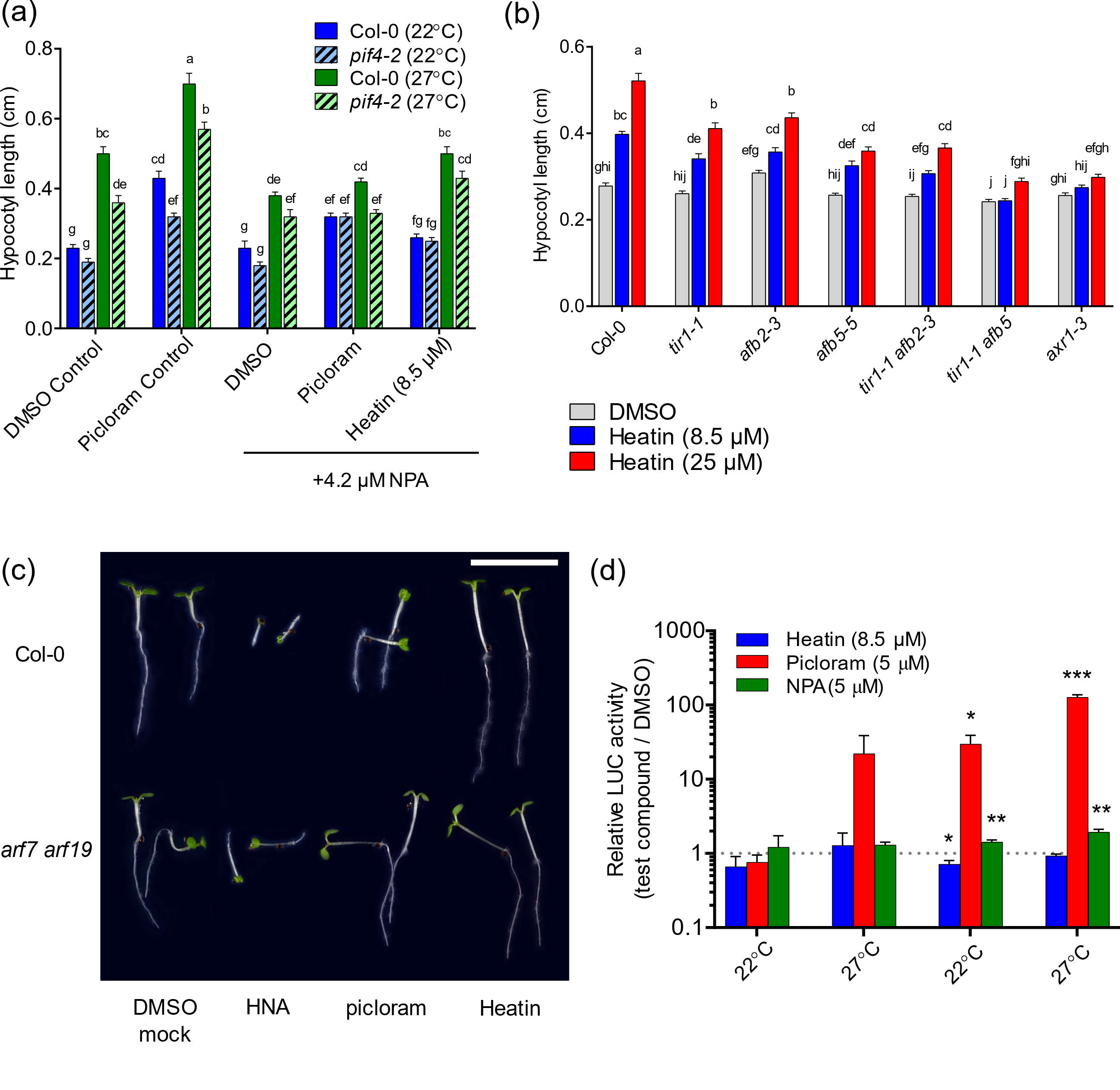


**Figure S6. Heatin and picloram activity require partly similar and distinct auxin signalling components.** (a) Hypocotyl lengths of eight day-old Col-0 wildtype (clear bars) and *pif4-2* mutant (dashed bars) seedlings, at 22°C (blue) and 27°C (green bars) on mock (DMSO solvent) and in presence of Heatin (8.5 µM) or picloram (8.5 µM), with and without additional NPA (4.2 µM). (b) Hypocotyl lengths of eight day-old seedlings of Col-0 wildtype and indicated auxin signalling mutants on mock (DMSO solvent; grey bars) or in presence of Heatin (8.5 µM; blue bars, 25 µM red bars). (a,b) Letters indicate significance groups (Tukey HSD post-hoc test), where averages that do not share letters are significantly different from each other (p<0.05). (a,b) Values are averages of (a) 10-15 seedlings and (b) 3-5 repeats of 15-25 seedlings each. Error bars indicate SEM. (c) Phenotypes of representative Col-0 wildtype (upper row) and *arf7 arf19* double mutant seedlings (lower row) grown on mock (DMSO solvent left), or in the presence of HNA (8.5 µM; middle-left), picloram (8.5 µM; middle-right), Heatin (8.5 µM; right). Scale bar indicates 0.5 cm. (d) LUCIFERASE activity of *eDR5:LUC* seedlings grown on medium containing Heatin (8.5 µM; blue bars), compared to DMSO mock-treated seedlings of 2- (left 2 groups of bars) and 6 day-old (right 2 groups of bars) seedlings, grown at the control (22°C) or high temperature (27°C). Picloram (5 µM; red bars) and 5 µM NPA (green bars)-treated seedlings are included as positive and negative control, respectively. The dotted horizontal line indicates no change compared to mock. Asterisks indicates significant difference from 1, as tested by one-sample t-test; * p<0.05, **p<0.01, ***p<0.001.


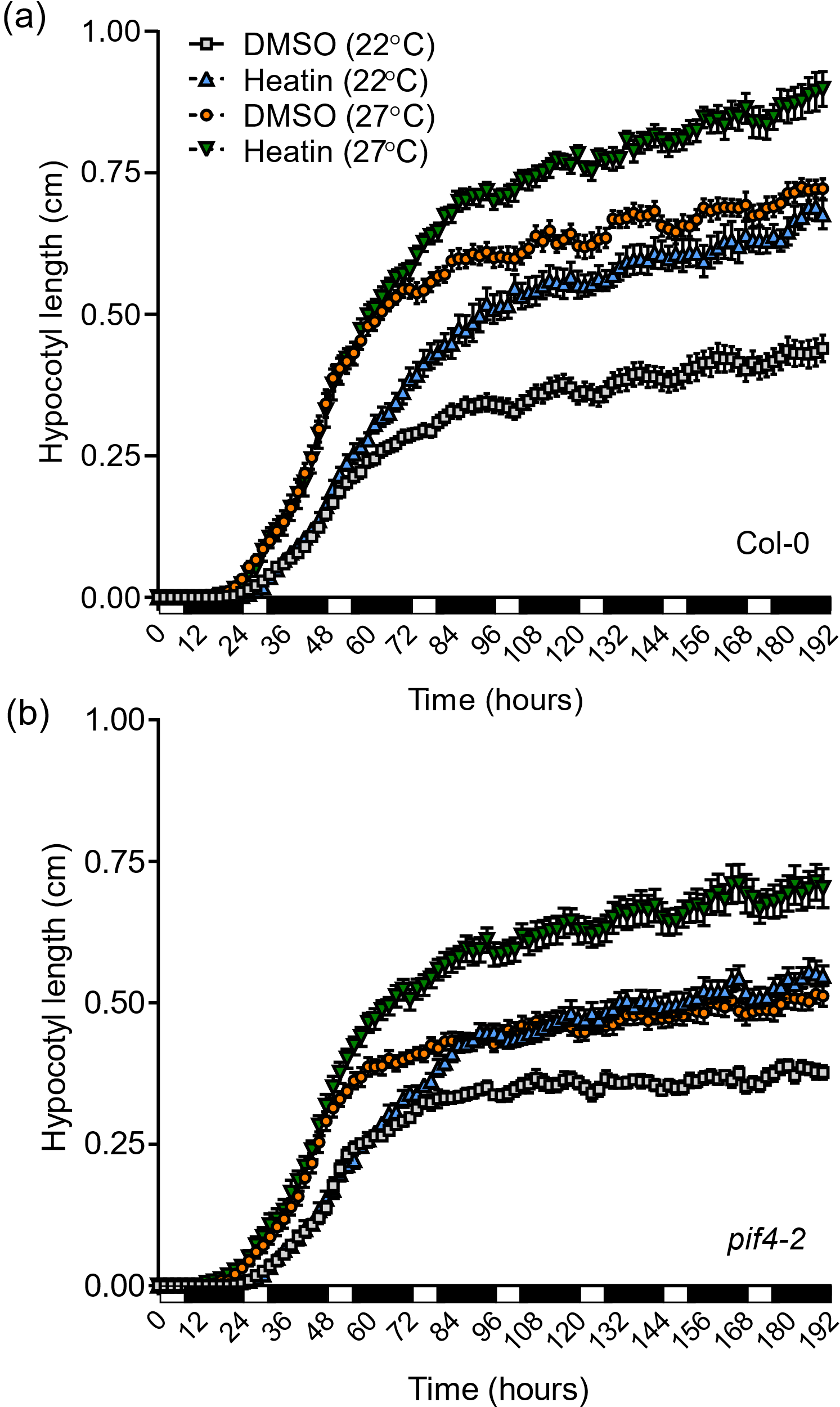


**Figure S7. Kinetics of Heatin-induced hypocotyl elongation in germinating and establishing seedlings.** (a,b) Hypocotyl growth of (a) Col-0 wildtype and (b) *pif4-2* mutant seedlings from seed (t=0 h) to eight day-old seedling (t=192 h) in the absence (mock DMSO solvent; squares and circles) and presence of 8.5 µM Heatin (triangles) at 22°C (grey and blue symbols) or 27°C (orange and green symbols). White and black bars on the X-axis indicate the light (day) and dark periods (night) of the short day photoperiod regime (8h light, 16h darkness). Values are averages of 12-18 independent repetitions of each 5-10 seedlings. Error bars indicate SEM. Note that seeds germinate at ~t=24 h.


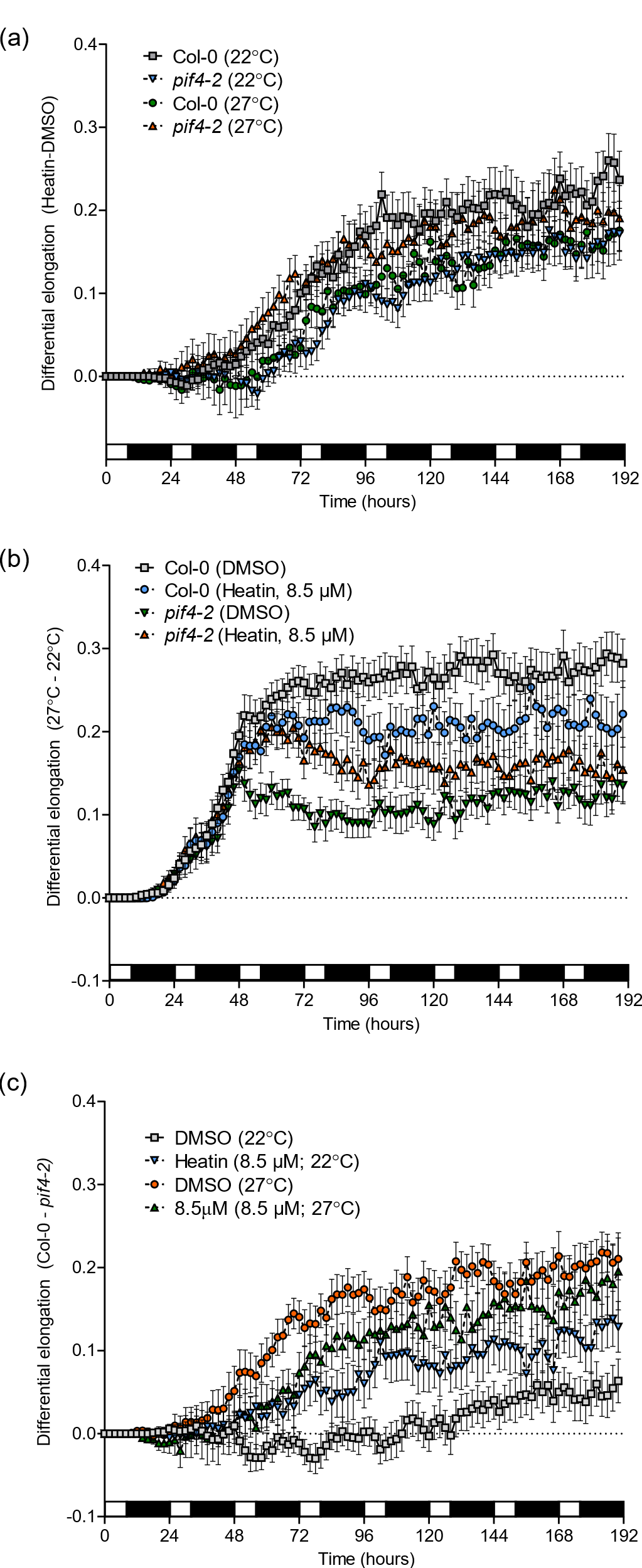


**Figure S8. Relative effects of Heatin on hypocotyl elongation during seed germination and seedling growth.** (a) Differential response of Heatin (8.5 µM) relative to DMSO solvent mock of Col-0 (squares and circles) and *pif4-2* mutant (triangles) seedlings, grown at either 22°C (grey and blue symbols) or 27°C (green and orange symbols). (b) Differential response of high temperature grown seedlings (27°C) relative to control temperature (22°C) of Col-0 (grey and blue symbols) and *pif4-2* (green and orange symbols) seedlings grown on either mock (DMSO solvent; squares and downward triangles) or in the presence of Heatin (8.5 µM; circles and upward triangles). (c) Differential response of Col-0 relative to *pif4-2* of mock (DMSO solvent; squares and circles) in the presence of Heatin (8.5 µM; triangles) at 22°C (squares and downward triangles symbols) or 27°C (circles and upward triangles). In all panels, progression of hypocotyl elongation of seedlings is shown from seed (t=0 h) to eight day-old seedling (t=192 h). White and black bars on the X-axes indicate the light (day) and dark periods (night) of the short day photoperiod regime (8h light, 16h darkness). Values are averages of 12-18 repeats of each 5-10 seedlings. Error bars indicate SEM. Note that seeds germinate at ~t=24 h. Dotted lines at Y=0 indicate no difference between compared two groups.


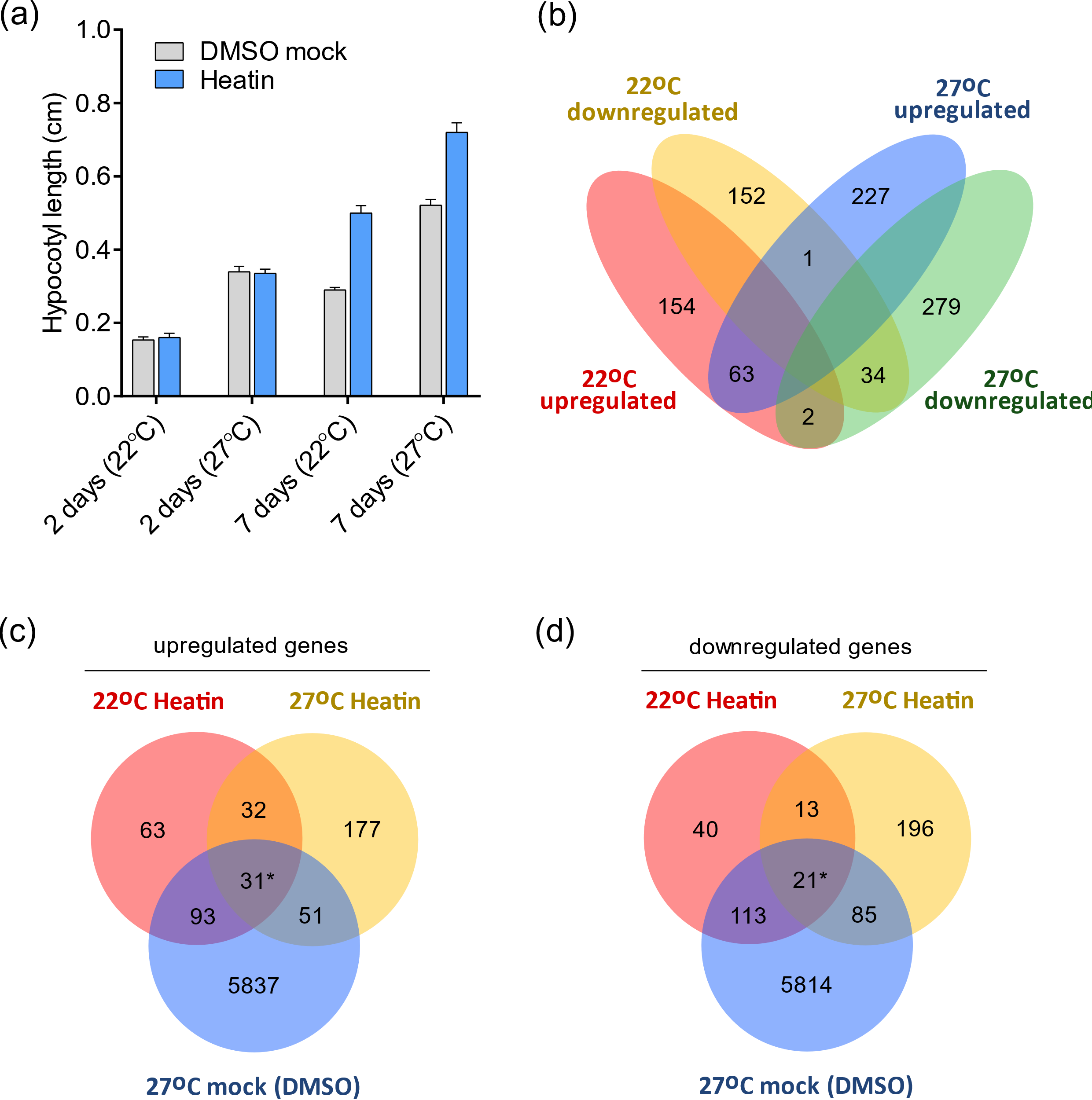


**Figure S9. Heatin effects on the seedling transcriptome**. See Appendix S1 for details. (a) Hypocotyl lengths of two and seven day-old seedlings used for RNA sequencing, grown at control (22°C) or high ambient temperature (27°C) on mock (DMSO solvent; grey bars) and in the presence of Heatin (8.5 µM; blue bars). Error bars indicate SEM. Values are averages of 12 replicates consisting of ~50-100 seedlings each. (b-d) Venn-diagrams showing numbers and overlap of differentially regulated genes. (b) Genes upregulated (blue and red) and downregulated (yellow and green) as a result of Heatin treatment (relative to mock) at control (22°C; yellow and red) and high temperature (27°C; green and blue) conditions. (c,d) Overlap between (c) upregulated and (d) downregulated genes relative to mock (DMSO solvent control) at 22°C. Shown are genes regulated by Heatin at 22°C (red), Heatin at 27°C (yellow) and genes affected by temperature in mock conditions (blue). Genes in the fields marked with asterisks are shown in Table S7. Numbers indicate the fraction of genes that were significantly differentially regulated in the indicated comparisons. Impossible combinations are left blank in panel (b) *e.g.* up and down simultaneously in the same condition.


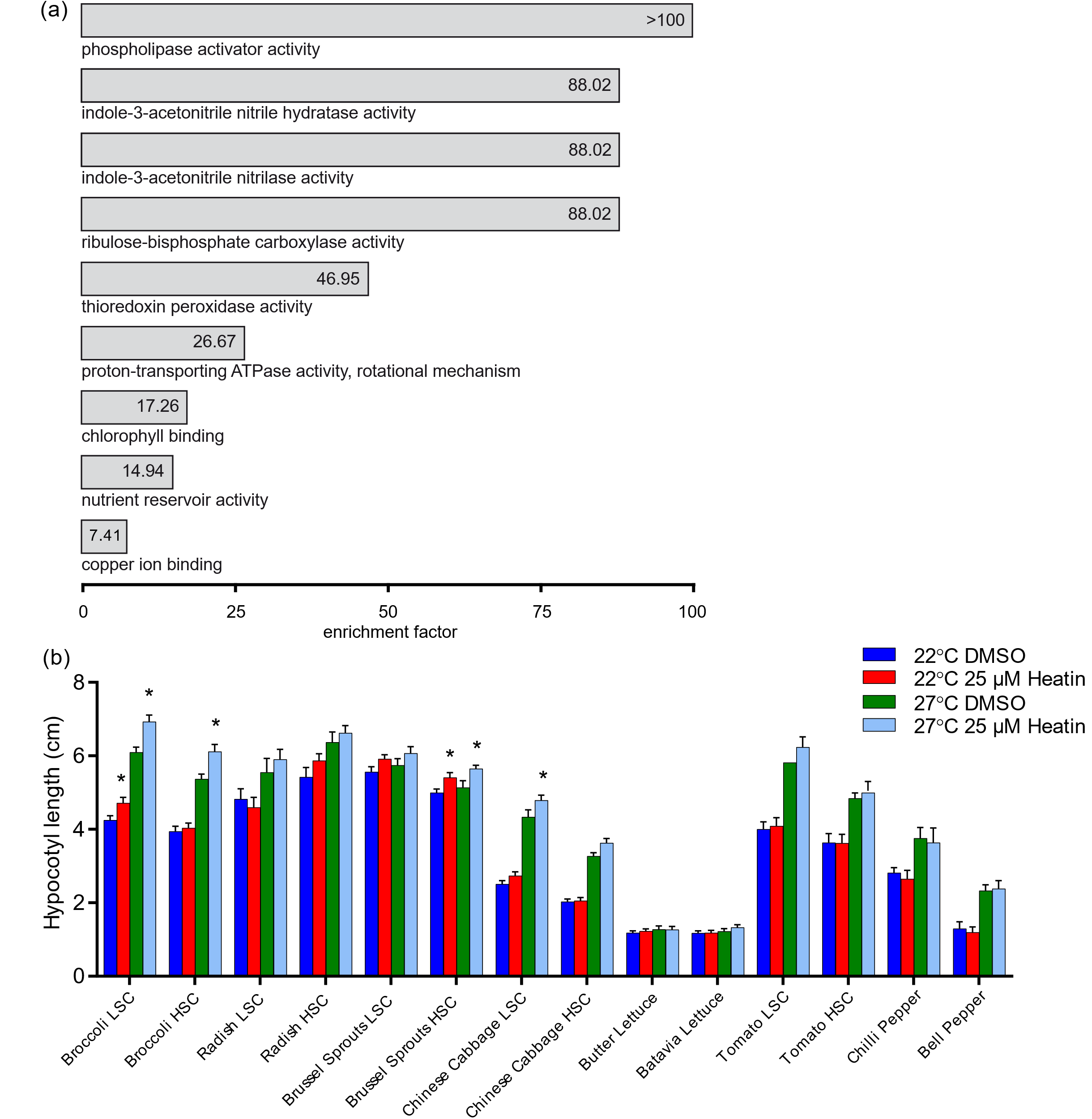


**Figure S10. Proteins interacting with Heatin and Heatin effects on crop varieties.** (a) Gene Ontology (GO) term enrichment analysis of significantly enriched proteins in the ‘Heatin-eluted’ fraction compared to the ‘On-bead’ fraction, based on their molecular function. Statistical overrepresentation is based on a Fisher‘s Exact test. Results with a Bonferroni-corrected p-value < 0.05 and a fold enrichment > 1 are displayed. For details, see Table S10. (b) Effect of Heatin application (25 µM; red and light blue bars) compared to (DMSO mock; dark blue and green bars) on hypocotyl elongation of 8 day-old seedlings (except bell pepper and chili pepper; 10 day-old) of diverse F1 hybrids of commercial crop varieties. Tested are batches with low (LSC) and high (HSC) seed count (seeds per gram^-1^) respectively, at control (22°C; dark blue and red bars) and high temperature conditions (27°C; green and light blue bars). Values are averages of 8 replicates of 20-30 seedlings each. Asterisks indicate significance of the Heatin effect compared to the mock treatment (p<0.05; Student’s t-test).


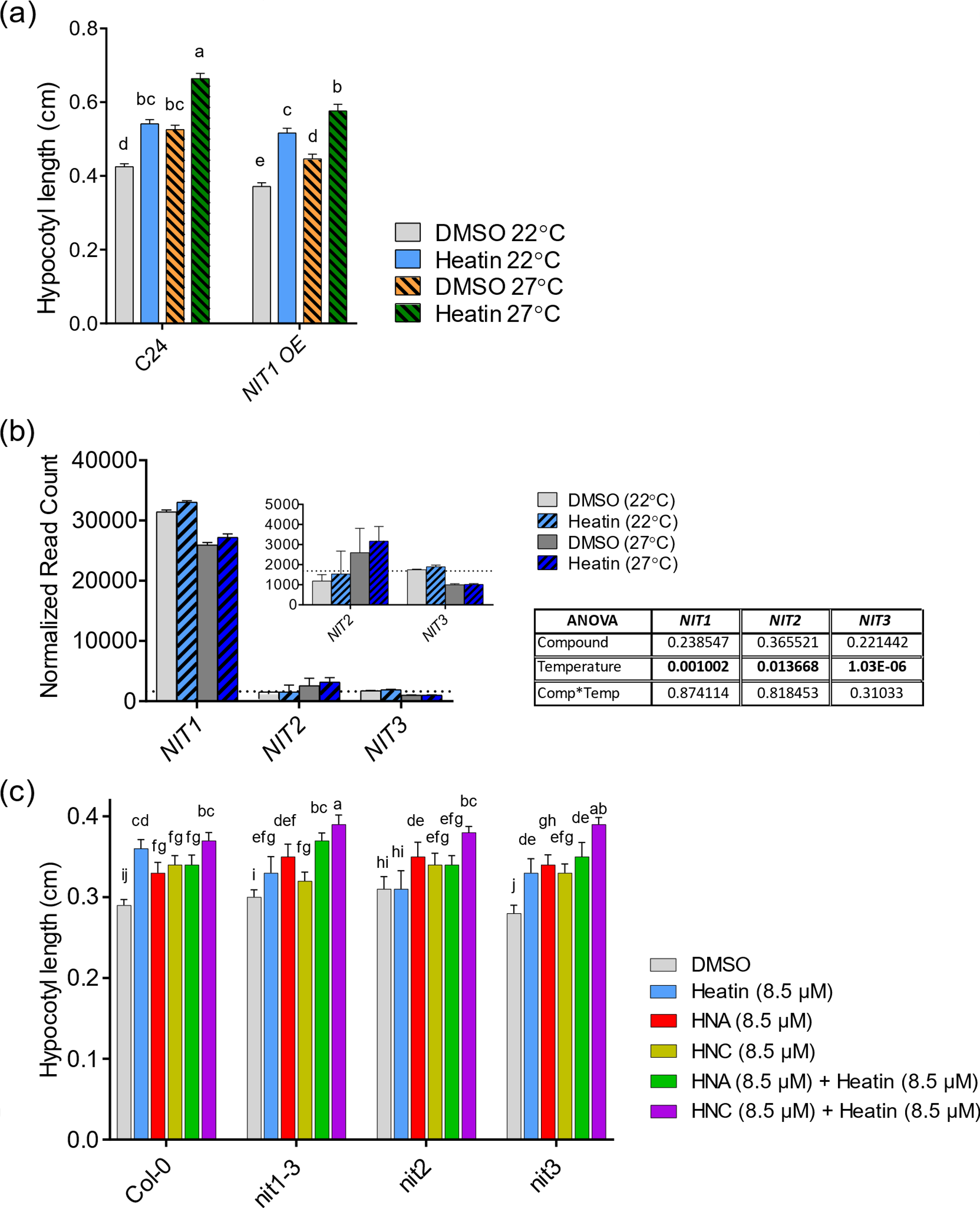


**Figure S11. *Nit1-subfamility* mutants exhibit reduced sensitivity to Heatin but remain sensitive to HNA and HNC.** (a) Hypocotyl lengths of eight day-old *NIT1 OE* (*NIT1* overexpression line) seedlings and its respective C24 wildtype background, on mock (DMSO, grey and orange bars) or in presence of Heatin (8.5 µM; blue and green bars), at either 22°C (open bars) or 27°C (dashed bars). Values are averages of 6 independent repetitions of 20-30 seedlings each. (b) Normalized read counts for *NIT1*, *NIT2* and *NIT3* as detected by RNA sequencing (Figure S9) in two day-old seedlings on mock (DMSO solvent; grey open bars) or in the presence of Heatin (8.5 µM, blue dashed bars), at 22°C (light bars) or 27°C (dark bars). The dotted horizontal line represents the average read count of all detected transcripts. Inset provides a magnification of *NIT2* and *NIT3* results. P values (ANOVA) of statistical comparisons for each *NIT* gene is indicated in the table below the graph. (c) Hypocotyl lengths of eight day-old *nitrilase1-subfamily* mutant seedlings on DMSO solvent (mock; grey bars), Heatin (8.5 µM; blue bars), HNA (8.5 µM; red bars), HNC (8.5 µM; yellow bars) and the combination of HNA and Heatin (both 8.5 µM; green bars) or HNC and Heatin (both 8.5 µM; purple bars). Values are averages of 11-12 independent repetitions of 15-50 seedlings each. (a-d) Error bars indicate SEM. Letters indicate significance groups (Tukey HSD post-hoc test), where averages that do not share letters are significantly different from each other (p<0.05).


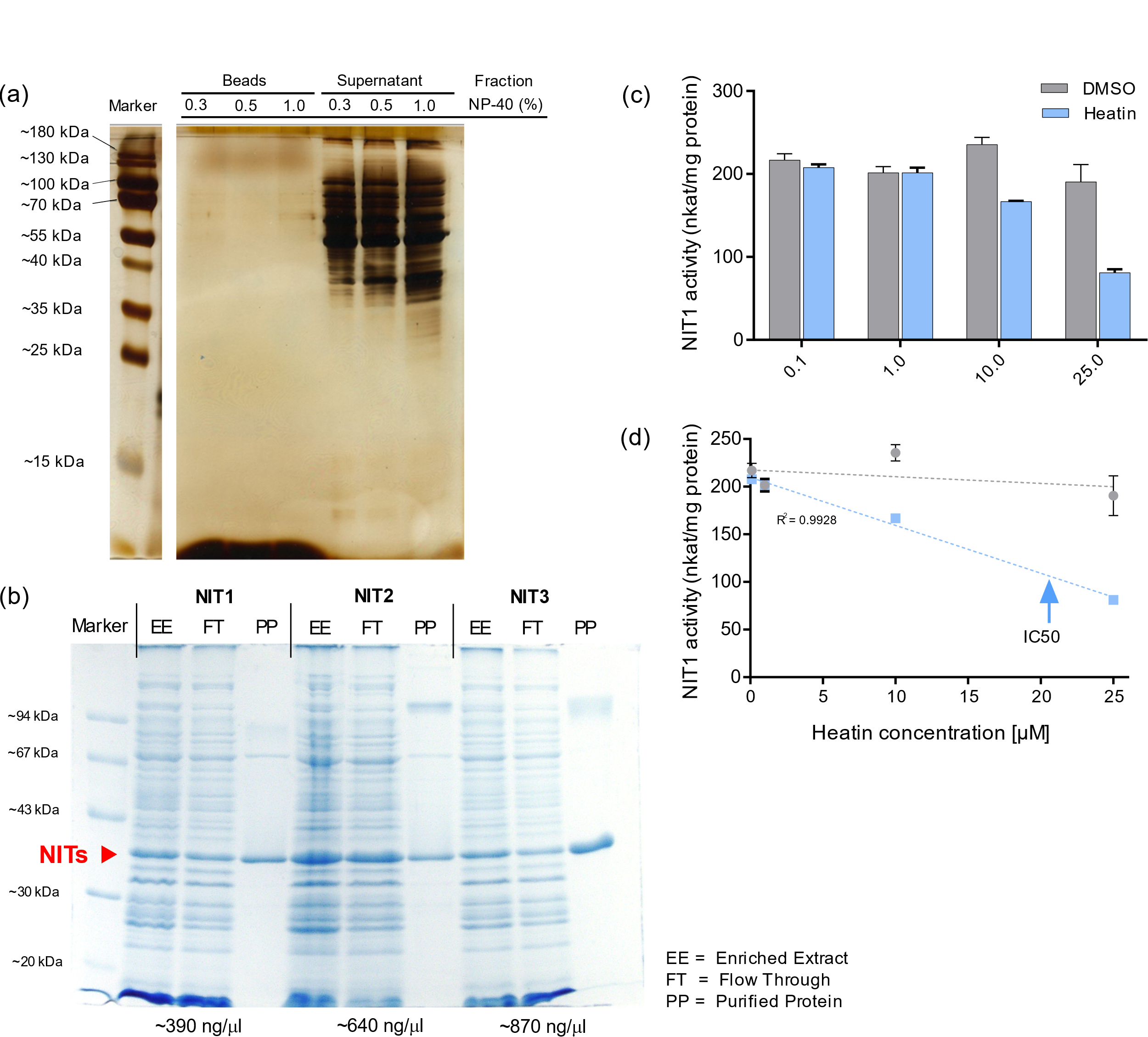


**Figure S12. Validation of chemical proteomics and NIT1 activity assays.** (a) Silver stain of pre-wash supernatant samples and post-wash ‘On-bead’ fractions of protein pulldown procedure, used to test for presence of proteins on the beads after washing. Different NP-40 percentages (v/v) were tested to optimize the washing solution. A size marker was included to visualize protein sizes. (b) Purification of recombinant HIS-tagged NIT proteins. Coomassie-stained gel showing different stages of the NIT purification process from *E. coli* cell culture. EE denotes the post-(NH4)_2_SO_4_ precipitation NIT protein enriched extracts, FT denotes the Ni-NTA column flow-through depleted of His-tagged NIT proteins and PP denotes the purified His-tagged NIT protein. Slight impurities can be seen in the gel, which correspond to *E. coli* proteins. A size marker with corresponding band sizes is included. The red arrowhead indicates the predicted size (~37 kDa) of recombinant His-tagged NIT proteins. Number depicted below the gel indicate protein concentrations of the purified protein (PP) as determined by Bradford’s assay. (c,d) Dose-response effects of Heatin inhibition of recombinant NIT1 enzymatic activity *in vitro,* with 2,5 µM 3-phenylpropionitrile (3-PPN) substrate. DMSO solvent (mock) is indicated with (c) grey bars and (d) grey circles and Heatin-treated samples with (c) blue bars and (d) blue squares. Values are averages of 3 technical replicates. (c,d) Error bars indicate SEM. (d) dotted lines represent the linear regression line through the data points. For Heatin effects the Pearson (R^2^ = 0.9928) is indicated. From this linear model, the half maximal inhibitory concentration of Heatin for NIT1 activity (IC_50_ of 3-phenylpropionitrile (3-PPN) substrate turnover), indicated with a blue arrow at 20.7 µM) was estimated (105.6 nkat/mg protein) by interpolation based on the average NIT1 activity in the DMSO Mock samples (211.2 ± 19,5 (StDEV) nkat/mg protein).


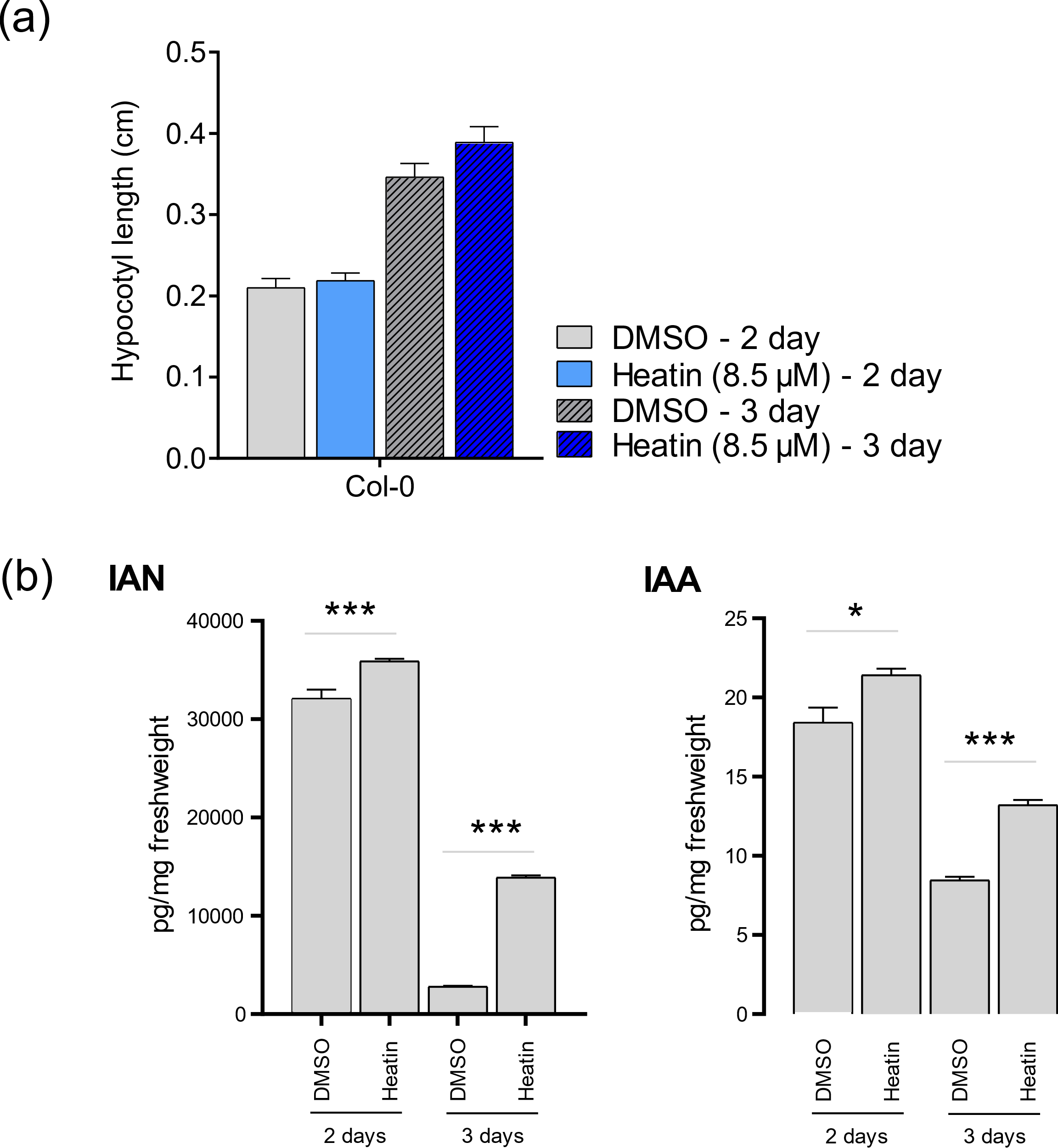


**Figure S13. Heatin effects on auxin metabolite levels.** (a) Hypocotyl lengths of two day-old (open bars) and three day-old (dashed bars) seedlings on mock (DMSO solvent; grey bars) or in the presence of Heatin (8.5 µM; blue bars), used for auxin metabolite analysis, confirming effectiveness and uniformness of the sampled materials. Error bars indicate SEM. Values are averages of 6 replicates consisting of ~50-100 seedlings each, of which four were used for auxin metabolite measurements. (b) Concentrations of IAN (left) and IAA (right) metabolites levels in two day-old and 3 day-old Col-0 wildtype seedlings on mock (DMSO control) and in the presence of Heatin (25 µM). N=4 replicates per treatment, each of 10 mg (fresh weight) seedlings. Asterisks indicate significant differences between mock and Heatin-treated samples, as determined by pair-wise ANOVA; *p<0.05, ***p<0.001.


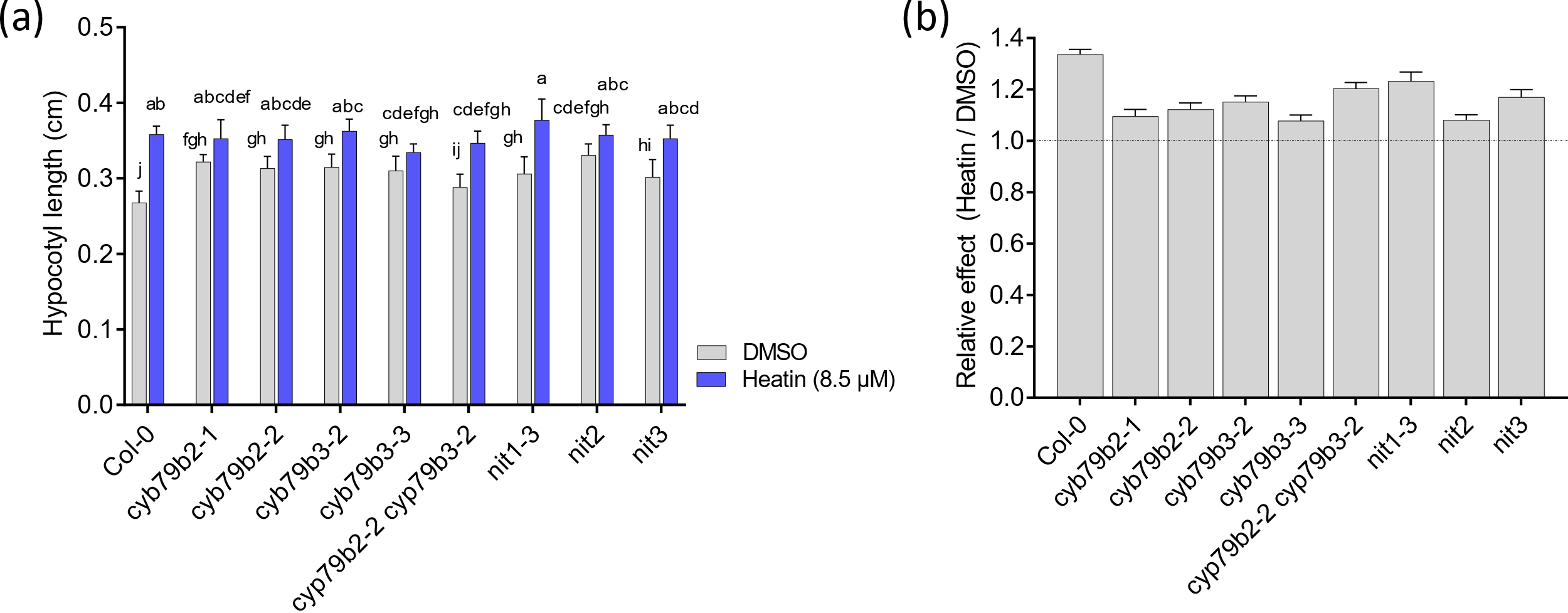


**Figure S14. *Cyp79b2 and cyp79b3* mutants exhibit reduced sensitivity to high temperature and are resistant to Heatin-induced hypocotyl elongation.** (b), Hypocotyl lengths of eight day-old seedlings of Col-0 wildtype and various *cyp79b2 and cypb79b3* and *nitrilase1-subfamily* mutants, grown in the presence of DMSO solvent (mock; grey bars) or Heatin (8.5 µM; blue bars), at 22°C. (b) Effect of 8.5 µM Heatin, relative to mock (DMSO) treatment, of hypocotyl lengths depicted in panel (a). The grey dashed line at Y=1 indicate no effect of Heatin. Error bars indicate SEM. Values are averages of 5 independent repetitions of 15-50 seedlings each. Letters indicate significance groups (Tukey HSD post-hoc test), where averages that do not share letters are significantly different from each other (p<0.05). Note that these data provide independent confirmation for the observation that *nit1-subfamily* mutants exhibit reduced sensitivity to Heatin.
