# Supplementary material for: The chemical compound ‘Heatin’ stimulates hypocotyl elongation and interferes with the Arabidopsis NIT1‐subfamily of nitrilases
Source: Plant J. 2021 May 6;106(6):1523–40. doi: 10.1111/tpj.15250 (PMC8360157; doi:10.1111/tpj.15250)
Supplement: Supplementary file 2 — Table S1. Small molecule screening data. Table S2. Quantification of effects of candidate hit compounds (34 compounds) isolated based on initial visual screening (8360 compounds). Table S3. Potential hit compounds from the chemical genetics screen with consistent effect. Table S4. Chemical compounds used in the structure–activity relation study. TableS5. Numbers of significantly differentially regulated genes. TableS6. Significantly differentially regulated genes in 2‐day‐old Heatin‐treated seedlings. TableS7. Normalized RNA‐seq read counts of genes differentially regulated by Heatin and high temperature in 7‐day‐old seedlings. TableS8. GO‐terms of biological processes significantly enriched amongst Heatin regulated genes after 7 days. TableS9. Change in expression (Log2) of selected auxin biosynthesis, perception and signalling genes. TableS10. Gene identifiers of proteins significantly enriched in the ‘Elute’ fraction. TableS11. GO‐term enrichment analysis of significantly enriched proteins in the ‘Heatin‐eluted’ fraction based on their molecular function. Table S12. Primers used in this study. [file TPJ-106-1523-s002.docx]

**Table S1.** **Small molecule screening data.**

| **Category** | **Parameter** | **Description** |
| --- | --- | --- |
| Assay | Type of assay | *In vitro,* 24-well plates containing *Arabidopsis thaliana* seedlings. |
|  | Target | Rescue of impaired hypocotyl elongation of *pif4-2* mutant seedlings at warm temperature conditions (28°C), *i.e*. molecular targets downstream or parallel to PIF4 signalling resulting in elongation growth. |
|  | Primary measurement | Visual screening of hypocotyl (embryonic stem) length. |
|  | Key reagents | Wells contained 1x Murashige-Skoog (MS) plant agar medium. Compounds dissolved in DMSO, with a final concentration of 0.1% in the medium. |
|  | Assay protocol | See the online methods section. In short; surface sterilized seeds were placed on MS-agar medium, containing the test compounds. Plates were stratified for three days, pre-germinated at 22°C, 100 µmol m^-2^ s^-1^ PAR light, 16 h photoperiod, for 24 h and then moved to a growth cabinet for eight days (28°C, 75 µmol m^-2^ s^-1^ PAR light, 8 h photoperiod, prior to visual screening. Initial hit compounds were rescreened (visually, 22°C and 28°C, 8 h photoperiod) and hypocotyl lengths of remaining hits were quantified by imageJ analysis software, using new/fresh compound obtained from the vendor. |
|  | Additional comments | NA |
| Library | Library size | 8000 random (of a total 17500) compounds + 360 known biologically active compounds in plants. |
|  | Library composition | Mainly aromatic druglike molecules covering a wide range of chemical space. |
|  | Source | Chembridge Corp. (17500 compound library) and Drakaki *et al.* 2011; Proc. Natl. Acad. Sci. U. S. A. 108, 17850–17855 (360 compound library) |
|  | Additional comments |  |
| Screen | Format | 24-well plates, each plate containing positive, neutral (mock solvent), and negative controls |
|  | Concentration(s) tested | 8.3 µM (compounds) |
|  | Plate controls | N-1-naphthylphthalamic Acid (NPA; Duchefa) and picloram (Sigma-Aldrich), dissolved in di-methyl sulfoxide (DMSO), were added to respectively Col-0 wildtype (negative control) and *pif4-2* (positive control) to a concentration of 4.18 µM to each 24-well plate for internal standardization. 0.1% DMSO lacking an active compound was used as (neutral) mock solvent control. |
|  | Reagent/compound dispensing system | Biomek NX, BeckmanCoulter pipetting robot. Seeds (6 per well) were placed manually |
|  | Detection instrument and software | Visual screen |
|  | Assay validation/QC | Hypocotyl length measurement using ImageJ image analysis software of seedlings scanned with a standard PC flatbed scanner. Internal plate controls were used for reference per plate |
|  | Correction factors | NA |
|  | Normalization | NA |
|  | Additional comments | NA |
| Post-library screening analysis | Hit criteria | Seedlings with a hypocotyl visually consistently longer than those of the mock control were considered initial hits. All compounds that resulted in a display of typical auxin-related phenotypes such as small, inward curved leaves, reduced root growth and agravitropic growth, in addition to hypocotyl elongation, were excluded from further analyses. |
|  | Hit rate | 298 primary hit compounds (visual screen; 3.56% of total); 38 compounds remaining after re-screening (visually) of the initial hit compounds (0.45% of total), 10 of these were further tested (0.12% of total), of which one (Heatin) was further characterized. |
|  | Additional assay(s) | NA |
|  | Confirmation of hit purity and structure | A structure-activity relation study was performed, confirming Heatin’s effectiveness. Moreover, both *in house* chemically synthesized Heatin and ‘fresh’ Heatin obtained from ChemBridge chemical vendor exhibited identical hypocotyl elongation growth stimulatory effects as the compound in the original tested chemical library. |
|  | Additional comments | NA |

**Table S2.** **Quantification of effects of candidate hit compounds (34 compounds) isolated based on initial visual screening (8360 compounds)**. See also Figure S1C. Shown are the library ID (left), absolute differences in hypocotyl length (cm) compared to the DMSO solvent mock control of Col-0 wildtype and *pif4-2* mutants at 22°C and 27°C and the given IDs (#I-J) of the 10 compounds chosen for further analyses, based on reproducibility of effects (See Table S3). The data is sorted from strongest to mildest effect on the *pif4-2* mutant at warm temperatures. Positive values are indicated with green shading, and negative values with red shading. Intensity of the shading reflects the relative effect-strength. NPA and picloram were included as controls. Note that NPA is a relatively strong inhibitor of hypocotyl elongation, whereas picloram induces hypocotyl elongation. Note that compound ‘D’ is Heatin. All compounds were added at 8.5 µM final concentration, except for NPA and picloram (4.18 µM).

| **Library** | **Col-0** | ***pif4-2*** | **Col-0** | ***pif4-2*** | **Given** |
| --- | --- | --- | --- | --- | --- |
| **ID** | **22^o^C** | **22^o^C** | **27^o^C** | **27^o^C** | **ID** |
| 23D04 | 0,93 | 0,60 | 0,29 | 0,52 | **D = Heatin** |
| 93B08 | 0,18 | 0,08 | 0,08 | 0,26 | **J** |
| 44F04 | 0,60 | 0,36 | 0,12 | 0,25 |  |
| 78D09 | 0,27 | 0,30 | -0,01 | 0,23 |  |
| 38D07 | 0,30 | 0,15 | 0,16 | 0,21 | **F** |
| 41G03 | 0,08 | -0,05 | 0,20 | 0,19 | **G** |
| 74G04 | 0,07 | -0,09 | 0,10 | 0,19 |  |
| 19C04 | 0,00 | -0,04 | 0,16 | 0,17 | **C** |
| 56B05 | -0,04 | 0,01 | 0,12 | 0,17 |  |
| 45A05 | 0,40 | 0,29 | 0,16 | 0,16 |  |
| 78G02 | 0,16 | 0,05 | 0,04 | 0,14 |  |
| 55G07 | 0,09 | -0,02 | 0,02 | 0,14 |  |
| 3H06 | 0,04 | -0,01 | 0,12 | 0,14 | **A** |
| 67D11 | 0,28 | 0,03 | 0,10 | 0,12 |  |
| 33B08 | 0,03 | -0,05 | 0,35 | 0,12 | **E** |
| 75G04 | 0,02 | -0,01 | 0,04 | 0,12 |  |
| 19B02 | -0,01 | -0,09 | 0,13 | 0,12 | **B** |
| 22A04 | -0,04 | -0,09 | 0,01 | 0,12 |  |
| 67G02 | 0,08 | -0,06 | -0,01 | 0,11 |  |
| 68B10 | 0,11 | -0,02 | 0,15 | 0,09 |  |
| 56F10 | 0,03 | 0,05 | 0,17 | 0,09 |  |
| 49A02 | -0,05 | -0,08 | 0,10 | 0,09 |  |
| 70D07 | 0,21 | 0,04 | -0,03 | 0,08 |  |
| 74B11 | 0,11 | -0,09 | 0,19 | 0,08 |  |
| 7B07 | 0,05 | -0,01 | 0,00 | 0,08 |  |
| 70G02 | -0,05 | -0,06 | -0,06 | 0,08 |  |
| 71G07 | 0,05 | 0,02 | 0,19 | 0,07 | **H** |
| 77A04 | 0,02 | 0,00 | -0,07 | 0,06 |  |
| 44A09 | 0,11 | -0,06 | -0,10 | 0,05 |  |
| 37000 | 0,02 | -0,08 | 0,08 | 0,03 |  |
| 54A10 | 0,37 | 0,01 | 0,06 | 0,01 |  |
| 6F09 | -0,05 | -0,08 | 0,06 | 0,01 |  |
| 14E09 | 0,04 | -0,05 | -0,04 | -0,01 |  |
| 74A04 | 0,04 | -0,04 | 0,17 | -0,03 | **I** |
| NPA | -0,31 | -0,30 | -0,41 | -0,19 |  |
| Picloram | 0,58 | 0,35 | 0,21 | 0,66 |  |

**Table S3. Potential hit compounds from the chemical genetics screen with consistent effect.** Shown are given compound ID (letter), Vendor ID, molecular structure, name, molecular weight (MW) and Partition coefficient (cLogP), of 10 compounds selected after quantitative effect confirmation (see Figure S1). Data extracted from Hit2Lead.com chemical database.

| Compound: **A**  4-[(4-methyl-1-piperazinyl)sulfonyl]benzoic acid  Vendor ID: 5156996  MW: 284  CLogP: -0.49  Vendor ID5156996  MW 284  cLogP -0.49 | Compound: **B**  4-(dimethylamino)-N-[(1-ethyl-2-pyrrolidinyl)methyl]-2-methoxy-5-nitrobenzamide  Vendor ID: 5685297  MW: 350  CLogP: 2.68 |
| --- | --- |
| Compound:  **C**  2-hydroxy-N'-(1-isopropyl-4-piperidinylidene)-2-phenylacetohydrazide  Vendor ID: 5688969  MW: 289  CLogP: 3.52 | Compound: **D**  N'-[(2-hydroxy-1-naphthyl)methylene]-2-(1-thylamino)propanohydrazide  Vendor ID: 5713980  MW: 383  CLogP: 4.38 |
| Compound: **E**  2-[5-(5-bromo-2-oxo-1,2-dihydro-3H- indol-3-ylidene)-4-oxo-2-thioxo- 1,3-thiazolidin-3-yl]succinic acid  Vendor ID: 5364749  MW: 457  CLogP: 0.89 | Compound: **F**  methyl 5-bromo-2-{[(2-methylphenoxy)acetyl] amino}benzoate  Vendor ID: 6148371  MW: 378  CLogP: 4.55 |
| Compound: **G**  4-chloro-2-({[1,3-dioxo-2-(3-pyridinylmethyl)- 2,3-dihydro-1H-isoindol-5-yl]carbonyl}amino)benzoic acid  Vendor ID: 6131881  MW: 428  CLogP: 1.16 | Compound: **H**  N-[1-(3-methylbenzyl)-1H-1,2,4-triazol-3-yl]-2-(3-methyl-4-nitrophenoxy)acetamide  Vendor ID: 7674471  MW: 504  CLogP: 2.963 |
| Compound: **I**  4-chloro-2-({[1,3-dioxo-2-(3-pyridinylmethyl)-2,3-dihydro-1H-isoindol-5-yl]carbonyl}amino)benzoic acid  Vendor ID: 7724721  MW: 436  CLogP: 3.997 | Compound: **J**  N-[1-(3-methylbenzyl)-1H-1,2,4-triazol-3-yl]-2-(3-methyl-4-nitrophenoxy)acetamide  Vendor ID: 7948967  MW: 381  CLogP: 3.857 |

**Table S4. Chemical compounds used in the structure-activity relation study**. Indicated are given analogue ID, vendor, vendor ID and chemical names.

| **Analogue ID** | **Vendor** | **Vendor ID** | **Chemical name** |
| --- | --- | --- | --- |
| Heatin | Chembridge | 5713980 | N'-[(2-hydroxy-1-naphthyl)methylene]-2-(1-naphthylamino)propanohydrazide |
| NPA | Sigma-Aldrich | N12507 | N-(1-Naphthyl)phthalamidic acid |
| picloram | Sigma-Aldrich | P5575 | 4-Amino-3,5,6-trichloropicolinic acid |
| #101 | Chembridge | 5539488 | N'-[(2-hydroxy-1-naphthyl)methylene]-2-[(2-methylphenyl)amino]acetohydrazide |
| #102 | Chembridge | 5248195 | N'-[(2-hydroxy-1-naphthyl)methylene]-2-[(4-iodophenyl)amino]acetohydrazide |
| #103 | Chembridge | 5713195 | 2-[(4-bromophenyl)amino]-N'-[(2-hydroxy-1-naphthyl)methylene]butanohydrazide |
| #104 | Chembridge | 5723320 | 2-[(4-ethoxyphenyl)amino]-N'-[(2-hydroxy-1-naphthyl)methylene]acetohydrazide |
| #105 | Chembridge | 5712115 | 2-[(4-bromophenyl)amino]-N'-(2,4-dihydroxybenzylidene)propanohydrazide |
| #106 | Chembridge | 5559595 | 2-[2-(2-hydroxybenzylidene)hydrazino]-N-(4-methylphenyl)-2-oxoacetamide |
| #107 | Chembridge | 5725603 | N'-(2-hydroxy-3-methoxybenzylidene)-2-(1-naphthylamino)propanohydrazide |
| #108 | Chembridge | 6143663 | 2-[2-(2-hydroxybenzylidene)hydrazino]-N-(2-methylphenyl)-2-oxoacetamide |
| #109 | Chembridge | 5530357 | N'-(2-hydroxybenzylidene)-2-[(2-methylphenyl)amino]acetohydrazide |
| #110 | Chembridge | 5861253 | N'-[4-(diethylamino)-2-hydroxybenzylidene]-2-(2-naphthylamino)acetohydrazide |
| #111 | Chembridge | 5717758 | N'-(2,4-dihydroxybenzylidene)-2-(1-naphthylamino)propanohydrazide |
| #112 | Chembridge | 5864129 | N'-(2-hydroxy-4-methoxybenzylidene)-2-(1-naphthylamino)acetohydrazide |
| #201 | Sigma-Aldrich | S779644 | acetic (2-​hydroxy-​1-​naphthylmethylene)​hydrazide |
| #202 | Sigma-Aldrich | OTV000008 | 1-​[(Dibenzylamino)​methyl]​-​2-​naphthol |
| #203 / Sirtinol | Sigma-Aldrich | S7942 | Sirtinol |
| #204 | Chembridge | 4021629 | 1-methyl-2-naphthol |
| #205 | Sigma-Aldrich | 185507 | 2-Naphthol |
| #301 | Sigma-Aldrich | CDS019203 | 1-Aminomethyl-naphthalen-2-ol |
| #302 | Sigma-Aldrich | 127035 | 1-Naphthylmethylamine |
| #401 | Chembridge | 5429881 | ethyl 1-[(2-hydroxy-1-naphthyl)methyl]-4-piperidinecarboxylate |
| #402 / IAN | Sigma-Aldrich | 129453 | 3-Indoleacetonitrile |
| #403 | Sigma-Aldrich | 732400 | 1-(α-Aminobenzyl)-2-naphthol hydrochloride |
| #404 | Chembridge | 5114438 | N-[(2-hydroxy-1-naphthyl)methyl]benzamide |
| #405 | Chembridge | 5929093 | N-[(2-hydroxy-1-naphthyl)(4-methylphenyl)methyl]acetamide |
| HNA | Sigma-Aldrich | H45353 | 2-Hydroxy-1-naphthaldehyde |
| HNC | Sigma-Aldrich | H45809 | 2-Hydroxy-1-naphthoic acid |

**Table S5.** **Numbers of significantly differentially regulated genes.** Numbers represent genes of which the expression is significantly changed in our transcriptomics experiments (RNA-sequencing) in the indicated comparison in two day-old (2d) and seven day-old (7d) seedlings. Upregulated genes are shown in green letters, downregulated genes as red letters, and total of regulated genes in black, with and without a two-fold cut-off.

| **Comparison** | **Upregulated (no cut-off)** | | **Downregulated** | | **Total** | |
| --- | --- | --- | --- | --- | --- | --- |
|  | **cut-off >2 fold** | **no cut-off** | **cut-off >2 fold** | **no cut-off** | **cut-off >2 fold** | **no cut-off** |
| 2d: 27°C vs. 22°C | 2062 | 6521 | 2078 | 6579 | 4140 | 13100 |
| 7d: 27°C vs. 22°C | 1032 | 6012 | 1431 | 6033 | 2463 | 12047 |
| 2d 22°C: Heatin vs. DMSO | 0 | 2 | 0 | 0 | 0 | 2 |
| 2d 27°C: Heatin vs. DMSO | 5 | 8 | 1 | 2 | 6 | 10 |
| 7d 22°C: Heatin vs. DMSO | 26 | 219 | 23 | 187 | 49 | 406 |
| 7d 27°C: Heatin vs. DMSO | 86 | 291 | 107 | 315 | 193 | 606 |

**Table S6. Significantly differentially regulated genes in two day-old Heatin treated seedlings.** Shown are the temperature condition where the respective genes were regulated, the Arabidopsis gene identifier (AGI code), gene product, description of the gene and functional annotation (involved in) according to (TAIR, www.arabidopsis.org), Fold change (Log2) and corrected significance of the regulation (p adj.). The rightest column indicates differential expression ((Log2 fold change) at 27°C compared to 22°C. Green and red font indicates up and down regulation respectively. No number means no significant change.

| **Condition** | **AGI code** | **Gene product** | **Fold change** | **p adj.** | **Fold change 27C vs. 22C** |
| --- | --- | --- | --- | --- | --- |
| **22°C Up** | *AT4G33550* | Bifunctional inhibitor/lipid-transfer protein/seed storage 2S albumin superfamily protein;  involved in: lipid transport | **1.80** | 2.61E-05 | 1.43 |
|  | *AT5G03350* | *SAI-LLP1*; Legume lectin family protein;  involved in: cellular response to salicylic acid stimulus, phosphorylation, systemic acquired resistance | **1.73** | 0.02051 | 0.27 |
| **27°C Up** | *AT2G22810* | *ACS4*; key enzyme in the biosynthesis of ethylene. ACS4 is specifically induced by auxin;  involved in: 1-aminocyclopropane-1-carboxylate biosynthetic process, cellular response to iron ion, ethylene biosynthetic process, fruit ripening, response to auxin | **6.23** | 0.051023 | - |
|  | *AT1G15580* | *IAA5*; Auxin induced protein;  involved in: auxin-activated signalling pathway, regulation of transcription, response to auxin | **3.49** | 0.051023 | - |
|  | *AT2G37870* | Bifunctional inhibitor/lipid-transfer protein/seed storage 2S albumin superfamily protein;  involved in: lipid transport | **2.61** | 0.011843 | 11.03 |
|  | *AT2G14960* | *GH3.1*; encodes a protein similar to IAA-amido synthases;  involved in: response to auxin | **2.31** | 0.071511 | 0.66 |
|  | *AT3G23635* | *ROTUNDIFOLIA like 13* | **2.22** | 0.040451 | 9.45 |
|  | *AT4G33550* | Bifunctional inhibitor/lipid-transfer protein/seed storage 2S albumin superfamily protein;  involved in: lipid transport | **1.74** | 0.040451 | 1.43 |
|  | *AT2G23170* | *GH3.3*; encodes an IAA-amido synthase that conjugates amino acids to auxin;  involved in: auxin homeostasis, response to auxin | **1.67** | 0.000557 | 0.59 |
|  | *AT2G43050* | *ATPMEPCRD*; Plant invertase/pectin methylesterase inhibitor superfamily;  involved in: cell wall modification, pectin catabolic process | **1.64** | 0.007324 | 10.4 |
| **27°C Down** | *AT4G01700* | Chitinase family protein;  involved in: carbohydrate metabolic process, cell wall macromolecule catabolic process, chitin catabolic process, defense response to fungus | **0.49** | 0.040451 | 0.80 |
|  | *AT4G21680* | *NRT1.8*; Encodes a nitrate transporter (NRT1.8). Functions in nitrate removal from the xylem sap. Mediates cadmium tolerance;  involved in: nitrate assimilation, oligopeptide transport, response to cadmium ion, response to nitrate, transmembrane transport | **0.59** | 0.000597 | 3.18 |

**Table S7. Normalized RNA-seq read counts of genes differentially regulated by Heatin and high temperature in seven day-old seedlings.** Commonly upregulated (top part of table) and down regulated (lower part of table) genes, indicated by AGI codes and gene description, as indicated in the Venn diagrams depicted in Figure S9c,d. Values are colour-coded according to their relative expression compared with the other two samples. Blue, white and orange respectively correspond with lowest, middle and highest values. Summarized gene descriptions are retrieved from TAIR (www.arabidopsis.org).

| **Upregulated genes** | | | | |
| --- | --- | --- | --- | --- |
| **AGI code** | **Heatin 22°C** | **Heatin 27°C** | **DMSO 27°C** | **Gene description** |
| AT2G43050 | 747.2095 | 2267.798 | 706.0377 | Plant invertase/pectin methylesterase inhibitor superfamily (ATPMEPCRD) |
| AT1G65310 | 1109.476 | 1554.834 | 873.8306 | XYLOGLUCAN ENDOTRANSGLUCOSYLASE/HYDROLASE 17 (ATXTH17) |
| AT2G23170 | 849.7201 | 4468.59 | 809.5 | encodes an IAA-amido synthase that conjugates Asp and other amino acids to auxin in vitro. (GH3.3) |
| AT1G78970 | 941.7625 | 1199.811 | 545.1526 | LUPEOL SYNTHASE 1 (LUP1) |
| AT1G70830 | 6353.93 | 19925.13 | 10766.23 | MLP-LIKE PROTEIN 28 (MLP28) |
| AT1G27020 | 366.6233 | 1031.431 | 460.4124 | plant/protein |
| AT1G70890 | 1038.91 | 1937.954 | 1090.275 | MLP-LIKE PROTEIN 43 (MLP43);MAJOR LATEX PROTEIN LIKE 43 (MLP43) |
| AT3G15540 | 735.9883 | 1097.294 | 683.396 | INDOLE-3-ACETIC ACID INDUCIBLE 19 (IAA19);MASSUGU 2 (MSG2) |
| AT1G20620 | 48186.53 | 178894.6 | 117815.1 | Catalase, catalyses the breakdown of hydrogen peroxide (H2O2) into water and oxygen. CATALASE 3 (CAT3);SENESCENCE 2 (SEN2) |
| AT5G07010 | 3603.405 | 13510.22 | 9136.697 | Encodes a sulfotransferase that acts specifically on 11- and 12-hydroxyjasmonic acid. SULFOTRANSFERASE 2A (ST2A) |
| AT3G20470 | 1007.287 | 2877.741 | 1706.545 | GLYCINE-RICH PROTEIN 5 (GRP5) |
| AT4G08150 | 786.2683 | 1294.725 | 871.4705 | A member of class I knotted1-like homeobox gene family (together with KNAT2). KNOTTED-LIKE FROM ARABIDOPSIS THALIANA (KNAT1);BREVIPEDICELLUS 1 (BP1) |
| AT5G20710 | 143.0779 | 581.7532 | 233.0679 | BETA-GALACTOSIDASE 7 (BGAL7) |
| AT3G26170 | 75.21313 | 841.1999 | 538.6211 | putative cytochrome P450 (CYP71B19) |
| AT4G19420 | 876.0811 | 2720.572 | 1759.976 | Pectinacetylesterase family protein PECTIN ACETYLESTERASE 8 (PAE8) |
| AT2G18050 | 1358.086 | 5678.489 | 3638.176 | encodes a structurally divergent linker histone whose gene expression is induced by dehydration and ABA. HISTONE H1-3 (HIS1-3) |
| AT2G36220 | 729.9189 | 1966.355 | 1465.972 | hypothetical protein;(source:Araport11) protein_coding |
| AT5G19530 | 2271.289 | 4641.809 | 3445.932 | Encodes a spermine synthase. ACAULIS 5 (ACL5) |
| AT4G18970 | 1692.008 | 2506.265 | 1891.142 | GDSL-motif esterase/acyltransferase/lipase. Enzyme group with broad substrate specificity |
| AT2G43060 | 518.3309 | 1192.068 | 733.6907 | ILI1 BINDING BHLH 1 (IBH1) |
| AT3G28857 | 66.84184 | 110.8382 | 54.90018 | Encodes a atypical member of the bHLH family transcriptional factors. PACLOBUTRAZOL RESISTANCE 5 (PRE5) |
| AT5G37950 | 15.85963 | 44.55112 | 6.467209 | UDP-Glycosyltransferase superfamily protein |
| AT2G37130 | 5525.472 | 15467.37 | 7857.298 | Peroxidase superfamily protein;(source:Araport11) protein_coding |
| AT1G67810 | 360.3545 | 908.1091 | 492.7664 | Encodes a protein capable of stimulating the cysteine desulfurase activity of CpNifS (AT1G08490) in vitro. SULFUR E2 (SUFE2) |
| AT3G56980 | 280.0405 | 826.2258 | 256.365 | Encodes a member of the basic helix-loop-helix transcription factor protein. OBP3-RESPONSIVE GENE 3 (ORG3); (BHLH039) |
| AT1G64710 | 744.3602 | 1737.071 | 1055.361 | GroES-like zinc-binding alcohol dehydrogenase family protein |
| AT1G09480 | 108.8401 | 231.3696 | 144.3987 | similar to Eucalyptus gunnii alcohol dehydrogenase of unknown physiological function |
| AT3G25717 | 937.2998 | 1623.655 | 1194.445 | ROTUNDIFOLIA LIKE 16 (RTFL16);DEVIL 6 (DVL6) |
| AT5G04080 | 440.3337 | 873.3245 | 634.8393 | cysteine-rich TM module stress tolerance protein |
| AT5G03545 | 134.862 | 258.0086 | 154.5678 | Expressed in response to phosphate starvation, this response is enhanced by the presence of IAA. (AT4) INDUCED BY PI STARVATION 2 (ATIPS2); (AT4) |
| AT2G01670 | 1342.098 | 2225.146 | 1713.535 | NUDIX HYDROLASE HOMOLOG 17 (NUDT17) |

| **Downregulated genes** | | | | |
| --- | --- | --- | --- | --- |
| AT5G48000 | 48.95991 | 78.43973 | 148.5338 | Encodes a member of the CYP708A family of cytochrome P450 enzymes. THAH appears to add a hydroxyl group to the triterpene thalianol. "CYTOCHROME P450, FAMILY 708, SUBFAMILY A, POLYPEPTIDE 2" (CYP708A2);THALIANOL HYDROXYLASE 1 (THAH1) |
| AT5G20630 | 1076.235 | 829.6481 | 1160.156 | Encodes a germin-like protein GERMIN 3 (GER3) GERMIN-LIKE PROTEIN 3 (GLP3) |
| AT1G79840 | 261.5464 | 220.5388 | 285.2976 | Glabra 2, a homeodomain protein affects epidermal cell identity including trichomes, root hairs, and seed coat. GLABRA 2 (GL2) |
| AT2G33850 | 313.281 | 107.9272 | 191.1522 | E6-like protein |
| AT3G09220 | 1103.349 | 959.5911 | 1483.293 | putative laccase LACCASE 7 (LAC7) |
| AT4G28940 | 165.1746 | 68.07668 | 105.2761 | Phosphorylase superfamily protein |
| AT3G14210 | 2282.588 | 1445.559 | 1820.469 | A semidominant QTL which has an epistatic effect on the Epithiospecifier gene. Represses nitrile formation and favors isothiocyanate production during glucosinolate hydrolysis. EPITHIOSPECIFIER MODIFIER 1 (ESM1) |
| AT3G46700 | 167.2139 | 213.896 | 298.2333 | UDP-Glycosyltransferase superfamily protein |
| AT5G24140 | 32.98181 | 24.97136 | 53.47751 | Encodes a protein with similarity to squalene monoxygenases. SQUALENE MONOOXYGENASE 2 (SQP2) |
| AT1G66280 | 1892.105 | 1478.314 | 2207.717 | Glycosyl hydrolase superfamily protein (BGLU22) |
| AT5G62480 | 236.1034 | 226.3598 | 282.8259 | Encodes glutathione transferase belonging to the tau class of GSTs. GLUTATHIONE S-TRANSFERASE TAU 9 (GSTU9) ;GLUTATHIONE S-TRANSFERASE 14 (GST14) |
| AT5G48010 | 150.1205 | 165.9923 | 256.1203 | Encodes an oxidosqualene cyclase involved in the biosynthesis of thalianol, a tricyclic triterpenoid of unknown function. THALIANOL SYNTHASE 1 (THAS1) |
| AT3G20370 | 1876.806 | 885.1374 | 1104.548 | TRAF-like family protein |
| AT2G29750 | 390.5076 | 246.6302 | 385.7497 | UDP-glucosyl transferase 71C1; UDP-GLUCOSYL TRANSFERASE 71C1 (UGT71C1) |
| AT1G24020 | 178.7816 | 25.90262 | 53.24399 | MLP-LIKE PROTEIN 423 (MLP423) |
| AT4G33610 | 876.7477 | 481.3765 | 609.2147 | glycine-rich protein |
| AT4G15160 | 1018.81 | 938.0513 | 1330.269 | Bifunctional inhibitor/lipid-transfer protein/seed storage 2S albumin superfamily protein |
| AT1G12040 | 77.1701 | 7.908191 | 29.22345 | encodes a a chimeric leucine-rich repeat/extensin protein that regulates root hair morphogenesis and elongation. LEUCINE-RICH REPEAT/EXTENSIN 1 (LRX1) |
| AT5G10230 | 104.8928 | 23.26215 | 43.80854 | Encodes a calcium-binding protein annexin ANNEXIN 7 (ANN7) |
| AT4G00700 | 356.6288 | 299.0846 | 438.2514 | C2 calcium/lipid-binding plant phosphoribosyltransferase family protein |
| AT2G43150 | 9211.727 | 8695.316 | 9916.654 | Proline-rich extensin-like family protein |

**Table S8. GO-terms of biological processes significantly enriched amongst Heatin regulated genes after seven days.** Shown are results for the two tested temperature conditions (22°C; left, 27°C; right) for upregulated (UP) and downregulated (DN) genes, by GO accession identifier, the term description and the FDR-corrected p-values.

| **Condition** | **GO accession** | **Term** | **FDR p-value** |  |  | **Condition** | **GO accession** | **Term** | **FDR p-value** |
| --- | --- | --- | --- | --- | --- | --- | --- | --- | --- |
| **22-UP** | GO:0050896 | response to stimulus | 5.70E-05 |  |  |  | GO:0050896 | response to stimulus | 2.20E-09 |
|  | GO:0042221 | response to chemical stimulus | 0.00026 |  |  | **27-UP** | GO:0042221 | response to chemical stimulus | 5.60E-07 |
|  | GO:0009733 | response to auxin stimulus | 0.0014 |  |  |  | GO:0006979 | response to oxidative stress | 2.20E-05 |
|  | GO:0009719 | response to endogenous stimulus | 0.0017 |  |  |  | GO:0009607 | response to biotic stimulus | 3.70E-05 |
|  | GO:0010033 | response to organic substance | 0.0031 |  |  |  | GO:0055072 | iron ion homeostasis | 4.00E-05 |
|  | GO:0009725 | response to hormone stimulus | 0.014 |  |  |  | GO:0051707 | response to other organism | 4.00E-05 |
|  | GO:0007017 | microtubule-based process | 0.037 |  |  |  | GO:0065008 | regulation of biological quality | 4.00E-05 |
| **22-DN** | GO:0010876 | lipid localization | 1.30E-09 |  |  |  | GO:0006826 | iron ion transport | 4.60E-05 |
|  | GO:0006629 | lipid metabolic process | 0.00029 |  |  |  | GO:0006950 | response to stress | 5.40E-05 |
|  | GO:0006869 | lipid transport | 0.00035 |  |  |  | GO:0009617 | response to bacterium | 6.40E-05 |
|  | GO:0009409 | response to cold | 0.00086 |  |  |  | GO:0000041 | transition metal ion transport | 6.50E-05 |
|  | GO:0050896 | response to stimulus | 0.0012 |  |  |  | GO:0048878 | chemical homeostasis | 0.00012 |
|  | GO:0044255 | cellular lipid metabolic process | 0.0012 |  |  |  | GO:0015674 | di-, tri-valent inorganic cation transport | 0.00012 |
|  | GO:0019748 | secondary metabolic process | 0.0027 |  |  |  | GO:0050801 | ion homeostasis | 0.00013 |
|  | GO:0044275 | cellular carbohydrate catabolic process | 0.0029 |  |  |  | GO:0042545 | cell wall modification | 0.00042 |
|  | GO:0016052 | carbohydrate catabolic process | 0.0029 |  |  |  | GO:0051704 | multi-organism process | 0.00062 |
|  | GO:0009628 | response to abiotic stimulus | 0.0064 |  |  |  | GO:0010035 | response to inorganic substance | 0.00073 |
|  | GO:0006631 | fatty acid metabolic process | 0.0069 |  |  |  | GO:0009628 | response to abiotic stimulus | 0.0014 |
|  | GO:0009266 | response to temperature stimulus | 0.0071 |  |  |  | GO:0009664 | plant-type cell wall organization | 0.0021 |
|  | GO:0042221 | response to chemical stimulus | 0.0094 |  |  |  | GO:0042592 | homeostatic process | 0.0024 |
|  | GO:0048869 | cellular developmental process | 0.01 |  |  |  | GO:0009827 | plant-type cell wall modification | 0.0024 |
|  | GO:0009607 | response to biotic stimulus | 0.012 |  |  |  | GO:0000302 | response to reactive oxygen species | 0.0026 |
|  | GO:0006950 | response to stress | 0.014 |  |  |  | GO:0055080 | cation homeostasis | 0.0034 |
|  | GO:0051707 | response to other organism | 0.025 |  |  |  | GO:0006873 | cellular ion homeostasis | 0.0045 |
|  | GO:0032787 | monocarboxylic acid metabolic process | 0.026 |  |  |  | GO:0055082 | cellular chemical homeostasis | 0.0046 |
|  | GO:0009414 | response to water deprivation | 0.026 |  |  |  | GO:0009605 | response to external stimulus | 0.0049 |
|  | GO:0009555 | pollen development | 0.026 |  |  |  | GO:0006576 | cellular biogenic amine metabolic process | 0.0049 |
|  | GO:0005976 | polysaccharide metabolic process | 0.026 |  |  |  | GO:0055066 | di-, tri-valent inorganic cation homeostasis | 0.0058 |
|  | GO:0044262 | cellular carbohydrate metabolic process | 0.027 |  |  |  | GO:0009719 | response to endogenous stimulus | 0.0074 |
|  | GO:0044248 | cellular catabolic process | 0.028 |  |  |  | GO:0009266 | response to temperature stimulus | 0.012 |
|  | GO:0009415 | response to water | 0.028 |  |  |  | GO:0042742 | defense response to bacterium | 0.014 |
|  | GO:0006720 | isoprenoid metabolic process | 0.032 |  |  |  | GO:0009733 | response to auxin stimulus | 0.015 |
|  | GO:0008610 | lipid biosynthetic process | 0.032 |  |  |  | GO:0010038 | response to metal ion | 0.015 |
|  | GO:0022622 | root system development | 0.033 |  |  |  | GO:0030003 | cellular cation homeostasis | 0.015 |
|  | GO:0048364 | root development | 0.033 |  |  |  | GO:0048589 | developmental growth | 0.016 |
|  | GO:0033036 | macromolecule localization | 0.039 |  |  |  | GO:0060560 | developmental growth involved in morphogenesis | 0.016 |
|  | GO:0009737 | response to abscisic acid stimulus | 0.049 |  |  |  | GO:0009826 | unidimensional cell growth | 0.016 |
|  | GO:0048513 | organ development | 0.049 |  |  |  | GO:0010033 | response to organic substance | 0.017 |
|  | GO:0048731 | system development | 0.049 |  |  |  | GO:0048869 | cellular developmental process | 0.017 |
|  |  |  |  |  |  |  | GO:0000902 | cell morphogenesis | 0.022 |
|  |  |  |  |  |  |  | GO:0032502 | developmental process | 0.034 |
|  |  |  |  |  |  |  | GO:0009725 | response to hormone stimulus | 0.034 |
|  |  |  |  |  |  |  | GO:0009408 | response to heat | 0.034 |
|  |  |  |  |  |  |  | GO:0006812 | cation transport | 0.035 |
|  |  |  |  |  |  |  | GO:0006811 | ion transport | 0.035 |
|  |  |  |  |  |  |  | GO:0030001 | metal ion transport | 0.035 |
|  |  |  |  |  |  |  | GO:0032989 | cellular component morphogenesis | 0.035 |
|  |  |  |  |  |  |  | GO:0051179 | localization | 0.035 |
|  |  |  |  |  |  |  | GO:0006810 | transport | 0.037 |
|  |  |  |  |  |  |  | GO:0051234 | establishment of localization | 0.038 |
|  |  |  |  |  |  |  | GO:0042398 | cellular amino acid derivative biosynth. process | 0.04 |
|  |  |  |  |  |  |  | GO:0019725 | cellular homeostasis | 0.041 |
|  |  |  |  |  |  |  | GO:0010876 | lipid localization | 7.80E-08 |
|  |  |  |  |  |  | **27-DN** | GO:0048316 | seed development | 0.00048 |
|  |  |  |  |  |  |  | GO:0006722 | triterpenoid metabolic process | 0.00048 |
|  |  |  |  |  |  |  | GO:0010154 | fruit development | 0.00069 |
|  |  |  |  |  |  |  | GO:0009791 | post-embryonic development | 0.003 |
|  |  |  |  |  |  |  | GO:0009790 | embryonic development | 0.0042 |
|  |  |  |  |  |  |  | GO:0009793 | embryonic develop. ending in seed dormancy | 0.012 |
|  |  |  |  |  |  |  | GO:0019748 | secondary metabolic process | 0.017 |
|  |  |  |  |  |  |  | GO:0015833 | peptide transport | 0.017 |
|  |  |  |  |  |  |  | GO:0006857 | oligopeptide transport | 0.017 |
|  |  |  |  |  |  |  | GO:0032502 | developmental process | 0.032 |
|  |  |  |  |  |  |  | GO:0006950 | response to stress | 0.033 |
|  |  |  |  |  |  |  | GO:0009415 | response to water | 0.04 |
|  |  |  |  |  |  |  | GO:0007275 | multicellular organismal development | 0.044 |
|  |  |  |  |  |  |  | GO:0051179 | localization | 0.044 |

**Table S9. Change in expression (Log2) of selected auxin biosynthesis, perception and signalling genes** (AUX/IAA, Auxin response factors (ARFs) and Small Auxin UP RNA (SAURs), in response to Heatin treatment compared to DMSO mock, in 2 day-old (2d) and 7 day-old (7d) Col-0 wild type seedlings, at 22^o^C and 27^o^C. Indicated are: Gene ID, Gene name, Arabidopsis Genome Initiative (AGI) identifier (www.arabidopsis.org). Positive values with blue shading, and negative values with red shading under the heading: ‘*Heatin vs. DMSO’*, indicate up and downregulation respectively. Significance of the transcriptional regulation is indicated under the heading ‘*p values’*, where significant values (P< 0.05) are indicated in red letters. Missing values indicates that no reads corresponding to the gene were detected.

**Table S10. Gene identifiers of proteins significantly enriched in the ‘Elute’ fraction.** Shown are Arabidopsis Gene Identifier (AGI) codes, -LOG p-value and the log2-fold change (difference) of protein groups significantly enriched in the Heatin-eluted fraction compared to the ‘On-bead’ fraction and the gene symbol and name, if available (www.arabidopsis.org). Note that in cases that where multiple proteins were assigned to a protein group, all corresponding AGI codes are presented within the same boxed row.

| **Gene Model** | **-LOG (p-value)** | **Difference** | **Symbol** | **Name** |
| --- | --- | --- | --- | --- |
| AT1G02305.1 | 3.468 | 2.744 | ATCATHB2 |  |
| AT1G03880.1 | 3.344 | 2.798 | CRU2 | CRUCIFERIN 2 |
| AT1G03890.1 | 2.755 | 1.463 |  |  |
| AT1G04410.1 | 3.673 | 1.716 | c-NAD-MDH1 | CYTOSOLIC-NAD-DEPENDENT MALATE DEHYDROGENASE 1 |
| AT1G04750.1 | 2.644 | 0.967 | VAMP721 | VESICLE-ASSOCIATED MEMBRANE PROTEIN 721 |
| AT2G33120.2 |  |  | SAR1 | SYNAPTOBREVIN-RELATED PROTEIN 1 |
| AT1G05510.1 | 2.049 | 1.456 | OBAP1A | OIL BODY-ASSOCIATED PROTEIN 1A |
| AT1G06680.1 | 1.705 | 1.550 | PSBP-1 | PHOTOSYSTEM II SUBUNIT P-1 |
| AT1G06690.1 | 3.779 | 4.223 |  |  |
| AT1G09310.1 | 2.062 | 1.213 |  |  |
| AT1G10840.1 | 1.012 | 0.928 | TIF3H1 | TRANSLATION INITIATION FACTOR 3 SUBUNIT H1 |
| AT1G11430.1 | 1.122 | 1.894 | MORF9 | MULTIPLE ORGANELLAR RNA EDITING FACTOR 9 |
| AT1G11580.1 | 1.520 | 2.735 | PMEPCRA | METHYLESTERASE PCR A |
| AT1G11650.2 | 0.987 | 1.155 | RBP45B |  |
| AT1G11910.1 | 5.961 | 5.066 | APA1 | ASPARTIC PROTEINASE A1 |
| AT1G12840.1 | 1.091 | 1.989 | DET3 | DE-ETIOLATED 3 |
| AT1G13080.1 | 2.081 | 2.479 | CYP71B2 | CYTOCHROME P450, FAMILY 71, SUBFAMILY B, POLYPEPTIDE 2 |
| AT1G13270.1 | 1.889 | 1.247 | MAP1C | METHIONINE AMINOPEPTIDASE 1B |
| AT1G17720.1 | 5.749 | 2.684 | ATBBETA |  |
| AT1G21380.1 | 1.628 | 0.716 |  |  |
| AT1G76970.1 |  |  |  |  |
| AT1G22270.1 | 1.436 | 1.721 | TRM112B | TRNA METHYLTRANSFERASE 112B |
| AT1G26850.1 | 2.676 | 3.049 |  |  |
| AT1G27450.3 | 0.929 | 2.237 | APT1 | ADENINE PHOSPHORIBOSYL TRANSFERASE 1 |
| AT1G29470.1 | 0.996 | 0.502 |  |  |
| AT1G32200.1 | 1.774 | 2.988 | ATS1 |  |
| AT1G32990.1 | 1.043 | 0.923 | PRPL11 | PLASTID RIBOSOMAL PROTEIN L11 |
| AT1G36310.1 | 2.789 | 1.475 | TRM9 | TRNA METHYLTRANSFERASE 9 |
| AT1G42960.1 | 3.820 | 2.378 |  |  |
| AT1G44170.1 | 2.736 | 2.112 | ALDH3H1 | ALDEHYDE DEHYDROGENASE 3H1 |
| AT1G44575.1 | 0.793 | 1.044 | NPQ4 | NONPHOTOCHEMICAL QUENCHING 4 |
| AT1G47128.1 | 3.663 | 3.389 | RD21A | RESPONSIVE TO DEHYDRATION 21A |
| AT1G50900.1 | 2.800 | 2.019 | GDC1 | GRANA DEFICIENT CHLOROPLAST 1 |
| AT1G52380.1 | 1.978 | 1.691 |  |  |
| AT1G54870.1 | 3.598 | 1.909 |  |  |
| AT1G55480.1 | 2.511 | 0.946 | ZKT | PROTEIN CONTAINING PDZ DOMAIN, A K-BOX DOMAIN, AND A TPR REGION |
| AT1G56070.1 | 6.372 | 2.270 | LOS1 | LOW EXPRESSION OF OSMOTICALLY RESPONSIVE GENES 1 |
| AT1G56590.1 | 3.425 | 7.587 | ZIP4 | ZIG SUPPRESSOR 4 |
| AT1G57720.1 | 3.816 | 2.555 |  |  |
| AT1G60680.1 | 3.294 | 1.067 | AGD2 | ARF-GAP DOMAIN 2 |
| AT1G60710.1 | 1.977 | 2.968 | ATB2 |  |
| AT1G60730.3 |  |  |  |  |
| AT1G61790.1 | 1.859 | 0.927 | OST3/6 | OLIGOSACCHARYLTRANSFERASE SUBUNIT 3/6 |
| AT1G62290.1 | 4.900 | 6.700 | PASPA2 | PUTATIVE ASPARTIC PROTEINASE A2 |
| AT1G62780.1 | 2.325 | 2.763 |  |  |
| AT1G63610.2 | 1.368 | 1.215 |  |  |
| AT1G65980.1 | 1.595 | 0.816 | TPX1 | THIOREDOXIN-DEPENDENT PEROXIDASE 1 |
| AT1G67090.1 | 4.753 | 4.958 | RBCS1A | RIBULOSE BISPHOSPHATE CARBOXYLASE SMALL CHAIN 1A |
| AT1G69740.1 | 2.320 | 2.417 | HEMB1 |  |
| AT1G75950.1 | 3.525 | 2.244 | SKP1 | S PHASE KINASE-ASSOCIATED PROTEIN 1 |
| AT1G76080.1 | 3.375 | 3.275 | CDSP32 | CHLOROPLASTIC DROUGHT-INDUCED STRESS PROTEIN OF 32 KD |
| AT1G76680.2 | 1.131 | 0.789 | OPR1 | 12-OXOPHYTODIENOATE REDUCTASE 1 |
| AT1G77060.1 | 3.271 | 0.794 |  |  |
| AT1G78900.1 | 1.961 | 0.950 | VHA-A | VACUOLAR ATP SYNTHASE SUBUNIT A |
| AT1G79550.1 | 1.396 | 0.684 | PGK | PHOSPHOGLYCERATE KINASE |
| AT2G01470.1 | 4.056 | 3.164 | STL2P | SEC12P-LIKE 2 PROTEIN |
| AT2G05830.1 | 1.156 | 1.124 | MTI1 | 5-METHYLTHIORIBOSE KINASE 1 |
| AT2G06850.1 | 2.676 | 2.296 | XTH4 | XYLOGLUCAN ENDOTRANSGLUCOSYLASE/HYDROLASE 4 |
| AT2G07698.1 | 1.123 | 0.550 |  |  |
| AT2G14720.1 | 3.382 | 1.576 | VSR4 | VACUOLAR SORTING RECEPTOR 4 (VSR4) |
| AT2G14740.1 |  |  | VSR3 | VACULOLAR SORTING RECEPTOR 3 |
| AT2G19860.1 | 4.596 | 2.754 | HXK2 | HEXOKINASE 2 |
| AT2G21270.3 | 2.137 | 1.546 | UFD1 | UBIQUITIN FUSION DEGRADATION 1 |
| AT2G22475.1 | 2.197 | 2.224 | GEM | GL2-EXPRESSION MODULATOR |
| AT2G22780.1 | 2.540 | 0.930 | PMDH1 | PEROXISOMAL NAD-MALATE DEHYDROGENASE 1 |
| AT2G25070.1 | 3.280 | 1.185 |  |  |
| AT2G25970.1 | 1.707 | 1.714 |  |  |
| AT2G27710.1 | 2.394 | 1.375 |  |  |
| AT2G27720.2 | 1.887 | 1.837 |  |  |
| AT2G28000.1 | 2.539 | 0.984 | CPN60A | CHAPERONIN-60ALPHA |
| AT2G28490.1 | 5.634 | 4.497 |  |  |
| AT2G29450.1 | 1.496 | 2.069 | GSTU5 | GLUTATHIONE S-TRANSFERASE TAU 5 |
| AT2G35370.1 | 3.501 | 2.093 | GDCH | GLYCINE DECARBOXYLASE COMPLEX H |
| AT2G35410.1 | 3.483 | 2.780 |  |  |
| AT2G36530.1 | 3.338 | 1.311 | LOS2 | LOW EXPRESSION OF OSMOTICALLY RESPONSIVE GENES 2 |
| AT2G36580.1 | 3.979 | 2.980 |  |  |
| AT2G37220.1 | 4.970 | 2.351 |  |  |
| AT2G38560.1 | 1.764 | 1.210 | TFIIS | TRANSCRIPT ELONGATION FACTOR IIS |
| AT2G39080.1 | 2.783 | 2.146 | EMB2799 | EMBRYO DEFECTIVE 2799 |
| AT2G43750.1 | 1.740 | 1.113 | OASB | O-ACETYLSERINE (THIOL) LYASE B |
| AT2G43910.2 | 3.753 | 1.425 | HOL1 | HARMLESS TO OZONE LAYER 1 |
| AT2G44060.1 | 3.301 | 1.738 | LEA26 | LATE EMBRYOGENESIS ABUNDANT 26 (LEA26) |
| AT2G44350.2 | 5.287 | 2.857 | ATCS |  |
| AT2G44610.1 | 1.849 | 1.571 | RAB6A |  |
| AT2G44650.1 | 1.996 | 1.820 | CHL-CPN10 | CHLOROPLAST CHAPERONIN 10 |
| AT2G47940.1 | 0.987 | 1.772 | DEG2 | DEGRADATION OF PERIPLASMIC PROTEINS 2 |
| AT3G01420.1 | 3.820 | 4.946 | DOX1 |  |
| AT3G01500.2 | 2.751 | 1.498 | CA1 | CARBONIC ANHYDRASE 1 |
| AT3G01520.1 | 2.884 | 1.639 |  |  |
| AT3G06300.1 | 4.341 | 2.468 | P4H2 | PROLYL 4-HYDROXYLASE 2 |
| AT3G06860.1 | 2.649 | 1.619 | MFP2 | MULTIFUNCTIONAL PROTEIN 2 |
| AT3G07880.1 | 1.822 | 1.871 | SCN1 | SUPERCENTIPEDE1 |
| AT3G08740.1 | 1.351 | 1.436 |  |  |
| AT3G08940.2 | 2.811 | 2.411 | LHCB4.2 | LIGHT HARVESTING COMPLEX PHOTOSYSTEM II |
| AT3G09260.1 | 1.966 | 1.642 | PYK10 |  |
| AT3G09820.1 | 3.284 | 1.420 | ADK1 | ADENOSINE KINASE 1 |
| AT5G03300.1 |  |  | ADK2 | ADENOSINE KINASE 2 |
| AT3G10670.1 | 3.235 | 2.435 | NAP7 | NON-INTRINSIC ABC PROTEIN 7 |
| AT3G11630.1 | 4.893 | 4.987 | 2CPA | 2-CYS PEROXIREDOXIN A |
| AT3G11780.2 | 1.104 | 1.212 |  |  |
| AT3G12670.1 | 1.717 | 1.484 | emb2742 | EMBRYO DEFECTIVE 2742 |
| AT3G13235.1 | 2.936 | 0.925 | DDI1 | DNA-DAMAGE INDUCIBLE 1 |
| AT3G15260.1 | 1.326 | 0.577 |  |  |
| AT3G15356.1 | 3.643 | 4.040 |  |  |
| AT3G15730.1 | 2.868 | 2.640 | PLDALPHA1 | PHOSPHOLIPASE D ALPHA 1 |
| AT3G16370.1 | 1.335 | 1.116 |  |  |
| AT3G16420.1 | 2.761 | 0.988 | PBP1 | PYK10-BINDING PROTEIN 1 |
| AT3G16450.1 | 0.784 | 1.094 | JAL33 | JACALIN-RELATED LECTIN 33 |
| AT3G16640.1 | 3.095 | 1.675 | TCTP1 | TRANSLATIONALLY CONTROLLED TUMOR PROTEIN |
| AT3G21190.1 | 2.680 | 0.804 | MSR1 | MANNAN SYNTHESIS RELATED 1 |
| AT3G22640.1 | 2.427 | 1.627 | PAP85 |  |
| AT3G23400.1 | 5.203 | 5.369 | FIB4 | FIBRILLIN 4 |
| AT3G26060.2 | 1.755 | 1.403 | PRXQ | PEROXIREDOXIN Q |
| AT3G26070.1 | 2.121 | 1.290 |  |  |
| AT3G27740.1 | 3.302 | 2.765 | CARA | CARBAMOYL PHOSPHATE SYNTHETASE A |
| AT3G27850.1 | 2.267 | 2.315 | RPL12-C | RIBOSOMAL PROTEIN L12-C (RPL12-C) |
| AT3G27830.1 |  |  | RPL12-A | RIBOSOMAL PROTEIN L12-A (RPL12-A) |
| AT3G29360.1 | 1.811 | 1.668 | UGD2 | UDP-GLUCOSE DEHYDROGENASE 2 |
| AT5G15490.1 |  |  | UGD3 | UDP-GLUCOSE DEHYDROGENASE 3 |
| AT3G44300.1 | 0.886 | 0.984 | NIT2 | NITRILASE 2 |
| AT3G44310.1 | 3.114 | 1.739 | NIT1 | NITRILASE 1 |
| AT3G44320.1 | 1.581 | 1.746 | NIT3 | NITRILASE 3 |
| AT3G44890.1 | 1.187 | 0.677 | RPL9 | RIBOSOMAL PROTEIN L9 |
| AT3G46740.1 | 1.377 | 1.778 | TOC75-III | TRANSLOCON AT THE OUTER ENVELOPE MEMBRANE OF CHLOROPLASTS 75-III |
| AT3G47520.1 | 1.434 | 0.365 | MDH | MALATE DEHYDROGENASE |
| AT3G48410.1 | 2.574 | 0.881 |  |  |
| AT3G51020.1 | 3.008 | 5.916 |  |  |
| AT3G52150.1 | 1.031 | 0.870 | PSRP2 | PLASTID-SPECI&#64257;C RIBOSOMAL PROTEIN 2 |
| AT3G52960.1 | 2.885 | 1.719 | PRXIIE | PEROXIREDOXIN-II-E |
| AT3G52990.1 | 4.023 | 1.739 |  |  |
| AT3G55250.1 | 2.231 | 1.442 | PDE329 | PIGMENT DEFECTIVE 329 |
| AT3G55440.1 | 2.378 | 1.422 | TPI | TRIOSEPHOSPHATE ISOMERASE |
| AT3G56490.1 | 1.141 | 1.457 | HIT3 | HIS TRIAD FAMILY PROTEIN 3 |
| AT3G57010.1 | 1.220 | 0.789 |  |  |
| AT3G58450.1 | 2.711 | 2.693 | USP | UNIVERSAL STRESS PROTEIN |
| AT3G59990.1 | 2.714 | 3.104 | MAP2B | METHIONINE AMINOPEPTIDASE 2B |
| AT3G61470.1 | 1.948 | 1.262 | LHCA2 | PHOTOSYSTEM I LIGHT HARVESTING COMPLEX GENE 2 |
| AT3G62290.1 | 2.895 | 2.618 | ARFA1E | ADP-RIBOSYLATION FACTOR A1E |
| AT2G47170.1 |  |  | ARF1A1C |  |
| AT1G70490.1 |  |  | ARFA1D |  |
| AT1G23490.1 |  |  | ARF1 | ADP-RIBOSYLATION FACTOR 1 |
| AT1G10630.1 |  |  | ARFA1F | ADP-RIBOSYLATION FACTOR A1F |
| AT5G14670.1 |  |  | ARFA1B | ADP-RIBOSYLATION FACTOR A1B |
| AT3G63190.1 | 3.400 | 1.485 | RRF | RIBOSOME RECYCLING FACTOR, CHLOROPLAST PRECURSOR |
| AT4G01480.1 | 1.712 | 2.307 | PPa5 | PYROPHOSPHORYLASE 5 |
| AT1G01050.1 |  |  |  |  |
| AT4G01900.1 | 4.496 | 4.857 | GLB1 | GLNB1 HOMOLOG |
| AT4G04460.1 | 3.531 | 3.769 | PASPA3 | PUTATIVE ASPARTIC PROTEINASE A3 |
| AT4G05160.1 | 2.517 | 2.453 |  |  |
| AT4G09320.1 | 1.349 | 2.069 | NDPK1 |  |
| AT4G11010.1 | 4.412 | 2.890 | NDPK3 | NUCLEOSIDE DIPHOSPHATE KINASE 3 |
| AT4G23900.1 |  |  |  |  |
| AT4G11150.1 | 1.104 | 0.598 | TUF | VACUOLAR ATP SYNTHASE SUBUNIT E1 |
| AT1G64200.1 |  |  | VHA-E3 | VACUOLAR H+-ATPASE SUBUNIT E ISOFORM 3 |
| AT4G12060.1 | 2.386 | 2.231 | CLPT2 |  |
| AT4G12800.1 | 1.285 | 1.751 | PSAL | PHOTOSYSTEM I SUBUNIT L |
| AT4G14890.1 | 1.528 | 1.803 | FdC1 | FERREDOXIN C 1 |
| AT4G16210.1 | 3.152 | 4.380 | ECHIA | ENOYL-COA HYDRATASE/ISOMERASE A |
| AT4G17600.1 | 1.296 | 1.388 | LIL3:1 | LIGHT-HARVESTING-LIKE 3:1 |
| AT4G18090.1 | 3.750 | 3.993 |  |  |
| AT4G20360.1 | 1.308 | 0.438 | ATRABE1B | RAB GTPASE HOMOLOG E1B |
| AT4G25080.3 | 2.898 | 1.642 | CHLM | MAGNESIUM-PROTOPORPHYRIN IX METHYLTRANSFERASE |
| AT4G25130.1 | 2.170 | 2.926 | PMSR4 | PEPTIDE MET SULFOXIDE REDUCTASE 4 |
| AT4G28520.1 | 2.257 | 2.566 | CRU3 | CRUCIFERIN 3 |
| AT4G29130.1 | 4.630 | 4.652 | HXK1 | HEXOKINASE 1 |
| AT4G29510.1 | 1.033 | 0.692 | PRMT11 | ARGININE METHYLTRANSFERASE 11 |
| AT4G31530.2 | 1.371 | 1.145 |  |  |
| AT4G31880.1 | 1.220 | 0.619 | PDS5C |  |
| AT4G34910.1 | 1.087 | 0.492 |  |  |
| AT4G35090.1 | 1.090 | 0.794 | CAT2 | CATALASE 2 |
| AT4G36250.1 | 1.746 | 0.748 | ALDH3F1 | ALDEHYDE DEHYDROGENASE 3F1 (ALDH3F1) |
| AT4G36700.1 | 1.122 | 1.025 |  |  |
| AT4G37040.1 | 2.443 | 3.337 | MAP1D | METHIONINE AMINOPEPTIDASE 1D |
| AT4G37070.2 | 4.137 | 4.993 | PLP1 |  |
| AT4G38510.5 | 4.098 | 3.840 | VAB2 | V-ATPASE B SUBUNIT 2 |
| AT4G38740.1 | 1.926 | 1.268 | ROC1 | ROTAMASE CYP 1 |
| AT5G01650.2 | 1.599 | 2.134 |  |  |
| AT5G01750.2 | 2.332 | 2.256 |  |  |
| AT5G04740.1 | 5.777 | 3.543 | ACR12 | ACT DOMAIN REPEATS 12 |
| AT5G09590.1 | 4.459 | 2.021 | MTHSC70-2 | MITOCHONDRIAL HSO70 2 |
| AT5G11170.1 | 1.675 | 1.017 | UAP56A | HOMOLOG OF HUMAN UAP56 A |
| AT5G11200.2 |  |  | UAP56B | HOMOLOG OF HUMAN UAP56 B |
| AT5G11450.1 | 3.791 | 2.963 | PPD5 | PSBP DOMAIN PROTEIN 5 |
| AT5G11500.1 | 2.726 | 3.282 |  |  |
| AT5G13410.1 | 3.616 | 2.325 |  |  |
| AT5G13710.1 | 2.921 | 1.482 | SMT1 | STEROL METHYLTRANSFERASE 1 |
| AT5G14030.1 | 2.544 | 3.738 |  |  |
| AT5G14660.1 | 2.244 | 1.482 | PDF1B | PEPTIDE DEFORMYLASE 1B |
| AT5G14740.1 | 2.996 | 1.431 | CA2 | CARBONIC ANHYDRASE 2 |
| AT5G15090.1 | 1.013 | 0.645 | VDAC3 | VOLTAGE DEPENDENT ANION CHANNEL 3 |
| AT5G15450.1 | 1.463 | 1.628 | CLPB3 | CASEIN LYTIC PROTEINASE B3 |
| AT5G16620.1 | 2.621 | 1.676 | TIC40 | TRANSLOCON AT THE INNER ENVELOPE MEMBRANE OF CHLOROPLASTS 40 |
| AT5G17770.1 | 2.676 | 3.816 | CBR | NADH:CYTOCHROME B5 REDUCTASE 1 |
| AT5G19140.1 | 4.069 | 1.328 | AILP1 |  |
| AT5G19940.1 | 3.206 | 1.301 |  |  |
| AT5G20720.1 | 1.795 | 1.287 | CPN20 | CHAPERONIN 20 |
| AT5G20890.1 | 2.769 | 0.953 |  |  |
| AT5G22340.2 | 1.725 | 3.319 |  |  |
| AT5G23300.1 | 1.941 | 2.591 | PYRD | PYRIMIDINE D |
| AT5G28500.1 | 1.474 | 1.162 |  |  |
| AT5G28750.1 | 1.106 | 2.682 |  |  |
| AT5G35630.1 | 2.269 | 1.059 | GS2 | GLUTAMINE SYNTHETASE 2 |
| AT5G37510.2 | 3.826 | 2.615 | EMB1467 | EMBRYO DEFECTIVE 1467 |
| AT5G38420.1 | 4.391 | 3.888 | RBCS2B | RUBISCO SMALL SUBUNIT 2B |
| AT5G38410.3 |  |  | RBCS3B | RUBISCO SMALL SUBUNIT 3B (RBCS3B) |
| AT5G38660.1 | 3.491 | 2.942 | APE1 | ACCLIMATION OF PHOTOSYNTHESIS TO ENVIRONMENT |
| AT5G43060.1 | 2.866 | 1.181 | RD21B | ESPONSIVE TO DEHYDRATION 21B |
| AT5G43830.1 | 1.562 | 0.776 |  |  |
| AT5G44120.3 | 1.666 | 1.114 | CRA1 | CRUCIFERINA |
| AT5G45690.1 | 1.272 | 0.415 |  |  |
| AT5G48230.2 | 2.448 | 1.578 | ACAT2 | ACETOACETYL-COA THIOLASE 2 |
| AT5G48300.1 | 1.567 | 0.530 | ADG1 | ADP GLUCOSE PYROPHOSPHORYLASE 1 |
| AT5G48580.1 | 3.158 | 2.389 | FKBP15-2 | FK506- AND RAPAMYCIN-BINDING PROTEIN 15 KD-2 |
| AT5G48810.1 | 1.822 | 1.708 | CB5-D | CYTOCHROME B5 ISOFORM D |
| AT5G53490.3 | 2.816 | 1.804 | TL17 | THYLAKOID LUMENAL 17.4 KDA PROTEIN |
| AT5G53560.1 | 2.633 | 2.576 | CB5-E | CYTOCHROME B5 ISOFORM E |
| AT5G57490.1 | 1.697 | 1.856 | VDAC4 | VOLTAGE DEPENDENT ANION CHANNEL 4 |
| AT5G57655.2 | 0.921 | 0.936 |  |  |
| AT5G58060.2 | 0.884 | 1.012 | YKT61 |  |
| AT5G58180.1 |  |  | ATYKT62 |  |
| AT5G58070.1 | 2.512 | 3.325 | TIL | TEMPERATURE-INDUCED LIPOCALIN |
| AT5G58250.1 | 2.027 | 3.920 | EMB3143 | EMBRYO DEFECTIVE 3143 |
| AT5G59160.1 | 2.311 | 1.657 | TOPP2 | TYPE ONE SERINE/THREONINE PROTEIN PHOSPHATASE 2 |
| AT3G46820.1 |  |  | TOPP5 | TYPE ONE SERINE/THREONINE PROTEIN PHOSPHATASE 5 |
| AT5G62670.1 | 0.988 | 0.854 | HA11 | H(+)-ATPASE 11 |
| AT1G80660.1 |  |  | HA9 | H(+)-ATPASE 9 |
| AT5G64300.1 | 2.868 | 2.149 | GCH | GTP CYCLOHYDROLASE II |
| ATCG00020.1 | 2.264 | 1.754 | PSBA | PHOTOSYSTEM II REACTION CENTER PROTEIN A |
| ATCG00120.1 | 0.843 | 0.857 | ATPA | ATP SYNTHASE SUBUNIT ALPHA |
| ATCG00470.1 | 3.654 | 2.427 | ATPE | ATP SYNTHASE EPSILON CHAIN |
| ATCG00490.1 | 2.626 | 1.656 | RBCL |  |
| ATCG00540.1 | 1.028 | 1.118 | PETA | PHOTOSYNTHETIC ELECTRON TRANSFER A |

**Table S11. GO-term enrichment analysis of significantly enriched proteins in the ‘Heatin-eluted’ fraction based on their molecular function.** Shown are molecular function categories, the number of proteins within the category, the number of proteins mapped to the category, the expected number of proteins mapped to the category, the fold enrichment, the significance of enrichment; p-value (Fisher‘s Exact test), the protein names with corresponding AGI codes. See also Figure S10a. Results with a Bonferroni-corrected p-value < 0.05 and a fold enrichment > 1 are displayed.

| **GO molecular function category** | **# in category** | **# Mapped IDs** | **expected** | **Fold Enrichment** | **pvalue** | **Mapped proteins** | **AGI code** |
| --- | --- | --- | --- | --- | --- | --- | --- |
| phospholipase activator activity | 6 | 6 | 0.05 | > 100 | 5.15E-07 | ADP-ribosylation factor 1 | AT2G47170 |
|  |  |  |  |  |  | ADP-ribosylation factor 2-A | AT1G70490 AT1G23490 |
|  |  |  |  |  |  | ADP-ribosylation factor 2-B | AT1G70490 AT1G23490 |
|  |  |  |  |  |  | ADP-ribosylation factor A1B | AT5G14670 |
|  |  |  |  |  |  | ADP-ribosylation factor A1E | AT3G62290 |
|  |  |  |  |  |  | ADP-ribosylation factor A1F | AT1G10630 |
| **indole-3-acetonitrile nitrile hydratase activity** | **4** | **3** | **0.03** | **88.02** | **3.46E-02** | **Nitrilase 1** | **AT3G44310** |
|  |  |  |  |  |  | **Nitrilase 2** | **AT3G44300** |
|  |  |  |  |  |  | **Nitrilase 3** | **AT3G44320** |
| **indole-3-acetonitrile nitrilase activity** | **4** | **3** | **0.03** | **88.02** | **3.46E-02** | **Nitrilase 1** | **AT3G44310** |
|  |  |  |  |  |  | **Nitrilase 2** | **AT3G44300** |
|  |  |  |  |  |  | **Nitrilase 3** | **AT3G44320** |
| ribulose-bisphosphate carboxylase activity | 4 | 3 | 0.03 | 88.02 | 3.46E-02 | Ribulose bisphosphate carboxylase large chain | ATCG00490 |
|  |  |  |  |  |  | Ribulose bisphosphate carboxylase small chain 1A, chloroplastic | AT1G67090 |
|  |  |  |  |  |  | Ribulose bisphosphate carboxylase small chain 2B, chloroplastic | AT5G38420 |
| thioredoxin peroxidase activity | 10 | 4 | 0.09 | 46.95 | 7.93E-03 | Peroxiredoxin-2B | AT1G65980 |
|  |  |  |  |  |  | Peroxiredoxin-2E, chloroplastic | AT3G52960 |
|  |  |  |  |  |  | Peroxiredoxin Q, chloroplastic | AT3G26060 |
|  |  |  |  |  |  | 2-Cys peroxiredoxin BAS1, chloroplastic | AT3G11630 |
| proton-transporting ATPase activity, rotational mechanism | 22 | 5 | 0.19 | 26.67 | 4.87E-03 | ATP synthase subunit alpha, chloroplastic | ATCG00120 |
|  |  |  |  |  |  | V-type proton ATPase catalytic subunit A | AT1G78900 |
|  |  |  |  |  |  | V-type proton ATPase subunit C | AT1G12840 |
|  |  |  |  |  |  | V-type proton ATPase subunit E1 | AT4G11150 |
|  |  |  |  |  |  | V-type proton ATPase subunit E3 | AT1G64200 |
| chlorophyll binding | 34 | 5 | 0.29 | 17.26 | 3.20E-02 | Light-harvesting complex-like protein 3 isotype 1, chloroplastic | AT4G17600 |
|  |  |  |  |  |  | Chlorophyll a-b binding protein CP29.2, chloroplastic | AT3G08940 |
|  |  |  |  |  |  | Photosystem I chlorophyll a/b-binding protein 2, chloroplastic | AT3G61470 |
|  |  |  |  |  |  | Photosystem II 22 kDa protein, chloroplastic | AT1G44575 |
|  |  |  |  |  |  | Photosystem II protein D1 | ATCG00020 |
| nutrient reservoir activity | 55 | 7 | 0.47 | 14.94 | 1.59E-03 | 12S seed storage protein CRA1 | AT5G44120 |
|  |  |  |  |  |  | 12S seed storage protein CRB | AT1G03880 |
|  |  |  |  |  |  | 12S seed storage protein CRC | AT4G28520 |
|  |  |  |  |  |  | 12S seed storage protein CRD | AT1G03890 |
|  |  |  |  |  |  | Vicilin-like seed storage protein At2g28490 | AT2G28490 |
|  |  |  |  |  |  | Vicilin-like seed storage protein At3g22640 | AT3G22640 |
|  |  |  |  |  |  | Temperature-induced lipocalin-1 | AT5G58070 |
| copper ion binding | 190 | 12 | 1.62 | 7.41 | 2.86E-04 | 20 kDa chaperonin, chloroplastic | AT5G20720 |
|  |  |  |  |  |  | Adenosine kinase 1 | AT3G09820 |
|  |  |  |  |  |  | Adenosine kinase 2 | AT5G03300 |
|  |  |  |  |  |  | ADP-ribosylation factor A1F | AT1G10630 |
|  |  |  |  |  |  | Beta-glucosidase 23 | AT3G09260 |
|  |  |  |  |  |  | Bifunctional enolase 2/transcriptional activator | AT2G36530 |
|  |  |  |  |  |  | Elongation factor 2 | AT1G56070 |
|  |  |  |  |  |  | Probable elongation factor 1-gamma 2 | AT1G57720 |
|  |  |  |  |  |  | PYK10-binding protein 1 | AT3G16420 |
|  |  |  |  |  |  | Ribosome-recycling factor, chloroplastic | AT3G63190 |
|  |  |  |  |  |  | Ribulose bisphosphate carboxylase small chain 1A, chloroplastic | AT1G67090 |
|  |  |  |  |  |  | Triosephosphate isomerase, cytosolic | AT3G55440 |

**Table S12.** **Primers used in this study.** Left, right and insertion primers are given for genotyping primers. Forward and reverse are given for RT-PCR primers. Tm denote the used annealing temperature.

| **Primers set** | **Left Primer** | **Right Primer** | **Insertion primer** | **Tm** |
| --- | --- | --- | --- | --- |
| *SALK_011511* | TGAGGTAGTGATGGAACAGCC | GTGAGAAAGCGATTGACGAAG | ATTTTGCCGATTTCGGAAC (LBb1.3) | 57°C |
| *SALK_152424* | AACAGGTGATGATGGTGAAGC | CTCTGCTTCCACAACCGTAAG | ATTTTGCCGATTTCGGAAC (LBb1.3) | 60°C-56°C |
| *SALK_104895* | ACTGCATGGGAGTGTCTTTTG | GAGGTTTTGGAGGGAAATCTG | ATTTTGCCGATTTCGGAAC (LBb1.3) | 60°C-58°C,  53°C-50°C |
| *GABI_379H03* | TTAACAGCCTGTCTGGTAGCG | TTGGCGTATCTTCCAAGTTTG | GTGGATTGATGTGATATCTCC (o3144/35St) | 54°C |
| *SALK_072361* | TTCTATTGGAAATGCATTGCC | TAAAACATCGGATGAACCTCG | ATTTTGCCGATTTCGGAAC (LBb1.3) | 59°C-50°C |
| *SAIL_78_H09* | TTTCTTGAATTGTCTCCGGTG | TAAAACATCGGATGAACCTCG | TAGCATCTGAATTTCATAACCAATCTCGATACAC (LB3) | 54°C |
| *SALK_047520* | AATTAGTTGTTGGCAACACGG | ATGCTCCATGTAGACAATGGG | ATTTTGCCGATTTCGGAAC (LBb1.3) | 60°C-50°C |
| *SALK_057531* | ATGCTCCATGTAGACAATGGG | AATTAGTTGTTGGCAACACGG | ATTTTGCCGATTTCGGAAC (LBb1.3) | 51°C, 60°C |
| *SALK_099479* | ATTTCGGATCCCATCAAAATC | AAGCTATACTCGCGAAGCTCC | ATTTTGCCGATTTCGGAAC (LBb1.3) | 58°C |
| **Primers set** | **Forward Primer** | **Reverse Primer** |  |  |
| *AAO1-1* | AAGCTTGGTTGTGGCGAAGGTG | TTCTTCGCGGAGTGCCAAGATG |  | 57°C |
| *AAO1-2* | GCCGTGAAATCAATGCCTGTTGC | TCCCGGTGTTGATCGCATGTTC |  | 57°C |
| *AAO2-1* | AGTCGAAGATTTCACTGTGAGC | ATCAAACCGAGTTAAACTACTAGAATC |  | 57°C |
| *AAO2-2* | GAATGCATCGCCTTCGTGGTTG | ACAGGACGCTGCAGTTTCTTAGC |  | 57°C |
| *AAO2-3* | TGTGGAGAAAACGAGGGATA | TCAGGTACTGCATGGGAGTG |  | 57°C |
