# Supplementary material for: The chemical compound ‘Heatin’ stimulates hypocotyl elongation and interferes with the Arabidopsis NIT1‐subfamily of nitrilases
Source: Plant J. 2021 May 6;106(6):1523–40. doi: 10.1111/tpj.15250 (PMC8360157; doi:10.1111/tpj.15250)
Supplement: Supplementary file 3 — Appendix S1. Transcriptomics of Heatin responsiveness. Appendix S2. Chemical synthesis of azide‐functionalized probe (N‐(6‐azidohexyl)‐4‐((benzyl((2‐hydroxynaphthalen‐1 yl)methyl)amino)methyl)benzamide). Appendix S3. Heatin stimulates growth of Brassicaceae varieties specifically. Appendix S4. Chemical synthesis of Heatin (Ethyl naphthalen‐1‐ylalaninate). [file TPJ-106-1523-s004.docx]

**Appendix S1.**

**Transcriptomics of Heatin responsiveness**

An early (two day-old seedlings; 48 h) and late (seven day-old seedlings; 168 h) sampling time point for RNA-sequencing experiments were defined based on the establishment of a detailed profile of hypocotyl growth from imbibed seed to eight day-old seedling demonstrated in the presence and absence of Heatin (Figure S7, S8, S9a).

High temperature (27°C) had a profound impact on the transcriptome, as in two day-old and seven day-old seedlings 4140 and 2463 genes, respectively, were significantly differentially expressed at least two-fold compared to control temperature (22°C) (Table S5). 49.8% of these genes were upregulated and 50.2% downregulated in two day-old seedlings and 41.9% upregulated vs. 58.1% downregulated in seven day-old seedlings (Table S5, Figure S9b).

The large number of high temperature-regulated genes was in stark contrast with the transcriptional response to Heatin application (Table S5). In two day-old seedlings, the expression of only 2 genes was significantly different at 22°C and 10 genes at 27°C (Table S6). None were left when a 2-fold change threshold was considered at 22°C and only 6 remained at 27°C. The Heatin-transcriptome was nevertheless more pronounced in seven day-old seedlings, where 406 genes were significantly regulated at 22°C and 606 genes at 27°C. Yet only 49 (22°C) and 193 (27°C) genes were left when the 2-fold change cut-off was considered (Table S5), despite the clear stimulating effect of Heatin on hypocotyl elongation (Figure S9a).

Of the 406 and 606 Heatin-regulated genes in seven day-old seedlings grown at 22°C and 27°C, 219 and 291, respectively, were upregulated, whereas 187 and 315 were downregulated (Table S5). Approximately 29% (63/219) of the upregulated genes at 22°C were also among the 291 upregulated genes at 27°C (Figure S9b). Considering the downregulated genes at 22°C, 18% (34/187) were also among the 315 downregulated genes at 27°C. Two genes were upregulated at 22°C and downregulated at 27°C and one gene was downregulated at 22°C and upregulated at 27°C (Figure S9b). Together, the transcriptome analyses showed little overlap in the response to Heatin at the two tested temperatures, indicating a strong interaction between compound and temperature.

A large fraction (56.6%; 124/219) of the genes that were upregulated in response to Heatin at control temperature (22°C), were also upregulated by high temperature conditions (27°C) in the absence (mock) of Heatin (Figure S9c). Genes induced by Heatin at 27°C showed 28.2% (82/291) overlap with the mock high temperature transcriptome. Among the downregulated genes, a similar trend was observed with 71.7% (134/187) overlap at 22°C and 33.7% (106/315) at 27°C (Fig. 9d). 31 and 21 genes were found to be shared by all three groups of upregulated respectively downregulated genes (Figure S9c,d, Table S7). Of the 31 genes with upregulated expression in all 3 comparisons, we consistently found the highest RNA-seq read counts in seedlings that were grown in the presence of Heatin at warm temperatures (Table S7). This is in line with hypocotyl length observations of these three groups (Figure 1a), suggesting that these genes could be tightly linked with the observed phenotype. Downregulated genes showed a similar trend, where 18 of the 21 overlapping genes had the lowest RNA-seq read count in Heatin-treated samples grown at 27°C (Table S7).

**Appendix S2.**

**Chemical synthesis of azide-functionalized probe (N-(6-azidohexyl)-4-((benzyl((2-hydroxynaphthalen-1-yl)methyl)amino)methyl)benzamide)**

All starting materials, reagents and solvents used for probe synthesis were obtained from commercial vendors and used without further purification. The 4-(chloromethylbenzoyl)chloride used in the synthesis of compound **3** was purchased from Sigma-Aldrich. Dry solvents were dried over 4A or 3A molecular sieves. Thin-layer chromatography (TLC) spots were visualized under 254 and 356 nm and by staining with ninhydrine, triphenylphosphine/ninhydrine and phosphomolybdic acid. Nuclear magnetic resonance (NMR) spectroscopy was performed on a 400 MHz Varian NMR machine.

**1,6-diazidohexane (1)**

To a solution of 1,6-dibromohexane (8.00 g, 32.8 mmol) in 50 ml of dry N,N-dimethylformamide (DMF) was added sodium azide (6.40 g, 3 eq), and the reaction mixture was stirred for 14 h at 60°C. The mixture was concentrated in vacuum, diluted with Et2O, extracted with saturated NaHCO3 and water. The water layer was extracted with Et2O, the organic layers combined, dried with Na2SO4, concentrated in vacuum and purified by column chromatography (Petroleum ether (PE), then PE:EtOAc 97:3) yielding 1,6-diazidohexane as a colorless oil (4.30 g, 78%). **Analytical data:** *Rf* 0.6 (PE/EtOAc 97:3); 1H NMR (400 MHz, CDCl3) δ 3.26 (t, 4H), 1.60 (m, 4H), 1.39 (m, 4H).

**1-aminohexyl-6-azide (2)**

To a solution of 1,6-diazidohexane (4.30 g, 25.6 mmol) in a mixture of Et2O (25 ml) and EtOAc (25ml) was added 1M HCl (40ml). The mixture was cooled to 0°C and triphenylphosphine (3.36 g, 12.8 mmol) was added in portions over 15 minutes. The mixture was stirred vigorously for4 h. The layers were separated, and the aqueous layer was extracted with 2x 20 ml Et2O. The pH of the aqueous layer was adjusted with 4M NaOH until pH 12 and was extracted 3x with 50 mL Et2O. The combined organic layers were dried with Na2SO4, concentrated in vacuum and purified by column chromatography (Dichloromethane (DCM)/MeOH/NH4OH 9:1:0.1 yielding 1-aminohexyl-6-azide as a colorless oil (2.40 g, 65%). **Analytical data:** 1H NMR (400 MHz, CDCl3) δ 3.24 (t, J = 6.9 Hz, 2H), 2.68 (t, J = 7.0 Hz, 2H), 1.59 (m, J = 10.7, 6.9 Hz, 3H), 1.50 – 1.25 (m, 7H).

**N-(6-azidohexyl)-4-(chloromethyl)benzamide (3)**

1-aminohexyl-6-azide (1.26 g, 8.86 mmol) was dissolved in dry DCM and cooled to 0°C. DMAP (217 mg, 1.77 mmol, 0.2 eq.), pyridine (1.30 ml, 17.7 mmol, 2 eq.) and 4-(chloromethylbenzoyl)chloride (1.68 g, 8.86 mmol, 1 eq.) were added and the mixture was stirred for 16 h at room temperature (RT). The reaction was quenched with satd. aq. NH4Cl. The layers were separated, and the aqueous layer was back-extracted with 2x 25 ml DCM. The organic layers were combined, dried with Na­2SO4 and concentrated in vacuuum. After column chromatography (9:1 DCM/MeOH) N-(6-azidohexyl)-4-(chloromethyl)benzamide was obtained as a yellowish solid (1.38 g, 53 %). **Analytical data:** 1H NMR (400 MHz, CDCl3) δ 7.79 – 7.66 (d, 2H), 7.49 – 7.37 (d, 2H), 6.17 (s, 1H), 4.58 (s, 1H), 3.50 – 3.36 (q, 2H), 3.25 (t, 2H), 1.68 – 1.49 (m, 4H), 1.49 – 1.30 (m, 4H).

**N-(6-azidohexyl)-4-(iodomethyl)benzamide (4)**

N-(6-azidohexyl)-4-(chloromethyl)benzamide (1.00 g, 3,39 mmol) was dissolved in 50 ml dry acetone. NaI (863 mg, 5.76 mmol, 1.7 eq) was added and the mixture was stirred overnight at 60°C. The reaction mixture was concentrated in vacuum. Et2O (100 ml) was added and washed twice with H2O and once with 1M sodium thiosulphate. The aqueous layers were back-extracted with Et2O (2x20 mL), the organic layers combined, dried with Na­2SO4 and concentrated in vacuum yielding N-(6-azidohexyl)-4-(iodomethyl)benzamide ( 1.24 g, 95%) as a yellowish crystalline solid which was used without further purification.

***tert*-butyl benzylcarbamate (5)**

Benzylamine (2,2 ml, 20 mmol) and triethylamine (4.2 ml, 1.5 eq) were dissolved in 50 ml dry DCM. Di-*tert*-butyl dicarbonate (5.24 g, 24 mmol, 1.2 eq) was added in portions at RT. The resulting reaction mixture was stirred at RT for 16H, diluted with Et2O and washed with 0.1M HCl and brine. The organic layers were dried with Na2SO4 and concentrated in vacuum. Column chromatography (9:1 PE/EtOAc) yielded white crystals of tert-butyl benzylcarbamate (1.5 g, 36%). **Analytical data:** 1H NMR (400 MHz, CDCl3) δ 7.40 – 7.15 (m, 5H), 4.81 (s, 1H), 4.24 (t, 2H), 1.45 (s, 9H).

**tert-butyl (4-((6-azidohexyl)carbamoyl)benzyl)(benzyl)carbamate (6)**

tert-butyl benzylcarbamate (216 mg, 2.41 mmol) was dissolved in dry DMF and cooled to 0°C. NaH (60% in paraffin) (440 mg, 26.5 mmol, 4.5 eq) was added and the reaction mixture was allowed to warm to RT in 30 min. N-(6-azidohexyl)-4-(iodomethyl)benzamide (400 mg, 2.41 mmol, 1 eq) was added and the reaction mixture was stirred at RT for 16 h. Water was added to quench the excess NaH and the mixture was concentrated in vacuum. The residue was dissolved in DCM and washed with H2O. The aqueous layer was back-extracted with DCM, the organic layers combined, dried with Na­2SO4 and concentrated in vacuum. The residue was purified by column chromatography (1:1 PE/ EtOAc) yielding tert-butyl (4-((6-azidohexyl)carbamoyl)benzyl)(benzyl)carbamate ( 336 mg, 30 %) as a waxy solid.

**Analytical data:** 1H NMR (400 MHz, CDCl3) δ 7.70 (d, 2H), 7.36 – 7.10 (m, 7H), 6.15 (s, 1H), 4.52 – 4.21 (m, 4H), 3.50 – 3.34 (q, 2H), 3.25 (t, 2H), 1.76 – 1.53 (m, 5H), 1.53 – 1.09 (m, 12H), MS (ESI) found 931.30 (2M+H)+

**N-(6-azidohexyl)-4-((benzyl((2-hydroxynaphthalen-1-yl)methyl)amino)methyl)benzamide (7)**

tert-butyl (4-((6-azidohexyl)carbamoyl)benzyl)(benzyl)carbamate (256 mg, 0.55 mmol) was dissolved in 20ml dry DCM and cooled to 0°C. 10 ml Trifluoroacetic acid (TFA) was added and the mixture was stirred for 2H. After TLC indicated completion, the mixture was concentrated in vacuum, co-evaporated with CHCl3 and used without further purification. The amine (200 mg, 0.55 mmol) was dissolved in 1,2-dichloroethane. 3 Å molecular sieves were added, followed by 2-hydroxy-1-naphthaldehyde (190 mg, 1.1 mmol, 2 eq) and sodium triacetoxyborohydride (580 mg, 2.75 mmol, 5 eq). The mixture was stirred at RT for 14 H, filtered and concentrated in vacuum. The residue was partitioned between EtOAc and satd. NaHCO3 and the aqueous layers were back extracted with EtOAc. The organic layers were dried with Na2SO4, concentrated in vacuum and purified by column chromatography (2:1 PE/EtOAc) and then preparative HPLC yielding N-(6-azidohexyl)-4-((benzyl((2-hydroxynaphthalen-1-yl)methyl)amino)methyl) benzamide as a colorless oil (33 mg, 3.5 %). **Analytical data:** 1H NMR (400 MHz, CDCl3) δ 7.81-7.67 (m, 5H), 7.66 – 7.51 (m, 1H), 7.50 – 7.41 (m, 1H), 7.41 – 7.26 (m, 7H), 7.21 – 7.12 (d, 1H) 6.33 – 6.14 (t, 1H), 4.35 – 4.20 (s, 2H), 4.00 – 3.80 (d, 4H), 3.51 – 3.34 (q, 2H), 3.34 – 3.11 (t, 2H), 1.68 – 1.49 (m, 4H), 1.49 – 1.30 (m, 4H). HRMS (ESI) calcd. for C32H36N5O2 [M+H]+ 522.28690, found 522.2864

**Compound 1: 1H NMR (400 MHz, CHCl3)**

**Compound 2: 1H NMR (400 MHz, CHCl3)**

**Compound 3: 1H NMR (400 MHz, CHCl3)**

**Compound 5: 1H NMR (400 MHz, CHCl3)**

**Compound 6: 1H NMR (400 MHz, CHCl3)**

**Compound 7: 1H NMR (400 MHz, CHCl3)**

**Appendix S3.**

**Heatin stimulates growth of *Brassicaceae* varieties specifically.**

In an agricultural setting, Heatin might be applicable for priming of crops for thermomorphogenesis, potentially increasing crop survival and production under otherwise high temperature conditions. Therefore, to exclude that Heatin effects are specific for Arabidopsis and to test the lead that Heatin may target the *Brassicaceae-*specific NIT1-subfamily of Nitrilases, we quantified Heatin effects on hypocotyl elongation of several commercial crop varieties (Figure S10b). We applied 25 µM Heatin, at control and high temperature conditions (22°C and 27°C) to the crop varieties: *Brassica oleracea* var. *Italica* (broccoli), *Raphanus raphanistrum* subsp. *Sativus* (radish), *Brassica oleracea* var. *gemmifera* (Brussels sprouts) and *Brassica rapa* subsp. *pekinensis* (Chinese cabbage), next to non-*Brassicaceae* crops: *Lactuca sativa* (Butter lettuce and Batavia lettuce), *Solanum lycopersicum* (tomato) and *Capsicum annuum* (chili pepper and bell pepper). For broccoli, radish, Brussels sprouts, Chinese cabbage and tomato ‘Low Seed Count’ (LSC) and ‘High Seed Count’ (HSC) seed batches were included, which had a relatively low or relatively high number of seeds per gram respectively, which is directly related to seed size.

Significant hypocotyl elongation in response to Heatin was observed in broccoli, Brussels sprouts and Chinese cabbage (Figure 10b). In broccoli, we observed clear Heatin-induced hypocotyl elongation in the LSC seed batch under both temperatures and in HSC seed batch only in high temperature conditions. In Brussels sprouts, Heatin sensitivity was observed in the HSC seed batch under both temperatures and in Chinese cabbage in the LSC seed batch under high temperature conditions only. Strikingly, all the non-*Brassicacea* crops in this experiment (butter lettuce, tomato, chili pepper and bell pepper), did not respond to Heatin under either temperature condition. These results strongly suggest that Heatin activity potential extends beyond Arabidopsis but is likely restricted to the *Brassicaceae* family. However, not all members of this family are sensitive, as we found no response in radish (Figure S10b). Additionally, high temperature seems to affect Heatin sensitivity as broccoli HSC and Chinese cabbage LSC are only sensitive to the compound under high temperature conditions.

**Appendix S4. Chemical synthesis of Heatin (Ethyl naphthalen-1-ylalaninate)**

**ethyl naphthalen-1-ylalaninate (1)**

2-napthylamine (2.86 g, 20.00 mmol) and triethylamine (7.00 ml, 2.5 eq.) were dissolved in 30 ml dry DMF. Ethyl-2-bromopropionate (2.85 ml, 1.1 eq.) was added and the mixture was heated to 60°C and stirred for 14H. The mixture was concentrated in vacuo, the residue redissolved in DCM, extracted with H2O. The organic layer was dried with Na2SO4, concentrated in vacuo and purified by column chromatography (4:1 hexanes/EtOAc) yielding ethyl naphthalen-1-ylalaninate (3.94 g, 80%).

**Analytical data:** 1H NMR (400 MHz, CDCl3) δ 7.89 (m, 1H), 7.83 – 7.71 (m, 1H), 7.52 – 7.37 (m, 2H), 7.35 – 7.21 (m, 2H), 6.53 (d, 1H), 4.94 (d, 1H), 4.40 – 4.26 (m, 1H), 4.26 – 4.11 (m, 2H), 1.59 (m, 3H), 1.26 (m, 3H).

**2-(naphthalen-1-ylamino)propanehydrazide (2)**

Ethyl naphthalen-1-ylalaninate (4,00 g, 16,44 mmol) was dissolved in 30 ml of ethanol. Hydrazine hydrate (8.4 ml, 10 eq.) was added and the mixture was heated to reflux for and stirred for 3H. The mixture was concentrated in vacuo, redissolved in DCM, extracted with H2O, the organic layer dried with Na2SO4, concentrated in vacuo and purified by column chromatography (97:3 DCM/MeOH) yielding 2-(naphthalen-1-ylamino)propanehydrazide (3.24 g, 86%).

**Analytical data:** 1H NMR (400 MHz, CD3OD) δ 8.06 (dd, 1H), 7.69 (dd, 1H), 7.57 – 7.33 (m, 2H), 7.33 – 7.03 (m, 2H), 6.48 (t, 1H), 4.19 – 3.94 (m, 1H), 1.54 (m, 3H).

**N'-((2-hydroxynaphthalen-1-yl)methylene)-2-(naphthalen-1-ylamino)propanehydrazide (3)**

2-(naphthalen-1-ylamino)propanehydrazide ( 2.8 g, 12.21 mmol) was dissolved in ethanol and 1-hydroxy-2-naphthaldehyde (2.09 g, 1 eq.) was added. The reaction mixture was refluxed for 2H and the yellowish precipitate was collected by vacuum filtration, yielding N'-((2-hydroxynaphthalen-1-yl)methylene)-2-(naphthalen-1-ylamino)propanehydrazide (4.12 g, 88%). 1H NMR spectrum matches the commercially available compound.

**Analytical data:** 1H NMR (400 MHz, DMSO*-d6*) δ 9.28 (s, 1H), 8.93 (s, 1H), 8.80 (d, , 1H), 8.42 – 8.25 (m, 1H), 8.17 (d, 1H), 7.85 (dd, 2H), 7.78 – 7.67 (m, 1H), 7.63 – 6.99 (m, 7H), 6.46 (d, 1H), 6.39 – 6.14 (m, 1H), 4.99 (dd, 1H), 4.19 (m, 1H), 1.60 (m, 3H).

**ethyl naphthalen-1-ylalaninate 1H NMR (400 MHz, CDCl3) (1)**

**2-(naphthalen-1-ylamino)propanehydrazide 1H NMR (400 MHz, CD3OD)** **(2)**

**N'-((2-hydroxynaphthalen-1-yl)methylene)-2-(naphthalen-1-ylamino)propanehydrazide
1H NMR (400 MHz, DMSO-*d6*) (3)**
